# On the evaluation of outlier detection and one-class classification: a comparative study of algorithms, model selection, and ensembles

**DOI:** 10.1007/s10618-023-00931-x

**Published:** 2023-05-16

**Authors:** Henrique O. Marques, Lorne Swersky, Jörg Sander, Ricardo J. G. B. Campello, Arthur Zimek

**Affiliations:** 1 University of Southern Denmark, Odense, Denmark; 2 University of Alberta, Edmonton, Canada

**Keywords:** Semi-supervised learning, One-class classification, Outlier detection, Evaluation, Model selection, Ensembles

## Abstract

It has been shown that unsupervised outlier detection methods can be adapted to the one-class classification problem (Janssens and Postma, in: Proceedings of the 18th annual Belgian-Dutch on machine learning, pp 56–64, 2009; Janssens et al. in: Proceedings of the 2009 ICMLA international conference on machine learning and applications, IEEE Computer Society, pp 147–153, 2009. 10.1109/ICMLA.2009.16). In this paper, we focus on the comparison of one-class classification algorithms with such adapted unsupervised outlier detection methods, improving on previous comparison studies in several important aspects. We study a number of one-class classification and unsupervised outlier detection methods in a rigorous experimental setup, comparing them on a large number of datasets with different characteristics, using different performance measures. In contrast to previous comparison studies, where the models (algorithms, parameters) are selected by using examples from both classes (outlier and inlier), here we also study and compare different approaches for model selection in the absence of examples from the outlier class, which is more realistic for practical applications since labeled outliers are rarely available. Our results showed that, overall, SVDD and GMM are top-performers, regardless of whether the ground truth is used for parameter selection or not. However, in specific application scenarios, other methods exhibited better performance. Combining one-class classifiers into ensembles showed better performance than individual methods in terms of accuracy, as long as the ensemble members are properly selected.

**Supplementary Information:**

The online version contains supplementary material available at 10.1007/s10618-023-00931-x.

## Introduction

Outlier detection is one of the central tasks of data mining. This task is aimed at identifying observations that deviate substantially from the remaining data. Many definitions of outlier exist in the literature (Zimek and Filzmoser 2018). One of the most cited ones is Hawkins’ definition (Hawkins 1980), which refers to an outlier as “*an observation which deviates so much from other observations as to arouse suspicions that it was generated by a different mechanism*”. Detecting such patterns is important because they might represent extraordinary behaviors that deserve special attention. In the literature, there are different names to refer to the task of detecting such extraordinary patterns, e.g., anomaly detection, novelty detection, noise detection, deviation detection, or exception mining (Zimek and Filzmoser 2018). Although sometimes these terms are used interchangeably, the name given to such a pattern usually depends on the application scenario. The range of real-world applications is diverse and includes, e.g., intrusion detection (Kwon et al. 2019), fraud detection (Adewumi and Akinyelu 2017), video surveillance (Chong and Tay 2017), industrial anomalies detection (Ramotsoela et al. 2018), medical anomaly detection (Litjens et al. 2017; Alaverdyan et al. 2020), trend detection (Schubert et al. 2015), and bot detection (Rodríguez-Ruiz et al. 2020; Bezerra et al. 2019). Further real-world applications have been discussed, e.g., by Pimentel et al. (2014), Chandola et al. (2009), and Chalapathy and Chawla (2019).

Outlier detection algorithms can be categorized into supervised, semi-supervised, and unsupervised techniques (Han et al. 2011). Supervised techniques can be seen as a special case of binary classification, when there are enough observations labeled as inliers and outliers available to train a classifier. Due to the natural imbalance between inliers and outliers, techniques for imbalanced classification can be used to train these classifiers (Aggarwal 2013). In semi-supervised outlier detection there are not enough (or no) outlier observations available to sufficiently describe the outlier class, due to the rarity of outliers. In this scenario, also referred to as “novelty detection”, the model is typically obtained using one-class classification techniques (Pimentel et al. 2014; Khan and Madden 2014). When no labeled data are available, we can use unsupervised techniques that do not assume any prior knowledge about which observations are outliers and which are inliers (Tan et al. 2005).

In this paper, we focus on one-class classification methods and unsupervised outlier detection methods adapted to the problem of novelty detection. Following the approach proposed by Janssens and Postma (2009), unsupervised outlier detection methods can be extended to use inlier class information to be applicable also in the semi-supervised setting. Janssens et al. (2009) performed a comparative study between 3 methods proposed for one-class classification [kNN Data Description (de Ridder et al. 1998), Parzen Windows (Parzen 1962) and SVDD (Tax and Duin 2004)] and 2 methods originally proposed for unsupervised outlier detection [LOF (Breunig et al. 2000) and LOCI (Papadimitriou et al. 2003)] extended to the one-class classification scenario. The authors concluded that LOF and SVDD were the top two performers and that their difference in performance was not statistically significant.

In our previous, preliminary study (Swersky et al. 2016), we performed a more comprehensive investigation than Janssens et al. (2009), using a more rigorous experimental setup, comparing the methods on a larger number of datasets with different characteristics, and using different performance measures. Our experiments led to conclusions that do not fully agree with those reported by Janssens et al. (2009). In particular, we could not confirm the same set of methods as performing best among the methods included in Janssens et al. (2009). We also identified some methods as top-performers in our experiments that were not included in their study.

In this paper, which is a major extension of our preliminary publication (Swersky et al. 2016), we aim to step forward and provide a more comprehensive study by making the following additional contributions:

- We increase the number of base datasets used in the evaluation from 30 to 50. The introduction of these new datasets allows us to draw stronger conclusions (from a statistical point of view) and study new scenarios, such as scenarios involving local outliers (as opposed to outlier vs. inlier classes only), low and high dimensional data spaces, as well as smaller and larger sample sizes.
- We include 4 additional well-known algorithms in the comparison: Isolation Forest (iForest) (Liu et al. 2008, 2012), Gaussian Mixture Model (GMM) (Bishop 2007), Subspace Outlier Degree (SOD) (Kriegel et al. 2009), and Deep SVDD (Ruff et al. 2018). Some of these algorithms are shown to be among the top performers in specific application scenarios.
- In addition to ROC AUC and AjustedPrec@*n* used in our previous study, we also use the Matthews Correlation Coefficient (MCC) (Matthews 1975) to measure the performance of the methods. While ROC AUC and AjustedPrec@*n* evaluate the rankings produced by the methods, MCC evaluates the labelings.
- In contrast to our previous study, where we only selected the models (algorithms, parameters) based on the ground truth by using examples from both classes (outlier and inlier), here we also study and compare different approaches for model selection in the absence of examples for the outlier class. The study of these approaches is very important from a practical perspective, since representative examples of outliers are often not available in real-world applications of one-class classification.
- We consider ensembles of one-class classifiers (Tax and Duin 2001a; Krawczyk and Cyganek 2017; Hempstalk et al. 2008; Krawczyk and Woźniak 2014) in our extended study. In particular, while in the one-class classification literature only score-based strategies for building ensembles have been used, here we adopt a rank-based approach to build the ensembles. Rank-based strategies avoid the score normalization procedure required by score-based strategies, which can be notoriously problematic and challenging (Kriegel et al. 2011; Gao and Tan 2006; Azami et al. 2017).
- Finally, while most OCC ensemble approaches in the literature use ground truth (label) information to select ensemble members, here we explore base model selection both *with* and *without* ground truth information. To the best of our knowledge, this is the first work on one-class classification that allows selection of ensemble members to be performed without the requirement of ground truth information.

With these additional contributions, we aim to answer the following research questions:

- Among all the methods studied (7 algorithms proposed for one-class classification and 7 originally proposed for unsupervised outlier detection extended to one-class classification), how do they perform in different scenarios, namely, single/multiple source-class inliers, global/local outliers, low/high dimensional data spaces, and smaller/larger sample sizes? Is there any algorithm capable of consistently performing well in most scenarios?
- Given that labeled outliers are rarely available in practical applications, how well do the studied model selection methods perform in the absence of labeled outliers? Is there any method capable of consistently selecting good models/parameters in each of the different (or in most) scenarios? Do they work equally well for tuning the parameter values of the different algorithms studied?
- Given that a single model cannot capture all the aspects involved in the one-class classification problem, is combining one-class classifiers into ensembles an effective way to improve the performance and robustness of the results? How to effectively guide the selection of the base members of the ensembles in a practical manner?

The remainder of this paper is organized as follows. In Sect. 2, we discuss related work. In Sects. 3 and 4, we provide the reader with relevant background on one-class classification, unsupervised outlier detection, and model selection. We describe the setup of the experiments in Sect. 5 and discuss the results in Sect. 6. We summarize and conclude the paper in Sect. 7. Additionally, we provide Supplementary Material. Section 1 in the Supplementary Material provides the full description of all the methods compared in the experiments. In Section 2 in the Supplementary Material, we provide the detailed results of the experiments.

## Related work

### One-class classification versus unsupervised outlier detection

Both unsupervised outlier detection and one-class classification were first studied in the field of statistics (Barnett and Lewis 1994; Markou and Singh 2003). The one-class classification scenario generally is an easier task, because training data for one class is available. While in the one-class classification setting one can estimate the probability density function (pdf) or other models without the presence of outliers in the dataset, in the unsupervised outlier detection setting one must deal with possible outliers while estimating the pdf or other models. Given the differences between these two settings, the corresponding methods cannot be compared in a straightforward way.

In the literature, there have been limited attempts to establish connections between, or compare the performance of, both categories of methods. One attempt to compare one-class classification and unsupervised outlier detection methods was reported by Hido et al. (2008). In that work, the authors compared their proposed outlier detection algorithm against other approaches, including supervised, semi-supervised and unsupervised outlier detection techniques. The comparison, however, was not entirely unbiased; for instance, the supervised and semi-supervised algorithms were trained with the best parameters selected using cross-validation, while only 3 different arbitrary values for LOF’s parameter were tested.

Janssens and Postma (2009) proposed a methodological framework to make unsupervised outlier detection algorithms work in a one-class classification setup and thus make them comparable to algorithms specifically designed for this task. The authors, however, only assessed the performance of two unsupervised methods, namely, LOF and LOCI. In a follow-up work (Janssens et al. 2009), the same framework was applied to LOF and LOCI, but this time these methods were also compared against three methods specifically designed for one-class classification. The authors concluded that LOF and SVDD were the top two performers with an identical average performance rank, although there were particular scenarios where each method could outperform the other.

Although Janssens’ proposal compares the methods in a fair setup, their experimental design was restricted to controlled experiments, where the performance of the algorithms is compared based on the selection of their parameters using a previously labeled dataset with representative examples from both classes. However, in practical applications where ground truth information is missing (or very limited) for a given class, it is difficult to select the best parameter(s), as only labeled examples of one of the classes are available.

### Semi-supervised model selection for one-class classification

The one-class classification model parameters typically determine how tightly the model should fit around the data, i.e., how complex the model should be. The more complex the model, the more flexible and tighter it fits the inlier class. Higher model complexity, however, comes at the price of overfitting risk, where the model may adapt to the specific instances from the inlier class, resulting in a high variance model. On the other hand, a model that is too simple can have high bias and lead to underfitting. Although suitable values for model parameters can lead to better fitting trade-offs, how to set these values is often not intuitive and the decision is typically left to the user. The selection of parameter values, however, has a major influence on the final performance of the models. In one-class classification, these parameters determine the trade-off between false positives and false negatives (Ruff et al. 2020; Juszczak 2006), such that poor parameter choices lead to poor trade-offs and, accordingly, limited classification performances.

In order to assist users to select the best parameterization for a given one-class classifier in real-world applications, some works in the literature have designed model selection methods for one-class classification that deal with the absence of representative examples for the outlier class. These works can be broadly categorized into methods designed for one specific type of algorithm (Duin 1976; Tax and Müller 2004; Xiao et al. 2015; Ghafoori et al. 2016), which aim to select the best value(s) for the parameter(s) of a given algorithm (e.g., the cross-validated likelihood (Smyth 2000) that can be used to select parameters for GMM); and methods designed for the selection of both the best algorithm and its parameterization (Tax and Duin 2001b; Wang et al. 2018). In this paper, we focus on the latter category of methods to select the best parameterization for different types of one-class classifiers.

### Ensembles

Several models have been proposed to solve the one-class classification problem. However, a single model cannot capture all the aspects involved in the one-class classification problem and, therefore, may fail in particular application scenarios. A possible approach to bypassing the selection of a single model and avoiding the selection of the weakest model, is to rely on an ensemble of multiple models in order to obtain more stable and accurate results. Ensemble methods have been studied for a variety of tasks, including ensembles for classification (Rokach 2010; Galar et al. 2012), regression (Mendes-Moreira et al. 2012), clustering (Zhou 2012; Strehl and Ghosh 2002), unsupervised outlier detection (Zimek et al. 2013a; Aggarwal and Sathe 2017), and one-class classification (Tax and Duin 2001a; Krawczyk and Cyganek 2017; Hempstalk et al. 2008; Krawczyk and Woźniak 2014).

There are two key properties that ensemble members for one-class classification should have: diversity and accuracy. Combining several but similar models would not add much information to a combined classifier. Similarly, combining diverse but very inaccurate classifiers could produce a weak ensemble.

There are typically two ways to introduce diversity into the ensembles. The first way is to build the ensemble using models with different biases (e.g. combining GMM, SVDD, and ANN) (Tax and Duin 2001a; Hempstalk et al. 2008). The second way is to build the ensemble using the same base model, but with different views of the data (e.g. using subsampling or subspaces) (Krawczyk and Cyganek 2017; Krawczyk et al. 2013).

To select accurate one-class classifiers, one typically must select both a method as well as the values for its parameters. In the literature, there have been two common approaches to select parameters for one-class classifiers, based on the relative comparison of the respective candidate models by measuring their accuracy using (a) examples from both classes, i.e., both ground truth inliers and ground truth outliers, or (b) by using only ground truth examples from the inlier class plus artificially generated outliers.

When building ensembles of one-class classifiers, the common approach in the literature is to use ground truth examples from both classes to select the ensemble members (Tax and Duin 2001a; Hempstalk et al. 2008), or to not perform a selection at all and instead use a set of fixed classifiers/parameters (Zhang et al. 2011). Here in this study we explore different approaches to perform ensemble member selection, first using both ground truth inliers and outliers, and then using ground truth inliers and artificially generated outliers only. To the best of our knowledge, this is the first work on one-class classification that selects the ensemble members using criteria that do not rely on the availability of ground truth examples from the outlier class.

Once one guarantees that the ensemble members are accurate and diverse enough, a third central question is how to combine them. A common approach is to combine the classification outcomes produced by multiple classifiers on some collection of test data. In general, these strategies can be divided into score-based strategies and rank-based strategies. *Score-based strategies* combine in some way the output scores associated with the test observations and produced from different classifiers to generate a new, consensus output score for each observation. One difficulty with this strategy is that the output domains of the different classifiers may vary. For example, one classifier might output a probability, while another might output a distance or a density measure. In order to combine the classifiers, some normalization procedure must be applied to the scores (Aggarwal and Sathe 2017; Kriegel et al. 2011). Most if not all works in the one-class classification literature rely on score-based strategies. The common approach is to transform the classifier output into posterior probabilities (Tax and Duin 2001a; Hempstalk et al. 2008) and combine them using different approaches that range from averaging the estimated posterior probabilities to more sophisticated combinations, such as voting or products (Tax and Duin 2001a; Zhang et al. 2011; Krawczyk et al. 2013; Krawczyk and Cyganek 2017; Spinosa and de Leon Ferreira de Carvalho 2005). *Rank-based strategies*, in contrast to the score-based strategies, ignore the actual scores and combine the rankings of the classifier outputs instead. Surprisingly, this type of strategy has not received much attention in the literature on ensembles of one-class classification, although it has been widely used in clustering (Jaskowiak et al. 2016) and unsupervised outlier detection (Zimek et al. 2013a, 2014). In this paper, we also investigate the use of a rank-based strategy for building ensembles of one-class classifiers.

## Outlier detection and one-class classification

### One-class classification

Unlike in the traditional supervised classification setting, in one-class classification (Tax 2001) we are only provided with observations from one class and our model must then classify new observations as belonging to this class or not.

In order to keep terminology consistent, we will refer to observations belonging to the provided class as inliers, and observations not belonging to this class as outliers.

One-class classifiers can be categorized into density methods, boundary methods, reconstruction methods, and deep learning methods.

#### Density methods

**Fig. 1:**
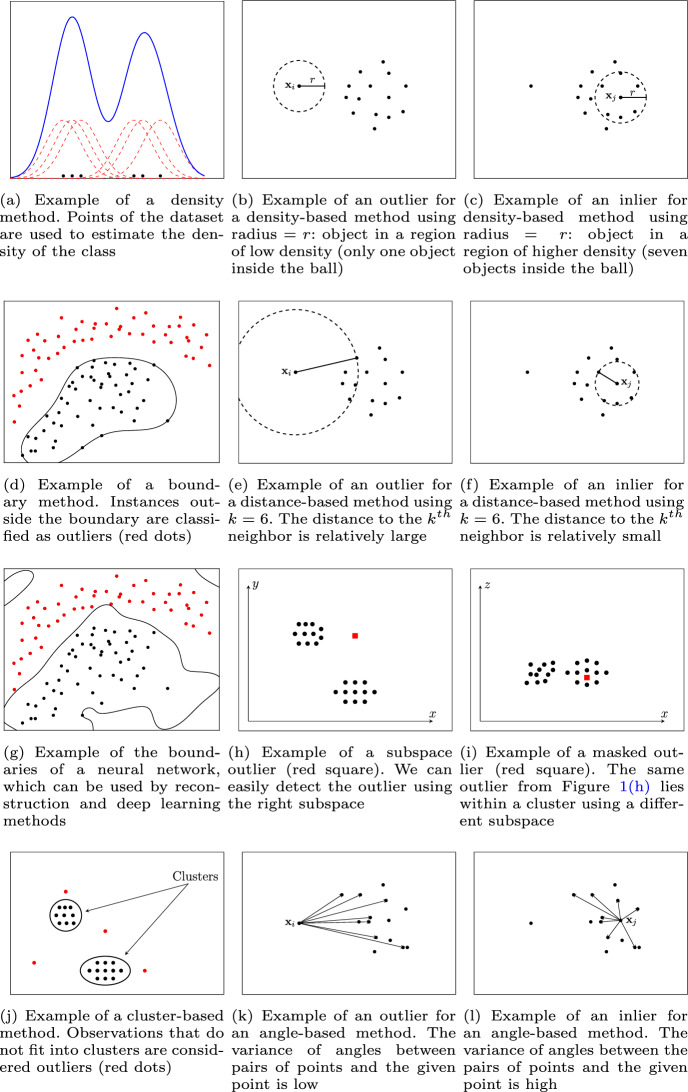
Examples of different approaches for one-class classification and unsupervised outlier detection

The density methods estimate the parameters for some probability density function (pdf) of the inlier class, given by the training data (Fig. 1a), and threshold this density. The new instances are classified using the threshold of the pdf. Since the training set usually contains only inliers, the pdf can be estimated without being affected by possible outliers. If outliers/noise occur in the training set, one can estimate the pdf using robust estimators (Huber and Ronchetti 2009). The drawback, however, is that estimating densities is not an easy problem. There are two approaches to estimate densities:

- **Parametric** The parametric approaches assume a parametric data distribution, such as Gaussian or Poisson distribution, and discordancy tests are used to test new instances (Hawkins 1980; Barnett and Lewis 1994). The disadvantage of this approach is that the parametric distribution is generally not known a priori, and assuming a particular distribution for the data may impose a very restrictive model, resulting in a large bias when the assumption is violated.
- **Non-parametric** Non-parametric models do not assume any distribution, resulting in very flexible models. The disadvantage, however, is that it requires a sufficiently large sample of inliers to produce a good estimation. Depending, e.g., on the dimensionality of the problem, the number of instances required to sufficiently represent the underlying distribution can become very large, possibly impractical to obtain in a real-world setting.

Despite the difficulties in estimating densities, the density methods often outperform the others when the assumptions are satisfied (see Sect. 6). Commonly used density methods for one-class classification are the Gaussian density (Bishop 2007) and Gaussian Mixture Model (Bishop 2007) (parametric), as well as Parzen density estimation (Parzen 1962) (non-parametric).

#### Boundary methods

Similarly to the non-parametric density methods, boundary methods do not make any assumption about the data distribution, but instead of using a large number of observations (e.g. a large sample) to estimate the class density, they address the problem in a different, possibly simpler way. The boundary methods define a boundary around the training data, such that new instances that fall within the boundary are classified as inliers, while instances falling outside the boundary are classified as outliers (Fig. 1d). Since we are only interested in defining this boundary, it is not necessary to obtain a large sample to fully represent the inlier class. Another advantage of defining this boundary is that the threshold to classify new instances does not need to be set explicitly. On the other hand, the decision boundary can be quite sensitive to outliers/noise that might occur in the training set. Another disadvantage is that boundary methods also tend to be sensitive to the scaling of the feature space coordinates.

Because of their simplicity and effectiveness, boundary methods are quite popular. However, these methods can be highly computationally demanding due to the optimization problem that they require to solve. Boundary methods include the well-known Support Vector Data Description (SVDD) (Tax and Duin 2004) and Linear Programming (LP) (Pekalska et al. 2002) algorithms.

#### Reconstruction methods

Reconstruction methods compute a compact representation of the training set while attempting to preserve most of the information of the data. There are usually two ways to compute this compact representation of the data. One way is by clustering the dataset to represent it by a few prototypes. The other way is by projecting the data to represent it by a few dimensions. In this representation of the data, the noise contribution is usually reduced when compared to the original data, which makes these methods naturally more robust to noise, in contrast to the boundary methods. Once the compact representation has been obtained, new instances can then be reconstructed in the original data space through this representation. The reconstruction error, the difference between the original instance and the reconstructed instance, indicates the resemblance of a new instance to the original training distribution. In case of a representation by prototypes, the reconstruction error is measured as the distance of the original instance to the closest prototype. In representations by projection, the reconstruction error is measured as the distance of the original instance to its reconstructed (mapped) version. The new instances are classified using a threshold in the reconstruction error. Outliers should be more difficult to reconstruct than true inliers, and their reconstruction error is expected to be high. Reconstruction methods include approaches such as auto-encoder networks (Tax 2001; Goodfellow et al. 2016; González and Dasgupta 2002).

#### Deep learning methods

In recent years, deep learning methods (LeCun et al. 2015; Goodfellow et al. 2016) have shown tremendous abilities in learning representations on large and complex data, such as image and text. These methods utilize model architectures with multiple processing layers to learn data representations with multiple levels of abstraction. Deep (multi-layered) neural networks are especially well-suited for this task (Fig. 1g). In one-class classification, we can categorize the deep learning approaches (Ruff et al. 2018, 2020; Pang et al. 2022) into mixed and fully deep (or end-to-end). In mixed approaches, the methods leverage deep learning only in part of the pipeline. Usually, off-the-shelf deep learning methods (Simonyan and Zisserman 2015; Krizhevsky et al. 2017) are applied for feature extraction in a preceding step, and these features are then fed into classical one-class classification methods, such as SVDD; or the other way around, after (classic) feature extraction, deep learning methods are applied to learn the representation of normality, for example, using deep autoencoders (Hinton and Salakhutdinov 2006; Vincent et al. 2010) or Generative Adversarial Networks (GAN) (Schlegl et al. 2017; Goodfellow et al. 2014; Liu et al. 2020). Fully deep approaches, on the other hand, employ deep learning in an end-to-end fashion (Ruff et al. 2018, 2020; Perera and Patel 2019).

### Unsupervised outlier detection

In unsupervised outlier detection, we are provided with a set of unlabeled observations and are tasked with determining whether an observation is an inlier or an outlier. In this scenario, an outlier may be defined as an observation that deviates from other observations in some significant way, as determined by the chosen method. Unsupervised outlier detection methods are generally categorized as global or local methods (Schubert et al. 2014). With global methods, a given statistic of interest is compared between each observation and the entire dataset. If the observation’s statistic is extreme in some sense (sufficiently low or high), it is considered an outlier. In contrast, local methods compare the statistic associated to an observation to that of its neighbors only, rather than the entire dataset. There is a variety of approaches for unsupervised outlier detection, including density-based, distance-based, cluster-based, angle-based, and subspace methods.

#### Density-based methods

Density-based methods assume that outliers will appear in a region of low density (Fig. 1b), often according to some non-parametric measure of density, while inliers will appear in dense regions (Fig. 1c). Note that differently from the one-class classification density methods, density-based methods for unsupervised outlier detection calculate the point-wise density of each observation, rather than a density function for the entire inlier class. Density-based methods include LOF (Breunig et al. 2000) and LOCI (Papadimitriou et al. 2003).

#### Distance-based methods

Distance-based techniques assume that outliers are observations that are far away from their nearest neighbors (Fig. 1e), while inliers are close to their nearest neighbors (Fig. 1f). There are different (dis)similarity measures that can be used to measure distance. While for numeric continuous attributes the Euclidean distance is the most usual [in which case these methods can also be seen as density-based (Zimek and Filzmoser 2018)], for categorical attributes the simple matching coefficient is one of the most common choices (Tan et al. 2005). A classic distance-based method is the kNN outlier algorithm (Ramaswamy et al. 2000).

#### Cluster-based methods

Cluster-based methods use data clustering in order to detect outliers. The intuition behind these methods is that observations that do not fit well into clusters can be considered outliers (Fig. 1j). An example is GLOSH, which is based on the HDBSCAN* clustering hierarchy (Campello et al. 2015).

#### Angle-based methods

Angle-based methods represent each observation of the dataset as a point in Euclidean space, which restricts these methods to numeric datasets. The intuition behind these methods is that by measuring the variance in the angles between an observation and other observations, we can determine whether or not an observation is an outlier. If the variance is high (Fig. 1l), this may suggest that the observation is surrounded by other observations (in a cluster), while a low variance (Fig. 1k) may suggest that the observation is far away from other observations (an outlier). Using the angle of the points as additional information, rather than a measure based on distance only, tends to make these methods more robust when applied to high-dimensional datasets. The seminal method of this category is ABOD (Kriegel et al. 2008).

#### Subspace methods

Since too many irrelevant (noise) dimensions in a dataset can easily mask outliers, subspace outlier detection methods use only the most relevant attributes that make the outliers easier to detect (see Fig. 1h, i) (Zimek et al. 2012). However, finding the right subspace where an outlier deviates from the remainder of the data is not trivial, particularly because searching for every possible subspace using brute-force is not feasible, and the right subspace may vary from outlier to outlier. An example of a subspace method is SOD (Kriegel et al. 2009).

### Adapting unsupervised outlier detection methods to the one-class classification setting

**Fig. 2:**
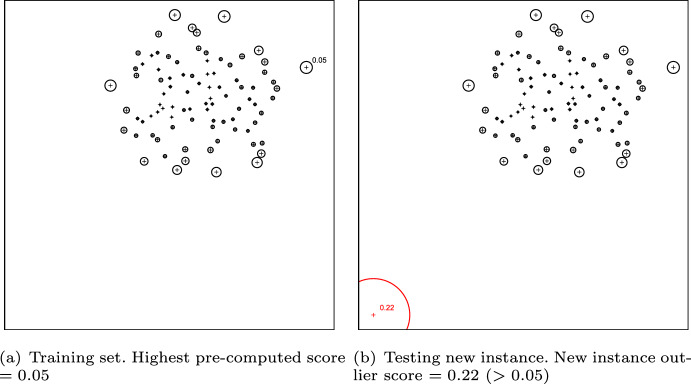
Illustrative example of the framework for adapting unsupervised outlier detection methods to one-class classification. The training instances are displayed as black crosses. The new instance to be classified is displayed as a red cross. The size of the circle around each cross reflects its outlier score: the larger the circle, the higher the outlier score (Color figure online)

A commonality among unsupervised outlier detection methods is that they compute a certain outlier score for each instance. To adapt an unsupervised outlier detection method to one-class classification, the general strategy is as follows: First run the unsupervised method on the (one-class) training data, pre-computing the scores for each inlier (Fig. 2a). Then compute the score for a new instance to be classified (Fig. 2b), possibly using other pre-computed quantities (e.g., densities or distances to nearest neighbors) related to instances in the training data. Then, in order to classify the new instance, compare its score with the pre-computed scores for the inliers. In the case of the example in Fig. 2, the new instance is classified as outlier, as its score is higher than any pre-computed scores for the inliers. In order to make the adapted algorithms more robust against noise or possible outliers in the training set, it is possible to exclude a top-scoring fraction $\alpha$ of the training set, which in practice is equivalent to establishing a score threshold. In this case, a new instance will be considered an outlier if it has a score higher than the scores in the training set, excluding the top $\alpha$ scores.

There are two important aspects related to the use of pre-computed quantities that involve training data when computing the outlier score for a new observation to be classified. First, when classifying multiple observations, there is no need to recompute these quantities over and over again for each new observation, since they relate solely to the training data (inlier class model) and can thus be pre-computed, which makes computations faster. Second and more importantly, using pre-computed quantities regarding the observations in the training data assures that the model is by no means affected by new observations to be classified. This is a basic principle of one-class classification, i.e., unlabeled observations should not affect the pre-computed inlier class model, since they may be outliers. For example, suppose that a certain algorithm operates by comparing the density of a new observation to be classified against the densities of its nearest neighbors among the known inliers (training data). In this case, the densities of the inliers should be pre-computed, not to be affected by the presence of the unlabeled observation being currently assessed; otherwise, each unlabeled observation would affect the model in a different way, which means that different observations would be classified by different models/criteria.

When classifying multiple observations, it is also recommended that the classification procedure described above be performed independently for each observation. This way, different unlabeled observations will not affect each other’s assessment. The reason we only classify one observation at a time instead of multiple observations at once is because we make no assumptions about the nature of each observation in relation to the combined dataset as a whole. It is possible that observations to be classified, while outliers in the sense that they do not belong to the inlier class, may be grouped together in such a way that an unsupervised method would not detect them as outliers.

## Model selection for one-class classification

Model selection methods for one-class classification rely on classification performance to guide the search for the best model (parameterization/algorithm). These methods search for the best model, such that some measure of classification performance is optimized. In most one-class classification problems, we are not only interested in models that have a small overall classification error, but also in what type of instances are misclassified. Due to the inherent imbalance of the outlier class, it is trivial to think of cases where a model can exhibit a small overall error, while it misclassifies a high proportion of outliers. In one-class classification problems, where misclassifying outliers as inliers is particularly critical (e.g., in fraud detection), we are also interested in the minimization of the error in the outlier class. Therefore, the error of a one-class classification model, in general, can be expressed as:

1 $$\begin{aligned} \Lambda (\epsilon _i, \epsilon _o) = \beta \epsilon _i + (1 - \beta )\epsilon _o \end{aligned}$$

where $\epsilon _i$ is the inlier rejection rate, i.e., the rate of inliers classified as outliers (false positives), and $\epsilon _o$ is the outlier acceptance rate, the rate of outliers classified as belonging to the inlier class (false negatives). The convex combination parameter $\beta$ controls the trade-off between the importance of outlier acceptance ($\epsilon _o$) and inlier rejection ($\epsilon _i$). For $\beta = 0.5$ both errors are equally treated. If estimates of $\epsilon _i$ and $\epsilon _o$ are available, we can estimate classification error for multiple candidate models and point out which one is better in relative terms. The problem, however, is that in one-class classification only instances of the inlier class are available during the training phase. Therefore, only $\epsilon _i$ can be directly estimated on the training set, which can be achieved by using traditional supervised techniques such as cross-validation or holdout (Han et al. 2011; Tan et al. 2005). The expected error on the outlier class ($\epsilon _o$), in turn, can only be estimated by making additional assumptions. In the next section, we present three methods capable of estimating the classification error and, therefore, performing model selection for one-class classification in the absence of training observations from the outlier class.

### Methods for model selection

#### Uniform object generation

**Fig. 3:**
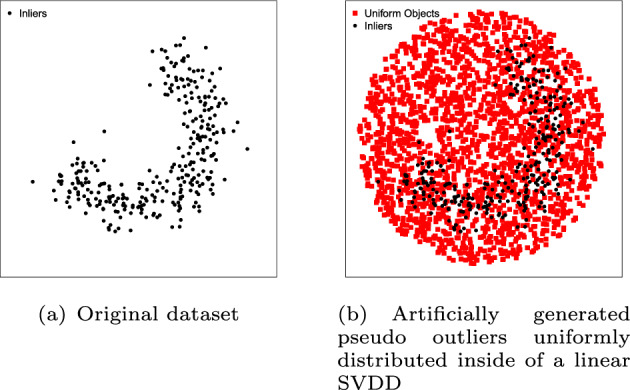
Illustrative example of the Uniform Object Generation method. The classifier is trained on the original dataset (**a**) and the rate of pseudo outliers classified as inliers is the estimate of $\epsilon _o$. $\epsilon _i$ is estimated by cross-validation using only the original dataset (**b**)

Uniform object generation (Tax and Duin 2001b) assumes that the outlier class is uniformly distributed in the input space. The method fits the smallest enclosing hypersphere around the training set, computed by a linear SVDD algorithm (Tax and Duin 2004), then it generates a set *Q* of uniformly distributed outliers inside of it (Fig. 3b). The rate of generated outliers that are classified as inliers by a given one-class classifier is the estimate of the error of that classifier on the outlier class ($\epsilon _o$). The idea behind this approach is that when the volume covered by the classification model under evaluation is minimized, the chances that it will accept outliers is also minimized. As pointed out by the authors themselves (Tax and Duin 2001b), a drawback of this method is that in high dimensional feature spaces the procedure becomes computationally inefficient. The reason is that it requires an exponentially large number of artificially generated outliers to minimally cover the space so as to get a confident estimate of the volume of the classifier. For instance, given 100,000 uniformly distributed artificial data objects (observations), this method has been deemed accurate only up to 15–20 dimensions, depending on the data distribution (Juszczak 2006).

In order to estimate the error on the inlier class ($\epsilon _i$), the authors propose to use cross-validation, since the labels for inlier class data objects are available.

#### Self-adaptive data shifting (SDS)

**Fig. 4:**
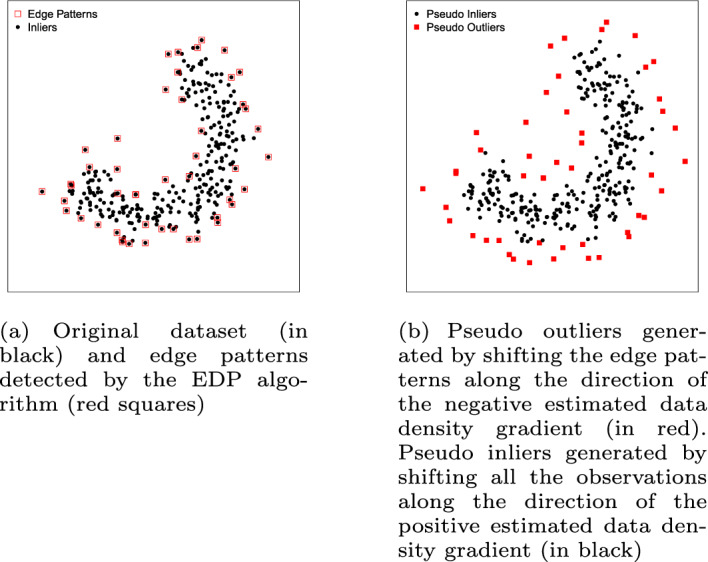
Illustrative example of the SDS method. The classifier is trained on the original dataset (**a**). The error terms $\epsilon _i$ and $\epsilon _o$ are estimated on the artificially generated data (**b**)

SDS (Wang et al. 2018) relies on an Edge Pattern Detection (EPD) algorithm to find the edges of the data, i.e., the instances in the border of the class. After the EDP algorithm finds the edge patterns (Fig. 4a), for a given neighborhood and a threshold value to distinguish the edge patterns from other observations, the corresponding instances are used to generate pseudo outliers. The pseudo outliers are generated by shifting these instances along the direction of the negative estimated data density gradient (Fig. 4b). In addition to the pseudo outliers, the SDS method also generates pseudo inliers by shifting each instance of the dataset along the positive direction of the data density gradient (Fig. 4b). After generating pseudo instances for both classes, the one-class classification model uses the original data for training and the generated data as the testing set to estimate $\epsilon _i$ and $\epsilon _o$. Note that in contrast to Uniform Object Generation, SDS does not use the true inliers to estimate $\epsilon _i$, therefore, the synthetic dataset generated by SDS (Fig. 4b) does not contain true inliers. The main weakness of this method is the performance of the EDP algorithm in high dimensional spaces. High dimensional datasets tend to become sparse, and the EDP algorithm begins to see every instance as an edge pattern.

#### Data perturbation

**Fig. 5:**
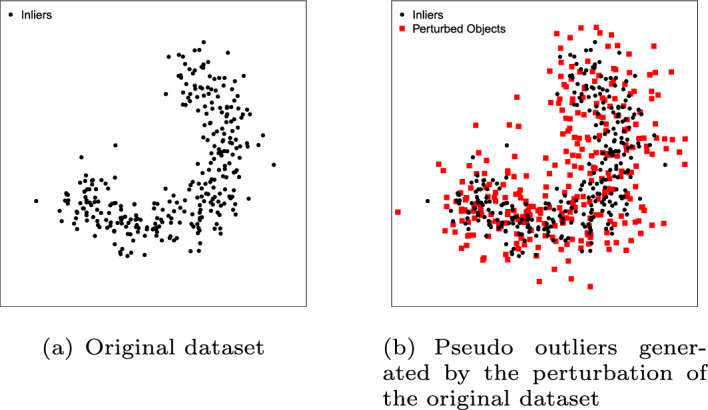
Illustrative example of the Data Perturbation method. The classifier is trained on the original dataset (**a**) and the rate of pseudo outliers classified as inliers is the estimate of $\epsilon _o$. $\epsilon _i$ is estimated by cross-validation using only the original dataset (**b**)

Data Perturbation (Marques 2019) adds a small, randomized amount of noise to each attribute value of all observations. The underlying premise of this method is that when a perturbation is applied to the data, the borderline observations can be affected enough to become outliers. The tighter the fit of the model to the data, the higher the number of observations close to the decision boundary. Therefore, the models with a tighter fit should result in a larger number of observations that become outliers after the perturbation. This perturbation is implemented by adding to each instance an attribute-wise noise component independently sampled from ${\mathcal {N}}(0, \sigma )$, where $\sigma$ corresponds to the value whereby a fraction of 50% of the data is rejected by a linear SVDD. Similarly to Uniform Object Generation, the error on the inlier class ($\epsilon _i$) is estimated using cross-validation and the error on the outlier class ($\epsilon _o$) is estimated as the rate of generated outliers (perturbed observations, Fig. 5b) classified as inliers. In order to make the results more robust, the process of data perturbation can be applied multiple times to generate *m* different instances of the dataset. According to the experiments reported in Marques 2019, this method performed consistently well on synthetic datasets, but not so well in real-world scenarios involving a multi-modal inlier class with clusters of very different sizes/dispersion, or in scenarios involving small sample sizes.

### Time complexity for model selection

The computational complexity of the one-class classification algorithms can be analyzed in terms of the two different sub-tasks involved, namely, the training time ${\mathcal {O}}(f_{ tr }(N))$ and the testing (or consultation) time ${\mathcal {O}}(f_{ te }(M))$, as a function of the training set size (*N*) and the test set size (*M*), respectively. Once a classifier is trained, we can divide the process of error evaluation into three phases: the generation of data objects that we use to estimate $\epsilon _i$ and/or $\epsilon _o$, the estimation of $\epsilon _i$ and the estimation of $\epsilon _o$. The phase of object generation for Data Perturbation and Uniform Object Generation involves the training of a *linear* SVDD, which can be efficiently computed in linear time (${\mathcal {O}}(N)$) (Joachims 2006; Erfani et al. 2015).^1^ SDS, in turn, needs to compute the kNNs for each object, which has a worst case complexity of ${\mathcal {O}}(N^2)$, although it can often be computed more efficiently with the use of suitable indexing structures.

The estimation of $\epsilon _i$ by Data Perturbation and Uniform Object Generation, both of which use *k*-fold cross-validation on the true inliers, takes ${\mathcal {O}}\Big (k\cdot \Big (f_{ tr }\Big (\frac{(k-1)\cdot N}{k}\Big ) + f_{ te } \Big (\frac{N}{k}\Big )\Big )\Big )$. SDS uses the artificially generated data to estimate both $\epsilon _i$ and $\epsilon _o$ in ${\mathcal {O}}(f_{ te }(N))$ time. Notice that the runtime for estimation of $\epsilon _o$ in all methods depends on the testing time over the number of objects generated, since the classifier has to test each of these objects. However, each method generates a different amount of objects: Data Perturbation generates an amount proportional to (and not smaller than) the size of the dataset (*N*); SDS generates an amount less or equal to *N*; Uniform Objects generates a user-defined number of objects (*Q*) that does not depend on the size of the dataset, but the number that is required for good performance grows fast with increasing dimensionality (Désir et al. 2013; Tax and Duin 2001b; Juszczak 2006). In Table 1, we summarize the complexity of the methods.

**Table 1:** Time complexity for model selection

	Gen	$\epsilon _i$	$\epsilon _o$
Uniform objects	${\mathcal {O}}(N)$	${\mathcal {O}}\left( k\cdot \left( f_{ tr }\left( \frac{(k-1)\cdot N}{k}\right) +f_{ te }\left( \frac{N}{k}\right) \right) \right)$	${\mathcal {O}}(f_{ te }(Q))$
SDS	${\mathcal {O}}(N^2)$	${\mathcal {O}}(f_{ te }(N))$	${\mathcal {O}}(f_{ te }(N))$
Perturbation	${\mathcal {O}}(N)$	${\mathcal {O}}\left( k\cdot \left( f_{ tr }\left( \frac{(k-1)\cdot N}{k}\right) +f_{ te }\left( \frac{N}{k}\right) \right) \right)$	${\mathcal {O}}(f_{ te }(Nm))$

*N*: training set size, *k*: number of folds in cross-validation, *Q*: number of generated uniform objects (user-defined parameter), *m*: number of perturbed instances of the dataset (user-defined parameter)

## Experimental setup

### Algorithms

We compare and evaluate 7 one-class classification algorithms, namely, Gaussian Mixture Model (GMM), Parzen Window (PW), Support Vector Data Description (SVDD), Linear Programming (LP), *k*-Nearest Neighbor Data Description (which we call here $\hbox {kNN}_{\textit{local}}$), Auto-Encoder, and Deep SVDD (DSVDD) against 7 unsupervised outlier detection algorithms adapted to one-class classification, namely, *k*-Nearest Neighbors (hereafter referred to as $\hbox {kNN}_{\textit{global}}$), Local Outlier Factor (LOF), Local Correlation Integral (LOCI), Global–Local Outlier Scores from Hierarchies (GLOSH), Isolation Forest (iForest), Angle-Based Outlier Detection (ABOD), and Subspace Outlier Degree (SOD). The full description of all compared methods, including their asymptotic computational complexities, is given in Section 1 in the Supplementary Material.

The parameters of the methods were varied in the following ranges: $k = 2, 4, 6, \ldots , 50$ for LOF, $\hbox {KNN}_{\textit{global}}$, $\hbox {KNN}_{\textit{local}}$, and SOD; $M_{\textit{clSize}} = M_{\textit{pts}} = 2, 4, 6, \ldots , 50$ for GLOSH; *l* = *k* and the coefficient for the expected variance along the attributes = 0.8 for SOD (as suggested by the authors); number of hidden units = $2, 4, 6, \ldots , 50$ for Deep SVDD and for the Auto-Encoder with scaled conjugate gradient backpropagation training algorithm (Møller 1993); number of Gaussians = $1, 2, 3, \ldots , 25$ for GMM; *h* varying from the largest distance in the dataset to the smallest nearest neighbor distance (into 25 equally spaced values) for PW, LP, and SVDD, as common practice in the literature (Liu et al. 2012; Tax and Müller 2004); fraction of the dataset $\varphi = 0.1, 0.25, 0.5, 0.75, 1$ for LOCI and iForest; number of iTrees = 10, 25, 50, 75, 100 for iForest. Also, all the methods were allowed to misclassify a fraction of the training set $\alpha = 0, 0.05, 0.1, 0.15$ during the model training, as discussed in Sect. 3.3. The ranges of parameters used for each method are summarized in Table 2.

**Table 2:** Ranges in which the parameters of the methods were varied

Method	Parameters		
	$\alpha$	$t_1$	$t_2$
ABOD	$\{0, 0.05, 0.1, 0.15\}$		
LOF	$\{0, 0.05, 0.1, 0.15\}$	$k = \{2, 4, 6, \ldots , 50\}$	
$\hbox {KNN}_{\textit{global}}$	$\{0, 0.05, 0.1, 0.15\}$	$k = \{2, 4, 6, \ldots , 50\}$	
$\hbox {KNN}_{\textit{local}}$	$\{0, 0.05, 0.1, 0.15\}$	$k = \{2, 4, 6, \ldots , 50\}$	
SOD	$\{0, 0.05, 0.1, 0.15\}$	$k = l = \{2, 4, 6, \ldots , 50\}$	
GLOSH	$\{0, 0.05, 0.1, 0.15\}$	$M_{\textit{clSize}} = M_{\textit{pts}} = \{2, 4, 6, \ldots , 50\}$	
Auto-Encoder	$\{0, 0.05, 0.1, 0.15\}$	Hidden units = $\{2, 4, 6, \ldots , 50\}$	
DSVDD	$\{0, 0.05, 0.1, 0.15\}$	Hidden units = $\{2, 4, 6, \ldots , 50\}$	
GMM	$\{0, 0.05, 0.1, 0.15\}$	Gaussians = $\{1, 2, 3, \ldots , 25\}$	
PW	$\{0, 0.05, 0.1, 0.15\}$	$h = \text {min-max}(\Vert {\textbf{x}}_i - {\textbf{x}}_j\Vert ^2)$	
LP	$\{0, 0.05, 0.1, 0.15\}$	$h = \text {min-max}(\Vert {\textbf{x}}_i - {\textbf{x}}_j\Vert ^2)$	
SVDD	$\{0, 0.05, 0.1, 0.15\}$	$h = \text {min-max}(\Vert {\textbf{x}}_i - {\textbf{x}}_j\Vert ^2)$	
LOCI	$\{0, 0.05, 0.1, 0.15\}$	Frac. data $= \{0.1, 0.25, 0.5, 0.75, 1\}$	
iForest	$\{0, 0.05, 0.1, 0.15\}$	Frac. data $= \{0.1, 0.25, 0.5, 0.75, 1\}$	iTrees = $\{10, 25, 50, 75, 100\}$

$\alpha$ is the percentage of the dataset that can be misclassified during the training, while $t_1$ and $t_2$ refer generically to additional user-defined parameters of each method. Most methods have only one parameter to configure in addition to $\alpha$, ABOD has none, and iForest has two

### Datasets

We use different collections of previously published, real and synthetic benchmarking datasets to evaluate the results of our experiments. For experiments on synthetic data, we adopt a collection of benchmarking datasets from the literature (Zimek et al. 2013b, 2014) to evaluate the methods’ performance to detect local outliers. This collection has been designed and used to evaluate outlier detection methods. Specifically, we use the set of 30 datasets referred to as “*batch1*” (Zimek et al. 2013b, 2014), which vary in dimensionality *d* ($d \in [20, \dots , 40]$), in the number of classes *c* ($c \in [2, \dots , 10]$), and for each class $c_i$ independently in the number of points $n_{c_i}$ ($n_{c_i} \in [600, \dots , 1000]$). For each class, the points are generated following a Gaussian model with randomly selected parameters that are attribute-wise independent. The points that have a Mahalanobis distance to their class center larger than the *theoretical* 0.975 quantile of the $\chi ^2_d$ distribution were labeled as (local) outliers.

For experiments on real data, we use 31 real-world datasets from the UCI Machine Learning Repository (Dheeru and Karra Taniskidou 2017) as pre-processed for one-class classification and made available by Tax (2001), namely: Abalone, Arrhythmia, Balance-scale, Ball-bearing, Biomed, Breast, Cancer, Colon, Delft1x3, Delft2x2, Delft3x2, Delft5x1, Delft5x3, Diabetes, Ecoli, Glass, Heart, Hepatitis, Housing, Imports, Ionosphere, Iris, Liver, Satellite, Sonar, Spectf, Survival, Vehicle, Vowels, Waveform, and Wine.

In addition, we also include the datasets CellCycle-237 and YeastGalactose, made public by Yeung et al. (2001, 2003)), and 20 other real-world multi-class datasets from the UCI Machine Learning Repository (Dheeru and Karra Taniskidou 2017), namely: Artificial Characters, Cardiotocography, Car Evaluation, CNAE-9, Dermatology, Solar Flare, Hayes-Roth, LED Display, Lung Cancer, Multiple Features, Optical Recognition, Page Blocks, Seeds, Semeion, Soybean, Synthetic Control, Texture, User Knowledge Modeling, Vertebra Column, and Zoo. Properties of these datasets are presented in Tables 2 and 3 in the Supplementary Material, where we describe the number of features, the number of objects in each class, and the total number of objects in the datasets.

We report only a single, aggregated result (the average) for the 5 Delft datasets, and we also report only the average result for the 30 synthetic datasets, since both sets contain variants obtained from a single source. This way, in total we report results corresponding to 50 independent dataset sources. Finally, due to the inability of some algorithms to deal with replicated observations, duplicates are removed from the datasets where they are present.

All the source codes and datasets used in the experiments are publicly available in a repository at https://github.com/homarques/occ.

### Experimental scenarios

In order to consider fundamentally distinct one-class classification scenarios, we perform different types of experiments. Regarding the labeling of datasets, i.e., the labeling of observations into inliers and outliers, two major types of experiments were carried out. In the **first type** of experiment (Type I), we follow the only approach taken by Janssens et al. (2009), where multi-class datasets are transformed into one-class datasets by re-labeling one of the classes as inliers, while the remaining classes are labeled as outliers. Except for datasets where only a single inlier class has been pre-defined as such in the data repository (Tax 2001)—e.g., Ecoli—we repeat the procedure as many times as there are classes in the dataset, each time labeling a different class as inliers. Among the resulting binary (e.g. inlier/outlier) datasets, those for which none of the methods were able to reach a classification performance of at least 0.5 as measured by ROC AUC were discarded (e.g., the Arrhythmia dataset with class 2 as inliers). By the above procedure, the initial 50 multi-class independent datasets yield 200 Type I one-class classification datasets.

**Fig. 6:**
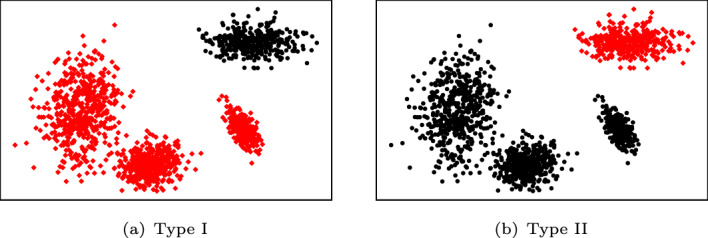
Two major types of experiments w.r.t. the labeling of datasets. Inliers marked in black dots and outliers marked in red diamonds (Color figure online)

For the **second type** of experiment (Type II), we reverse the inlier and outlier classes obtained in the first type of experiment for datasets that have more than 2 possible inlier classes. This type of experiment models the important situations where the inlier class is more likely multi-modal as it combines 2 or more classes of the original dataset. Note that Type II experiments are only different from Type I (and, as such, they are only reported) for datasets with 3 or more classes. Accordingly, results for Type II experiments are only available for a subset of the datasets used in Type I experiments. More precisely, the initial 50 multi-class independent datasets yield 157 Type II one-class classification datasets. Figure 6 shows an example of Type I and II experiments w.r.t. the labeling of a given multi-class dataset.

In addition to Type I and Type II experiments, we also evaluate the behavior of the methods in other scenarios. In the **third type** of experiments (Type III), we use the collection of 30 synthetic datasets to evaluate the methods’ performance to detect the local outliers as originally labeled in those datasets (e.g., observations in the vicinity/tails of normally distributed classes—see Sect. 5.2).

**Fig. 7:**
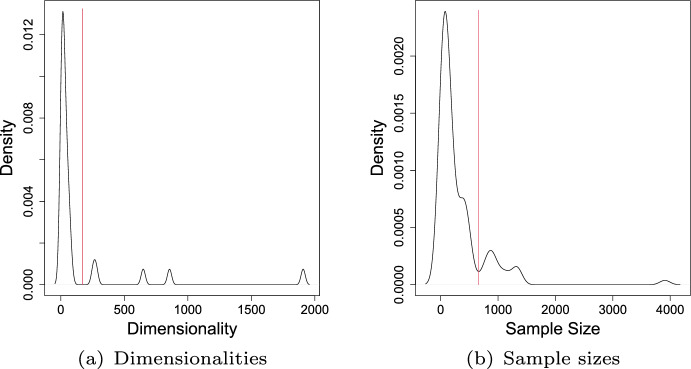
Distribution of datasets with respect to dimensionality (**a**) and sample size (**b**). Red vertical bars indicate adopted thresholds (Color figure online)

In the **fourth type** of experiments (Type IV), we use datasets with high dimensionality to assess the performance of the methods in high dimensional spaces. Finally, we use datasets with larger sample sizes in the **fifth type** of experiments (Type V) to evaluate the performance of the methods when information for training becomes sufficiently large. We define “high dimensionality” and “larger sample size” based on the distribution of dimensionalities and sample sizes of the datasets, as shown in Fig. 7. The 31 one-class classification datasets with dimensionality higher than 200 were considered high dimensional datasets, and the 13 one-class classification datasets with a sample size of more than 600 were considered to have larger sample sizes. These thresholds are indicated by the vertical red lines in Fig. 7. For Types IV and V experiments, we consider only the scenario of single source-class inliers (Type I experiments). Therefore, we can see these experiments as a subset of the first type of experiments. We adopted the separation of these two additional scenarios, as it is known that there are methods designed specifically for high dimensionality [e.g., ABOD (Kriegel et al. 2008)], and others that require a sufficiently large sample for good performance [e.g., Parzen Window (Parzen 1962)]. Therefore, we can evaluate these methods where they are supposed to perform best.

### Evaluation measures

For the evaluation of the experiments, we use three well-known classification measures (Baldi et al. 2000; Désir et al. 2013; Wang et al. 2018; Campos et al. 2016): Area Under the ROC Curve (ROC AUC), Adjusted Precision-at-*n* (AdjustedPrec@n), and Matthews Correlation Coefficient (MCC).

#### MCC

Matthews Correlation Coefficient (MCC) (Matthews 1975) is a discrete version of Pearson’s correlation coefficient (Kirch 2008) between the ground truth and the predicted labels. MCC ranges from $-1$ (all predictions are wrong) to $+1$ (perfect classification). A value of 0 indicates that either predictions are completely random or the classifier always predicts only one of the classes. MCC can also be calculated from a contingency table as:

2 $$\begin{aligned} {\text {MCC}} = \frac{{\text {TP}} \times {\text {TN}} - {\text {FP}} \times {\text {FN}}}{\sqrt{({\text {TP}}+{\text {FP}})({\text {TP}}+{\text {FN}})({\text {TN}}+{\text {FP}})({\text {TN}}+{\text {FN}})^{}}}, \end{aligned}$$

where TP (True Positives) is the number of true outliers correctly classified, TN (True Negatives) is the number of true inliers correctly classified, FN (False Negatives) is the number of true outliers that have been misclassified and FP (False Positives) is the number of true inliers that have been misclassified.

Unlike other classification measures that are also suitable for class imbalance problems (e.g. recall, precision, and $F_1$ score), MCC does not require to define which class is underrepresented. This is convenient in our experimental setup because in Type I experiments (Fig. 6a) the inlier class tends to have fewer instances than the outlier class, whereas in Type II experiments (Fig. 6b) it is the other way around.

#### ROC AUC

In one-class classification and unsupervised outlier detection, the output of the algorithms is usually a real-valued score, and the quantities TP, TN, FP and FN depend on how a threshold is selected. Generally, there is a trade-off between the amount of false positives and the amount of false negatives produced by an algorithm. The Receiver Operating Characteristic (ROC) curve shows, for all possible threshold choices, the true positive rate ${\text {TPR}} = \frac{{\text {TP}}}{{\text {TP}}+{\text {FN}}}$ versus the false positive rate ${\text {FPR}} = \frac{{\text {FP}}}{{\text {FP}}+{\text {TN}}}$. As TPR and FPR normalize the number of true and false positives, respectively, ROC inherently adjusts for the imbalance of class sizes.

The performance of a classifier as represented by a ROC curve can be summarized by a single value by computing the area under this curve (ROC AUC). The ROC AUC value ranges between 0 and 1. A perfect ranking of the instances (in which all outliers are ranked ahead of any inliers) results in a ROC AUC value of 1, whereas an inverted perfect ranking produces a value of 0. The expected value for a random ranking of the instances is a ROC AUC value of 0.5.

#### AdjustedPrec@n

As stated above, most one-class classification and unsupervised outlier detection algorithms output real-valued scores. Precision-at-*n* (prec@n) measures the fraction of true outliers among the top *n* outlier scores in a test set:

3 $$\begin{aligned} {\text {prec@n}} = \frac{|\{o \in O \vert rank(o) \le n\}|}{n}, \end{aligned}$$

where *O* is the set of true outliers and *n* is typically defined as the cardinality of this set (i.e., $n = |O |$, case in which ${\text {prec@n}} \in [0,1]$). To compare prec@n values across datasets with different numbers of outliers, one has to adjust prec@n by chance, as discussed in Campos et al. (2016). This gives rise to the AjustedPrec@n measure used in our experiments, in which the perfect ranking produces a value of 1, while a random ranking has an expected value of 0. The AjustedPrec@n in a test set can be computed as follows:

4 $$\begin{aligned} {\text {AjustedPrec@n}} = \frac{{\text {prec@n}} - |O |/M}{1 - |O |/M}, \end{aligned}$$

where *M* is size of the test set and $|O |$ is number of true outliers in the test set.

#### ROC AUC versus MCC versus AdjustedPrec@n

**Fig. 8:**
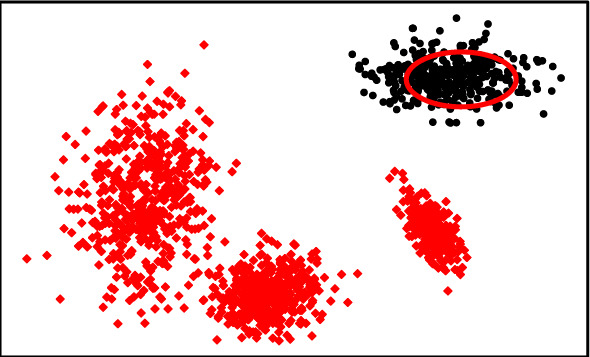
Illustrative example of a boundary descriptor (red ellipse). Inliers are displayed as black dots while outliers are highlighted as red diamonds. The boundary descriptor achieves a high ROC AUC, albeit a low MCC (Color figure online)

ROC AUC takes the entire ranking of real-valued outlier scores into account, without the need to select a particular threshold to label the observations as outliers or inliers. A high ROC AUC score indicates that, in the overall ranking, outliers are more likely to be ranked ahead of inliers. For the boundary methods, for example, it only means that the inliers are closer to the described boundary than the outliers; it does not necessarily mean that the boundary descriptor is accurately classifying the observations. Figure 8 shows an example of a boundary descriptor that achieves ROC AUC = 1, *i.e.,* a perfect ranking of the outlier scorings. However, the boundary descriptor of the classifier imposes a suboptimal threshold on the ranking, resulting in a large fraction of inliers misclassified. MCC, on the other hand, evaluates the classifier performance based on a binary outlier/inlier labeling, which is automatically provided by some one-class classification models,^2^ or, alternatively, it can be obtained by applying an arbitrary user-defined threshold to the produced scores. In the latter case, incorrectly defining such a threshold may lead to poor evaluations. For AdjustedPrec@n, setting the cardinality of the top *n* scores as the number of true outliers in the test data (e.g. $n = |O |$) can be seen as an implicit choice of threshold, which is sensible when ground truth information is available; however, in real-world one-class classification tasks the number of outliers is usually unknown. Therefore, we argue that one cannot rely solely on ROC AUC, MCC, or AdjustedPrec@n for quality assessment of models; rather, ROC AUC, MCC, and AdjustedPrec@n complement each other by revealing different aspects of the quality of a solution, which are all relevant in practice.

#### Friedman test

The Friedman test (Friedman 1937) is a non-parametric statistical test that ranks the algorithms based on their relative performance on each dataset, from best to worst, and checks for statistically significant differences between the ranks. The null hypothesis states that the average ranks of the compared algorithms are not significantly different. The Friedman statistic is given by:

5 $$\begin{aligned} \chi ^2_{(v-1)} = \frac{12n_d}{v(v+1)}\left( \sum ^v_{j=1} R^2_j - \frac{v(v+1)^2}{4}\right) \end{aligned}$$

which can be shown to follow a $\chi ^2$ distribution with $v - 1$ degrees of freedom when $n_d$ and *v* are big enough (as a rule of thumb, $n_d > 10$ and $v > 5$), where *v* is the number of classifiers (algorithms being compared) and $n_d$ is the number of datasets (sample size for the test). $R_j$ is the average rank of classifier *j* taken over all datasets. For small values of $n_d$ or *v*, the approximation by the chi-square distribution becomes poor and the *p*-value should be obtained from tables specially prepared for the Friedman test (Demsar 2006). If the null-hypothesis is rejected, i.e., the *p*-value is significant, one can proceed with a post-hoc test.

#### Neményi test

The post-hoc Neményi test (Neményi 1963) is a pairwise test used to check whether there is a significant difference in performances between two algorithms. The performance of two algorithms is significantly different when the difference between their average ranks is greater than or equal to the critical difference CD:

6 $$\begin{aligned} {\text {CD}} = q_\alpha \sqrt{\frac{v(v+1)}{6n_d}} \end{aligned}$$

where $q_\alpha$ is the tabulated critical value for the test at the specified significance level (Neményi 1963). Note that the critical difference can be reduced by increasing the number of datasets $n_d$ or by decreasing the number of algorithms *v*. In the data mining and machine learning literature, results of the Friedman–Neményi tests are often visually represented by means of critical difference diagrams (Demsar 2006), which will be used later in this paper to summarize our results.

### Model selection

In order to evaluate a method’s performance on a one-class classification dataset, we first split the dataset (which, for the sake of external evaluation, contains both inlier and outlier ground truth labels) into 2 *stratified* subsets, such that the distribution of outliers and inliers remains as close as possible to the entire dataset: one subset containing 20% of the observations (test set) and the other containing the remaining 80% of the data (training set). Then, using the training subset with 80% of the data, we apply four different model selection procedures in order to select the best model (parameters/algorithms) from the collection of candidates under evaluation.

The first model selection procedure, which is the most commonly used approach when evaluating one-class classifiers in the literature, initially uses 10-fold cross-validation to select the algorithm’s parameter(s) that result in the best ROC AUC value on the training dataset.^3^ Since this procedure uses both inliers and outliers (labeled as such) in the training dataset, the latter of which are usually not available in real-world applications of one-class classification problems, this approach represents an optimistic evaluation based on an ideal scenario where the optimal algorithm parameter(s) is supposed to be determined in a traditional, fully supervised way. Because by definition this is not a realistic supposition when it comes to semi-supervised (one-class) learning applications, we also consider three additional model selection procedures. For these additional procedures, we select the best model *without* using any outliers from the training set.

The three approaches to select models in the absence of examples for the outlier class are Perturbation (Marques 2019), Uniform Objects (Tax and Duin 2001b), and SDS (Wang et al. 2018), which have been described in Sect. 4. For Perturbation, we generate *m* = 10 “perturbed” datasets in order to estimate $\epsilon _o$. For Uniform Objects, we estimate $\epsilon _o$ using $Q = 100{,}000$ uniformly distributed artificial data objects [the same amount used by the authors (Tax and Duin 2001b)]. For SDS, the neighborhood size and the threshold value used by the EDP algorithm were fixed as $\lceil 5\, \text {log}_{10} \, N \rceil$ and 0.1, respectively, as suggested by the authors (Wang et al. 2018). We use 10-fold cross-validation (excluding outliers) to estimate $\epsilon _i$ for Uniform Objects and Perturbation (Tax and Duin 2001b; Marques 2019).

After parameter selection, the subset containing 20% of the data (test set) is used to measure the performance of the resulting algorithms, i.e., the different candidate models as configured with parameter value(s) selected by the four procedures described above and, then, trained using all and only the inliers from the training set.

In order to get more reliable results, the whole procedure is repeated 30 times, and the resulting ROC AUC values are aggregated and reported. In addition to ROC AUC values, we also report the MCC and AdjustedPrec@n on the test sets (described in Sect. 5.4).

### Ensemble

In order to investigate how the results from different one-class classifiers as combined into an ensemble can compare against the use of single classifiers, we adopt a rank-based combination strategy. The main advantage of using a rank-based combination strategy as opposed to a score-based combination strategy is that there is no need to normalize the outlier scorings coming from the different classifiers. Score normalization is a complex problem (Kriegel et al. 2011; Platt 2000; Gao and Tan 2006), particularly when the goal is to make scores from different algorithms comparable in a statistically meaningful way by taking not only their scales into account but also their distributions. Here we circumvent this problem by using Reciprocal Rank Fusion (RRF) (Cormack et al. 2009), which is a ranking combination method based on the intuition that higher ranks are more important for the overall ranking, whereas the importance of lower ranked observations should vanish as it would if an exponential function was used.^4^ The results reported in this study can be seen as a lower bound in terms of the achievable performance of potential ensembles. The resulting, rank-based combined “outlier score” of an observation according to the RRF method is given by:

7 $$\begin{aligned} {\text {RRF score}}(s) = \sum _{\tau \in {\mathcal {R}}} \frac{1}{\epsilon + \tau (s)}, \end{aligned}$$

where ${\mathcal {R}}$ is the set of rankings $\tau$ produced by the different classifiers (ensemble members), $\tau (s)$ is the rank of observation *s* from a given classifier, and $\epsilon$ is a user-defined parameter. We use $\epsilon = 60$ as suggested by Cormack et al. (2009), which can mitigate the impact of abnormal classifier rankings.

## Results

### Comparison of algorithms under optimistic parameter tuning

In the following, we describe the comparison results of the different one-class classification algorithms when their parameters are set by using the first model selection approach described in Sect. 5.5 (namely, supervised cross-validation), in which not only inliers, but also outliers, are supposed to be available in the training data. As previously discussed, this represents an “optimistic” setup, which is typically not doable in practical applications; yet, it has been the standard setup adopted so far in the literature on one-class classification. The overall ranking results are summarized by critical difference diagrams (Demsar 2006) presented throughout the remainder of this section. Detailed results for all experiments are provided in Section 2 in the Supplementary Material.

**Table 3:** Average ROC AUC for the different types of experiments using cross-validation (optimistic parameter tuning)

ROC AUC	ABOD	Auto Enc	GLOSH	GMM	iForest	KNN G	SVDD	KNN L	LOCI	LOF	LP	Parzen	SOD	DSVDD
Single source-class inliers	0.8	0.85	0.83	**0**.**87**	0.83	0.82	0.85	0.81	0.83	0.84	0.83	0.81	0.83	0.82
Multiple source-class inliers	0.72	0.8	0.81	**0.83**	0.75	0.8	0.82	0.78	0.79	**0**.**83**	0.82	0.78	0.8	0.8
Local outliers	0.73	0.92	0.95	**0**.**99**	0.90	0.91	0.94	0.87	0.90	0.95	0.93	0.88	0.82	0.72
High-dimensional datasets	0.88	0.88	0.90	0.93	0.80	0.90	0.91	0.88	0.90	0.91	0.85	0.65	**0**.**94**	0.79
Larger sample size	0.80	0.88	0.84	**0**.**89**	**0**.**89**	0.84	0.88	0.84	0.87	0.86	0.86	0.84	0.83	0.81

The highest achieved values for each t﻿ype of experiment are shown in bold

**Table 4:** Average AdjustedPrec@*n* for the different types of experiments using cross-validation (optimistic parameter tuning)

AdjustedPrec@*n*	ABOD	Auto Enc	GLOSH	GMM	iForest	KNN G	SVDD	KNN L	LOCI	LOF	LP	Parzen	SOD	DSVDD
Single source-class inliers	0.45	0.57	0.52	** 0.59**	0.51	0.52	0.56	0.47	0.51	0.52	0.53	0.5	0.51	0.47
Multiple source-class inliers	0.32	0.49	0.5	0.52	0.39	0.5	** 0.54**	0.43	0.44	0.53	0.51	0.45	0.48	0.48
Local outliers	0.23	0.33	0.42	**0**.**65**	0.29	0.38	0.43	0.27	0.28	0.46	0.39	0.34	0.13	0.11
High-dimensional datasets	0.60	0.62	0.66	**0**.**75**	0.52	0.67	0.69	0.56	0.66	0.66	0.60	0.24	0.72	0.38
Larger sample size	0.49	0.60	0.54	0.61	**0**.**62**	0.55	0.59	0.50	0.58	0.56	0.56	0.52	0.51	0.49

The highest achieved values for each t﻿ype of experiment are shown in bold

**Table 5:** Average MCC for the different types of experiments using cross-validation (optimistic parameter tuning)

MCC	ABOD	Auto Enc	GLOSH	GMM	iForest	KNN G	SVDD	KNN L	LOCI	LOF	LP	Parzen	SOD	DSVDD
Single source-class inliers	0.41	0.36	**0**.**49**	0.37	0.47	0.47	0.32	0.45	0.46	**0**.**49**	0.28	0.37	**0**.**49**	0.38
Multiple source-class inliers	0.29	0.37	0.43	0.39	0.34	0.44	0.25	0.38	0.39	** 0.46**	0.26	0.32	0.43	0.40
Local outliers	0.18	0.34	0.40	**0**.**60**	0.29	0.33	0.23	0.27	0.28	0.42	0.28	0.29	0.17	0.12
High-dimensional datasets	0.48	0.41	0.57	0.17	0.50	0.57	0.28	0.45	0.51	0.61	0.07	0.22	**0**.**63**	0.3
Larger sample size	0.47	**0**.**62**	0.56	0.60	**0**.**62**	0.55	0.40	0.54	0.58	0.58	0.35	0.51	0.53	0.51

The highest achieved values for each t﻿ype of experiment are shown in bold

**Fig. 9:**
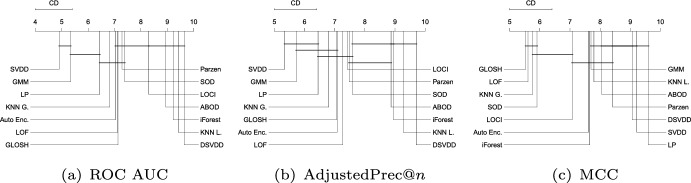
Critical difference diagrams with average ranks of the methods over all Type I experiments (single source-class inliers)

#### Type I: Single source-class inliers

Figure 9 shows the average ranks of the methods obtained by the Friedman test over all Type I experiments with respect to ROC AUC, AdjustedPrec@*n*, and MCC. When looking at ROC AUC and AdjustedPrec@*n*, one can see SVDD, GMM, and LP, in this order, at the top, among which statistical difference is observed (as indicated by the absence of critical difference bars) only between SVDD and LP for ROC AUC. The average ROC AUC for GMM, SVDD, and LP were 0.87, 0.85, and 0.83 (see Table 3), respectively, while the average AdjustedPrec@*n* values were 0.59, 0.56, and 0.53 (see Table 4). Notice that although SVDD has the best average rank, GMM has the best average ROC AUC/AdjustedPrec@*n* values due to poor performances of SVDD on certain individual datasets, such as e.g. Cardiotocography, where GMM obtains a ROC AUC value of 0.98 and an AdjustedPrec@*n* value of 0.91, whereas SVDD achieves only a ROC AUC value of 0.65 and an AdjustedPrec@*n* value of 0.14 (for the detailed results for each dataset, refer to Tables 4 and 8 in the Supplementary Material). This indicates that although SVDD was the best overall performer, there are particular application scenarios where even a highly effective algorithm such as SVDD may under-perform (no free lunch). When looking at MCC, the overall picture is very different: the boundary-based methods SVDD and LP, two of the top performers with respect to ROC AUC and AdjustedPrec@*n*, are the two worst performers with respect to MCC. This suggests that although the outlier rankings produced by these algorithms are good, the decision boundary leads to a misclassification of many observations, such as in the example shown in Fig. 8. For MCC, the neighbourhood-based outlier detection algorithms performed better, without statistical difference observed between GLOSH, LOF, $\hbox {KNN}_{\textit{global}}$, and SOD, in this order, at the top, with average MCC values of 0.49, 0.49, 0.47, and 0.49 (see Table 5), respectively.

**Fig. 10:**
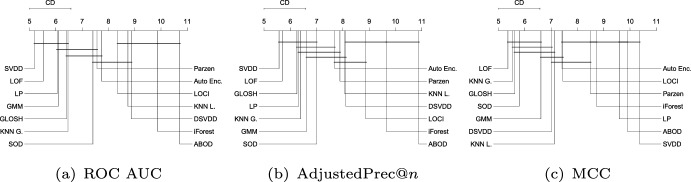
Critical difference diagrams with average ranks of the methods over all Type II experiments (multiple source-class inliers)

#### Type II: Multiple source-class inliers

Figure 10 shows the average ranks of the methods over all Type II experiments with respect to ROC AUC, AdjustedPrec@*n*, and MCC. When comparing Type I experiments in Fig. 9 and Type II experiments in Fig. 10, it can be seen that in Type II experiments, which correspond to application scenarios possibly involving multi-modal target classes (inliers), the relative performance of the neighborhood-based outlier detection methods improves, most noticeably the local density-based methods such as LOF and GLOSH, in particular when the statistical significance of the differences in ranks (or lack thereof) are considered. Specifically, in Type II experiments, LOF, GLOSH, and $\hbox {KNN}_{\textit{global}}$ join SVDD, LP, and GMM at the top performing group (among which there is no critical difference observed between the methods) with respect to ROC AUC and AdjustedPrec@*n*. They also remain in the top performing group with respect to MCC, for which the performance of SVDD and LP is once again low. When comparing the average values of the evaluation measures (rather than ranks) between Type I and Type II experiments, the boundary-based and neighborhood-based outlier detection methods tend to remain more stable than other methods, such as GMM, iForest, and ABOD, for which e.g. the average ROC AUC decreased, respectively, from 0.87, 0.83, and 0.8 in Type I experiments to 0.83, 0.75, and 0.72 in Type II experiments (see Table 3 for summarized results and in Section 2 in the Supplementary Material for full details on all experiments).

**Fig. 11:**
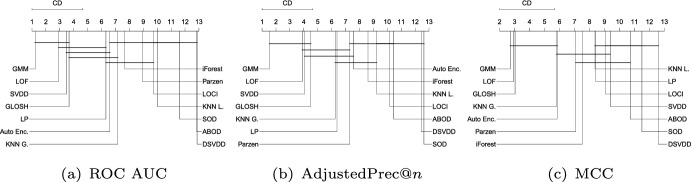
Critical difference diagrams with average ranks of the methods over all Type III experiments (local outliers)

#### Type III: Local outliers

Figure 11 shows the average ranks of the methods over all Type III experiments with respect to ROC AUC, AdjustedPrec@*n*, and MCC. In Type III experiments, which correspond to application scenarios involving local outliers as observations in the vicinity/tail of clusters of inliers (here consisting of truncated multivariate Normal distributions), GMM, LOF, SVDD and GLOSH are the top performers, in this order, with no statistical difference observed between their ranks with respect to ROC AUC and AdjustedPrec@*n*. The relative performance of SVDD as measured by MCC is once again much lower. The fact that GMM outperforms the other methods in every measure can be explained by the fact that the synthetic datasets used in this type of experiment consist of mixtures of Gaussians, which matches the underlying assumption behind GMM models. The good performance of the (non-parametric) LOF and GLOSH models, in turn, can be explained by the fact that these models were specifically designed to detect local outliers. From this perspective, the relative performance of $\hbox {KNN}_{\textit{global}}$ (global method), as expected, has noticeably dropped in Type III experiments as compared to Type I and Type II experiments. It is interesting to note that the outlier detection algorithms SOD (local on subspaces) and ABOD (global), especially designed for high dimensional data, are among the worst performers in this type of scenario involving local outliers, irrespective of the evaluation measure considered.

**Fig. 12:**
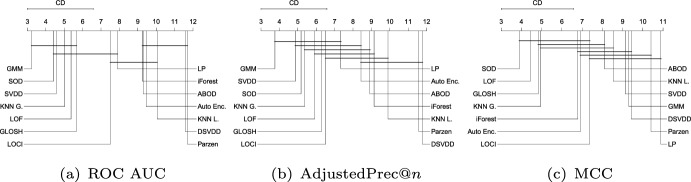
Critical difference diagrams with average ranks of the methods over all Type IV experiments (high-dimensional)

#### Type IV: High-dimensional datasets

Figure 12 shows the average ranks of the methods over all Type IV experiments, involving high-dimensional datasets, with respect to ROC AUC, AdjustedPrec@*n*, and MCC. When comparing these results with Type I experiments in Fig. 9, a noticeable difference can be observed with respect to SOD: while it was only the 9th best performer in Type I experiments with respect to ROC AUC, its relative performance in the high-dimensional scenario rises significantly and it is now the second best with respect to ROC AUC and the best with respect to MCC. The reason for the increased performance of this method is probably associated with the fact that it has been designed to detect outliers in subspaces of higher dimensional spaces. The other method especially designed for high-dimensional datasets, ABOD, only gains slightly in relative performance. However, this relative performance gain is more related to the drop in performance of methods such as Parzen and LP than due to its own improvement (bear in mind, however, that ABOD has no parameters to tune, which may have given the other methods an advantage when setting them up for the evaluation). The LP method works in the similarity space, and the decrease in its performance might be related to the similarity measure used, namely, the Euclidean distance, which is well-known to suffer from the adverse effects of the “curse of dimensionality” in high dimensions. For Parzen, the sample size required to produce a suitable density estimate depends critically on the dimensionality of the space, which might be the reason behind the decrease in its performance.

**Fig. 13:**
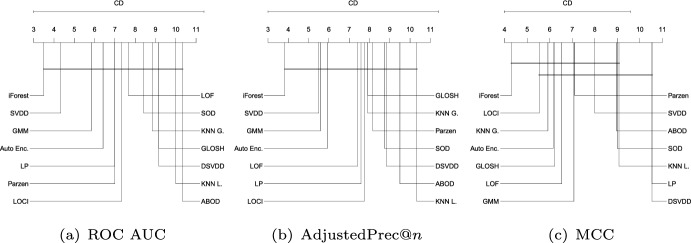
Critical difference diagrams with average ranks of the methods over all Type V experiments (larger sample size)

#### Type V: Larger sample size

Figure 13 shows the average ranks of the methods over all Type V experiments, involving larger datasets, with respect to ROC AUC, AdjustedPrec@*n*, and MCC. When comparing these results with Type I experiments in Fig. 9, a noticeable difference can be observed with respect to iForest: while it was one of the worst performers in Type I experiments, especially with respect to ROC AUC and AdjustedPrec@*n*, its performance in the larger sample size scenario increases significantly and now it is the top performer with respect to all our measures. This improvement is likely due to the sampling procedure performed by the algorithm to build the iTrees, which requires larger amounts of observations for better performance. This conjecture is in line with the observations by the original authors (Liu et al. 2008, 2012); they also noticed that their method performs better when the dataset is sufficiently large (in the case of their experiments, for a sample size larger than 1000). It is worth remarking, however, that there is no statistically significant difference between any methods in this set of experiments, while noting the small number of the datasets used in this experiment (only 13 datasets had a sample size large enough to be included). Although none of the methods worsened their performance when compared to the experiments involving all datasets (ABOD and SOD maintained the same performance), iForest was the one that exhibited the most noticeable performance gain, rising from 0.83 ROC AUC in the scenario involving all the datasets to 0.89 when only datasets with larger sample sizes are used (see Table 3).

### Model selection and comparison of algorithms under practical parameter tuning

The parameter tuning procedure used for the experiments reported in Sect. 6.1 were (as usual in the literature) based on labeled datasets containing both inliers and outliers. While the outliers were not used directly when training the algorithms, they were used to select the algorithms’ optimal parameters by assessing their cross-validated ROC AUC results. As such, the results should be interpreted as comparing the full potential of the different methods should their optimal parameter values be known. However, as previously discussed, unlike controlled experiments such as those conducted in Sect. 6.1, in practical applications of one-class classification this type of supervised model/parameter selection is not very realistic because there may be only a very small amount of outliers available, if any, which will not allow for a proper cross-validation procedure to be carried out. In real applications, parameter selection must be performed using truly one-class model selection methods, such as those described in Sect. 4.1.

In this section, we evaluate the performance of the model selection methods described in Sect. 4.1, namely, SDS, Perturbation, and Uniform Objects, for selecting the best parameter for the different one-class classifiers, which will be compared under this more realistic scenario. In order to avoid clutter and make the critical difference diagrams cleaner, here we only present results for the overall top performers with statistical significance according to the experiments reported in Sect. 6.1. The complete collection of results, including the full critical difference diagrams, can be found in Section 2 in the Supplementary Material.

**Fig. 14:**
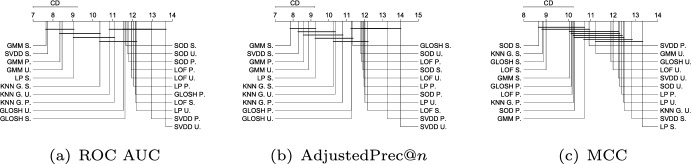
Critical difference diagrams with average ranks of the methods equipped with their best parameter value according to the respective model selection method over all Type I experiments (single source-class inliers). Methods ending in S., P. and U. had their parameters selected by SDS, Perturbation, and Uniform Objects, respectively

Figure 14 shows the average ranks of the methods with the parameters selected by the respective model selection methods over all Type I experiments (single source-class inliers) with respect to ROC AUC, AdjustedPrec@*n*, and MCC. By looking at ROC AUC and AdjustedPrec@*n*, one can see that, when equipped with parameters selected by SDS, the GMM, SVDD, and LP models are placed at the top performing group, similarly to the experiments of Sect. 6.1, but now *without using any ground truth information about outliers*. In contrast, the parameters selected by Perturbation and Uniform Objects make LP and SVDD some of the worst performers, which suggests that these model selection methods may not work well to select parameters for boundary models. When looking at MCC, the overall picture is similar to the optimistic scenario in Sect. 6.1 in that the performance of the boundary models deteriorates significantly, irrespective of the model selection method used. Overall, we can see a clear trend in the models when evaluated using MCC: models selected by SDS (except boundary models) are clustered at the top whereas models selected by Uniform Objects are clustered at the bottom.

The overall picture is similar for the other different scenarios, namely, Type II (multiple source-class inliers), Type III (local outliers), Type IV (high-dimensional datasets), and Type V (larger sample size) experiments. Models selected by SDS usually perform the best, whereas those selected by Uniform Objects tend to comparatively underperform, although there are some occasional inversions. The main conclusions about which methods perform better in each of these scenarios would also not change when selecting the parameters under the practical parameter tuning, which does not use any ground truth information about outliers (the complete collection of results is available in Section 2 in the Supplementary Material).

### Comparison of individual algorithms versus the ensemble approach

As one can see from the results in Sects. 6.1 and 6.2, a single one-class classification algorithm/model cannot outperform the others in every single scenario, and some of them may perform poorly in particular application scenarios. A possible approach to bypassing the selection and use of a single model, whose performance is expected to vary across different datasets, is to rely on an ensemble of multiple models in order to obtain more stable and accurate results.

In this section, we perform three different experiments involving an ensemble: (1) initially, we combine the outputs (outlier scorings as converted into ranks) of all the models evaluated in the experiments shown in Sect. 6.1, using the rank-based ensemble strategy (RRF) described in Sect. 5.6; (2) we then performed a preliminary, controlled experiment where we guided the selection of a subset of base models to be combined into the ensemble using ROC AUC; (3) finally, we performed a more realistic experiment where we select the base members for the ensemble using, in lieu of ROC AUC, the practical model selection methods described in Sect. 4.1, namely, SDS, Perturbation, and Uniform Objects.

#### Ensemble without selection of base models

**Fig. 15:**
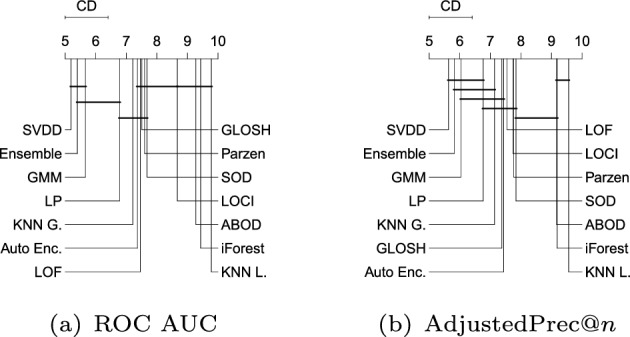
Critical difference diagrams with average ranks of the methods including the ensemble approach

We show the results of our first experiment in Fig. 15, which summarizes the comparison between the resulting ensemble (combining all the individual models) against every individual model. Notice that this experiment and results involve the “optimistic” approach for model selection (parameter tuning) adopted in Sect. 6.1. Also, notice that MCC has not been displayed as it cannot be straightforwardly computed in a meaningful way for the ensemble: while MCC computation requires a binary inlier/outlier classification output, the ensemble just combines the multiple rankings of outlier scores produced by the different models, each of which adopts a different decision boundary/threshold to perform classifications, and these classifications cannot be combined by the ensemble using the rank-based strategy adopted here.

When comparing the performance of the individual methods against the ensemble in Fig. 15, one can see that although the ensemble appears among the top performers, SVDD and GMM perform on average as well as the ensemble, with no statistical difference. Hence, from a computational standpoint, SVDD and GMM might be preferred as they exhibit good overall performance while requiring training of a single model. On the other hand, one advantage of the ensemble approach is that by combining multiple results it can reduce the variance of the individual performances across different applications. For example, we have 6 occurrences of values below 0.7 of ROC AUC for SVDD and GMM, in contrast to only 3 for the ensemble (see Table 6). In addition, we will show below that the performance of the ensemble can be further improved by proper selection of ensemble members.

**Table 6:** Datasets that displays ROC AUC values below 0.7 for the individual methods SVDD and GMM and for the ensemble that combines the results of all 14 methods

ROC AUC	GMM	SVDD	Ensemble
Cancer	**0**.**51**	0.75	0.72
Cardiotocography	0.98	**0**.**65**	0.78
Cellcycle	**0**.**65**	**0**.**68**	0.76
Colon	**0**.**64**	**0**.**59**	0.78
Diabetes	**0**.**62**	**0**.**67**	0.71
Hayes-roth	**0**.**69**	0.73	**0**.**68**
Heart	0.85	**0**.**55**	**0**.**63**
Hepatitis	0.84	**0**.**51**	**0**.**51**
Survival	**0**.**67**	0.77	0.76

Values below 0.7 are shown in bold

#### Optimistic selection of base models

**Fig. 16:**
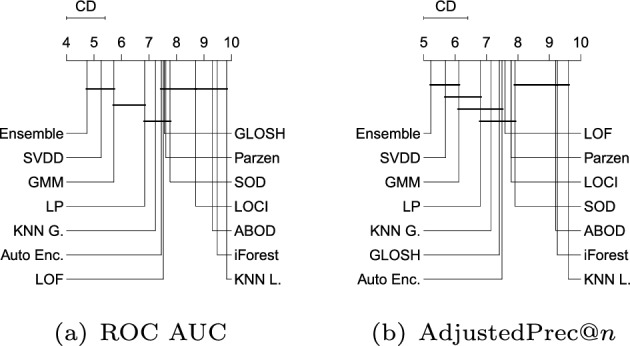
Critical difference diagrams with average ranks of the methods including the ensemble approach: ensemble members selected using ROC AUC

As previously discussed in Sect. 2.3, there are two key properties that an ensemble should have: diversity and accuracy. Although combining all available models can increase diversity, some of the individual models may not be accurate enough to positively contribute to the combined result as ensemble members. To confirm this hypothesis, we initially performed a preliminary, controlled experiment where we combined into the ensemble only the base models that exhibited ROC AUC values higher than the average across all models. The result is displayed in Fig. 16, which displays a noticeable gain in performance for the ensemble (even though there is still no statistical difference in the ranks when compared to SVDD and GMM). When comparing the evaluation measures for the ensemble approach without and with selection of ensemble members, there is an increase of average ROC AUC from 0.87 to 0.89 and also an increase of average AdjustedPrec@*n* from 0.58 to 0.64.

#### Practical selection of base models

The aforementioned improvement in ensemble performance, however, may not be achievable in practical applications of one-class classification as it requires outliers for selection of base members using ROC AUC. As an attempt to overcome this limitation, we performed additional experiments where we select base members for the ensemble using, in lieu of ROC AUC, the practical model selection methods described in Sect. 4.1, namely, SDS, Perturbation, and Uniform Objects. These methods were used to select the best parameter(s) for each model in Sect. 6.2, and now they are used again to also select the best of those models/results (i.e., those above average) to be combined into the ensemble.

**Fig. 17:**
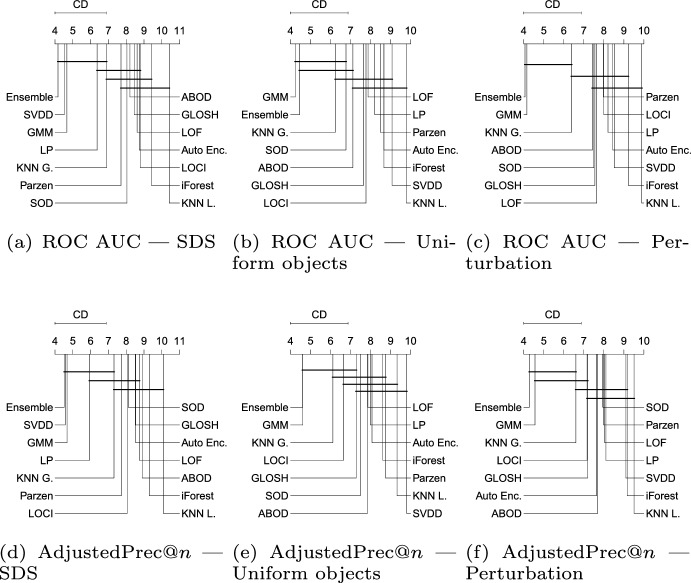
Critical difference diagrams with average ranks of the methods including the ensemble approach: ensemble members selected using practical model selection strategies

Figure 17 displays the results. Similarly to our preliminary, controlled experiment, the ensemble is consistently a top performer irrespective of the evaluation measure considered, and also irrespective of the model selection method used to select ensemble members and their parameters. For SDS, the individual models GMM, SVDD, LP, and $\hbox {KNN}_{\textit{global}}$ are overall competitive against the ensemble, with no statistically significant difference observed between their average ranks. In the case of Perturbation, only GMM and $\hbox {KNN}_{\textit{global}}$ are overall statistically comparable to the ensemble. Here it is worth noticing that, as previously discussed, Perturbation did not perform well in selecting parameters for boundary models, so the statistical difference observed between the ensemble and these particular models is at least in part explained by the poor performance of these individual models when equipped with parameters selected by Perturbation. As for the Uniform Objects method, depending on the evaluation measure considered there is a number of individual models exhibiting no statistically significant difference from the ensemble, namely, GMM, $\hbox {KNN}_{\textit{global}}$, SOD, LOCI, and GLOSH. Once again, it is worth noticing that the statistically significant difference between the ensemble and the boundary methods LP and SVDD is likely due to the same reason explained above for Perturbation.

**Fig. 18:**
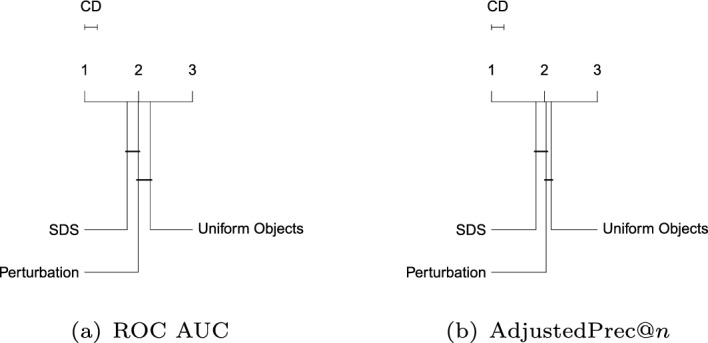
Critical difference diagrams with average ranks of the ensemble approaches: ensemble members selected using the different practical model selection strategies

Overall, when comparing ensemble combinations using the different model selection methods to select ensemble members and their parameters (Fig. 18), ensembles built based on SDS usually perform best, while those using Uniform Objects usually have the worst performance, in agreement with our previous results involving model selection experiments in Sect. 6.2.

## Conclusion

In this paper, we performed and reported a comprehensive comparison of one-class classification algorithms/models as well as unsupervised outlier detection methods extended to the one-class classification scenario. These methods were evaluated over a large number of datasets, measuring performance with respect to ROC AUC, AdjustedPrec@*n*, and MCC in different experimental settings. We also evaluated the use of three model selection techniques for one-class classification to select models and their parameters when only labeled observations of the inlier class are available. Finally, we studied how an ensemble of one-class classifiers compares against individual based models, particularly when selecting ensemble members by using one-class model selection methods.

Regarding the studied models, a noticeable result is that methods specifically designed for one-class classification usually perform better in the scenarios studied. More specifically, **GMM** and **SVDD** appear **among the top performers in most scenarios**, and as such they can be sensible choices when there is very little or no information about the characteristics of the particular application scenario in hand. However, **in specific scenarios** unsupervised outlier detection methods can become very competitive and appear **among the top performers**, for example: locally sensitive density-based outlier detection methods such as **GLOSH and LOF in scenarios with local outliers as well as in scenarios with a possibly multi-modal target class of inliers; SOD for scenarios involving high-dimensional datasets; and iForest for large datasets**. These conclusions contrast to the previous comparison study by Janssens et al. (2009), which evaluated only the scenario of Single Source-Class Inliers. The authors reported SVDD, LOF, and $\hbox {kNN}_{\textit{local}}$ as the top-performers. We, however, could only confirm the top performance of SVDD. In addition, **SVDD only** appears as a **top performer when considering the problem of outlier/inlier ranking** (as assessed by ROC AUC and AdjustedPrec@*n*), but **not the binary outlier/inlier classification problem** (as assessed by MCC). This result may suggest that the classification threshold imposed by this algorithm is not necessarily suitable despite the quality of the model in terms of ranking outliers above inliers.

It is also important to acknowledge that we do not evaluate the algorithms in all possible application scenarios. Therefore, although some methods did not perform well in any of the scenarios comprised in our benchmark, they might perform well in other scenarios. For example, deep-learning methods have been shown to be effective on large and complex datasets in specialised domains (Ruff et al. 2018, 2020), which are not considered here. In the scenarios studied here, the traditional algorithms have shown to be more effective. This conclusion is in agreement with Ruff et al. (2020), who also reported the advantage of shallow over deep approaches in tabular data.

Regarding the **practical model selection** methods, the most noticeable result is that **SDS** appears to be the **top choice regardless of the scenario or measure**, consistently selecting the best models in the scenarios studied, while Uniform Objects consistently performed the worst. This finding extends the conclusion of Wang et al. (2018), who reported SDS as the top-performer among the methods studied for hyperparameter selection of OCSVM (Schölkopf et al. 2001). In our more comprehensive comparative study, we also confirmed the top performance of SDS (if not in first, very close and without statistical difference with respect to the first) when selecting parameters for the 14 different algorithms studied on the 5 different application scenarios considered, as well as when guiding the selection of ensemble members.

When comparing the **practical parameter tuning against the optimistic one** (which uses ground-truth labels from both classes), we can see that the **same methods are placed similarly at the top performing group, regardless of the tuning approach** adopted. This suggests that **effective model selection can be achieved in practice** in the absence of outliers in the training dataset. Another important conclusion regarding the model selection methods is the ineffectiveness of Perturbation and Uniform Objects when selecting parameters for boundary methods such as SVDD and LP.

Finally, the ensemble approach has proven a robust and effective choice when compared to individual classifiers. Our most important conclusion regarding **ensembles** is that a **guided selection of base members is effective to enhance performance**, and it **can be achieved in practice by using model selection techniques** for one-class classification. In agreement with the conclusions above regarding model selection, **ensembles built based on SDS usually perform best**, with statistical significance when compared to Uniform Objects.

Although the ensemble approach has proven a robust and effective choice, in the context of ensembles, we only investigate how to select accurate one-class classifiers. There are two other central questions to build good ensembles: How to introduce diversity into the ensembles and how to combine the results. Therefore, two interesting directions for future research are: (1) a comprehensive comparative study of different score-based and rank-based combination strategies; (2) a comprehensive study of a different strategy to introduce diversity, for example, using the same base model (e.g., GMM and/or SVDD), but with different variations of the data (e.g., subsampling and/or subspaces).

## Supplementary Information

Below is the link to the electronic supplementary material.

Supplementary file 1 (pdf 860 KB)

## Data Availability

We made publicly available all the codes and datasets used at https://github.com/homarques/occ.

## Footnotes

1 Note that only the linear SVDD can be efficiently computed in ${\mathcal {O}}(N)$; if other kernels were to be adopted, the SVDD computation would be ${\mathcal {O}}(N^3)$.

2 Note that although the unsupervised outlier detection algorithms usually do not provide this threshold, when they are applied to the framework for one-class classification discussed in Sect. 3.3 this threshold is provided as a by-product of an arbitrary choice of $\alpha$.

3 In the case of parameter(s) that result in the same ROC AUC value, we select the parameter(s) that result in the best MCC value. If the tie persists, we look at AdjustedPrec@n.

4 It is beyond the scope of this paper to investigate/compare different score-based or rank-based combination strategies. We use RRF because it has been shown before as one of the top-performers for ensemble combinations (Jaskowiak et al. 2016).
