# Supplementary material for: On the evaluation of outlier detection and one-class classification: a comparative study of algorithms, model selection, and ensembles
Source: Data Min Knowl Discov. 2023 May 16;37(4):1473–517. doi: 10.1007/s10618-023-00931-x (PMC10326160; doi:10.1007/s10618-023-00931-x)
Supplement: Supplementary file 1 — (pdf 860 KB) [file 10618_2023_931_MOESM1_ESM.pdf]

On the Evaluation of Outlier Detection and One-Class Classification: A Comparative Study of Algorithms, Model Selection, and Ensembles [Supplementary Material]

Henrique O. Marques[1*], Lorne Swersky[2], Jörg Sander[2], Ricardo J. G. B. Campello[1] and Arthur Zimek[1]

[1]University of Southern Denmark, Denmark.
[2]University of Alberta, Canada.

*Corresponding author(s). E-mail(s): oli@sdu.dk;
Contributing authors: jsander@ualberta.ca;
campello@imada.sdu.dk; zimek@imada.sdu.dk;

1 Methods and their Properties

1.1 Description of the Methods

Gaussian Mixture Model (GMM)

The most widely used parametric model is the Gaussian distribution (Bishop, 2007), where, in the most simple case, a single Gaussian probability density function,

$$p_{Gauss}(\mathbf{x}|\boldsymbol{\mu}, \Sigma) = \frac{1}{(2\pi)^{d/2}|\Sigma|^{1/2}}\, \mathrm{e}^{-\frac{1}{2}(\mathbf{x}-\boldsymbol{\mu})^T\Sigma^{-1}(\mathbf{x}-\boldsymbol{\mu})}, \tag{1}$$

is fit to the inlier data, with $\boldsymbol{\mu}$ being the mean, Σ being the covariance matrix, and d being the dimensionality of the data. Using a single Gaussian distribution, however, makes a very strong assumption about the distribution being unimodal, which is often violated.

A more flexible model is a Gaussian Mixture Model (GMM) (Bishop, 2007), where the distribution is assumed to be the result of a mixture of

different Gaussian distributions. The means μ_i and covariances Σ_i of the individual Gaussian components can efficiently be estimated by an Expectation-Minimization (EM) algorithm (Dempster et al, 1977). Aside from the inherited disadvantages of parametric methods, the main disadvantages of the Gaussian density are related to the covariance matrix. For high dimensional data, this model suffers from very large covariance matrices, which makes it expensive to compute its inverse. In case the inverse of the covariance matrix cannot be calculated (*e.g.* data with singular directions), it has to be approximated, for example, by adding a small constant ϵ to the diagonal, or alternatively, approximated by its pseudo-inverse (Strang, 2016).

Parzen Window (PW)

PW is a non-parametric method based on Parzen Density Estimation (Parzen, 1962) that estimates the density of the data using a mixture of kernels centered on each of the N individual training instances. In our study, we use Gaussian kernels with diagonal covariance matrices $\Sigma_i = hI$, where h is a width parameter. The probability of an instance being an inlier is then computed as:

$$\mathrm{PW}(\mathbf{x}) = \frac{1}{N}\sum_{i=1}^{N} p_{Gauss}(\mathbf{x}|\mathbf{x}_i, hI) \tag{2}$$

The training for the Parzen density consists of the determination of the parameter h, which can be optimized using the maximum likelihood solution (Duin, 1976). Alternatively, the user can supply the parameter h. In the latter case, the computational cost for training is negligible. Testing new instances, however, is computationally expensive. During the testing phase, the distances of the new instance to the training instances have to be computed, which also imposes a storage limitation, since the training instances have to be stored and typically large training sets are required to produce a good density estimation.

Support Vector Data Description (SVDD)

SVDD (Tax and Duin, 2004) is a boundary-based one-class classification method inspired by Support Vector Machines (SVM) (Vapni, 1995) used in regular classification problems. The primary difference between SVDD and SVM is that while a SVM attempts to separate two or more classes with a maximum margin hyperplane, SVDD instead will enclose the inlier class in a minimum volume hypersphere by minimizing the following error:

$$\mathcal{E}(R, \mathbf{a}, \boldsymbol{\xi}) = R^2 + C\sum_i \xi_i, \tag{3}$$

subject to the constraints:

$$\|\mathbf{x}_i - \mathbf{a}\|^2 \leq R^2 + \xi_i, \quad \xi_i \geq 0, \quad \forall i, \tag{4}$$

where R is the radius of the hypersphere, $\mathbf{a}$ is the center of the hypersphere, $\boldsymbol{\xi}$ are slack variables allowing training observations $\mathbf{x}$ to fall outside the SVDD boundary, and C is a penalty (regularization) parameter.

Like traditional SVMs, the above formulation can also be extended to non-linearly transformed spaces using kernel methods. In the case of a kernel with the property $K(\mathbf{x}_i, \mathbf{x}_i) = 1$, $\forall_i$, such as the Gaussian kernel that we use in our experiments, OC-SVM (Schölkopf et al, 2001) and SVDD (Tax and Duin, 2004) find the same decision boundary when some conditions are met (Tax and Duin, 2004; Schölkopf et al, 2001; Tax, 2001).

Linear Programming (LP)

LP (Pekalska et al, 2002) is a boundary method which, instead of using the explicit feature space, utilizes a dissimilarity measure to compare new instances to the inlier instances in the training set. The basic assumption is that instances belonging to the inlier class are similar to each other, while the outliers are dissimilar to inliers. The use of a dissimilarity measure makes the method very convenient for applications where it is difficult to define suitable features for other approaches, as *e.g.* in unstructured data (strings, graphs or shapes). The dissimilarity measure used must meet the criteria defined by the authors: reflectivity, positivity, and symmetry. LP constructs the boundary by minimizing the volume of a simplex (Bazaraa et al, 2009) with the main vertex being the origin and the other vertices resulting from the intersection of the boundary and the axes of the dissimilarity space.

For unbounded dissimilarity measures, instances with large dissimilarities values (due to noise or the existence of outliers in the training data) may affect the decision boundary. In order to make the classifier robust against such values, the dissimilarities are transformed by the sigmoid function, $sigm(x) = \frac{2}{1+e^{x/s}}$, such that large values are bounded by 1. In this case, the user has to define the parameter s for the slope of the sigmoid function.

k-Nearest Neighbor Data Description (kNN$_{local}$)

The k-Nearest Neighbor Data Description approach (de Ridder et al, 1998), which we call here kNN$_{local}$, avoids explicit density estimations by only using the distances to the nearest neighbors. It resembles local outlier detection methods in that it approximates the local (normalized) distance of the training instances, however in a simpler way. An observation is classified under kNN$_{local}$ by computing the ratio between the distance from an observation to its kth nearest neighbor $\text{NN}_\text{k}(\mathbf{x}_i)$, and the distance between the kth nearest neighbor and that neighbor's k^{th} nearest neighbor:

$$\text{kNN}_{local}(\mathbf{x}_i, k) = \frac{d(\mathbf{x}_i, \text{NN}_\text{k}(\mathbf{x}_i))}{d(\text{NN}_\text{k}(\mathbf{x}_i), \text{NN}_\text{k}(\text{NN}_\text{k}(\mathbf{x}_i)))} \quad (5)$$

kNN$_{local}$ has the same computational issues when testing new instances as PW. However, according to Tax (2001) the method performs best in small

sample size, where overall computational efforts are low. For larger sample sizes and noisy data, reported quality of results is rather poor (Tax, 2001).

Auto-Encoder Networks

An Auto-Encoder (Japkowicz et al, 1995; Tax, 2001) is a neural network with a sigmoidal transfer function, a single hidden layer, and a parameter-defined number of hidden units trained on the inlier class. The network is trained to reproduce the input patterns at its output layer (*i.e.*, it should perform the identity operation) by minimizing the Mean Square Error (MSE). In order to classify a new instance, the instance is given as input to the network and the difference between the original input and the network's output defines the reconstruction error.

An Auto-Encoder can learn very flexible models. However, it suffers from the same problems as the conventional neural networks for classification problems, such as the high number of parameters to tune (learning rate, number of neurons, number of layers, number of epochs, etc), local minimum, weight initialization, etc. (Freeman and Skapura, 1991; Goodfellow et al, 2016).

Deep SVDD

Deep SVDD (Ruff et al, 2018) is an end-to-end deep learning approach for one-class classification which jointly trains a deep neural network while optimizing a data-enclosing hypersphere of the smallest size in output space. In contrast to kernel-based SVDD, Deep SVDD learns useful feature representations of the data together with the one-class classification objective by employing a neural network that is jointly trained to map the data into a hypersphere of minimum volume. The Deep SVDD minimizes the following objective function:

$$\frac{1}{N}\sum_{i=1}^{N}\|\phi(\mathbf{x}_i, W) - c\|^2 + \frac{\delta}{2}\sum_{j=1}\|W^j\|^2, \delta > 0, \tag{6}$$

where ϕ is the neural network with the corresponding set of weights W trained to learn a transformation that minimizes the volume of a data-enclosing hypersphere centered on a predetermined point c. The second term is a standard weight decay regularizer.

Local Outlier Factor (LOF)

LOF (Breunig et al, 2000) is an unsupervised outlier detection method that, similarly to kNN$_{local}$, compares the local density of an observation to that of its neighbors. The distances between observations are replaced by reachability distances, defined as:

$$\text{reach-dist}_k(\mathbf{x}_i \leftarrow \mathbf{x}_j) = \max\{d(\mathbf{x}_j, \text{NN}_k(\mathbf{x}_j)), d(\mathbf{x}_i, \mathbf{x}_j)\} \tag{7}$$

The local reachability density of an observation $\mathbf{x}_i$ is then defined as the inverse average reachability distance from the set of $\mathbf{x}_i$'s neighbors, $k\text{NN}(\mathbf{x}_i)$, that are within the k nearest neighbor distance around $\mathbf{x}_i$:

$$\mathrm{lrd_k}(\mathbf{x}_i) = \frac{|\,k\mathrm{NN}(\mathbf{x}_i)|}{\sum_{\mathbf{x}_j \in k\mathrm{NN}(\mathbf{x}_i)} \text{reach-dist}_k(\mathbf{x}_i \leftarrow \mathbf{x}_j)} \tag{8}$$

Finally, the LOF score of an observation is computed by comparing the lrd of the observation with that of its neighbors:

$$\mathrm{LOF}_k(\mathbf{x}_i) = \frac{\sum\limits_{\mathbf{x}_j \in k\mathrm{NN}(\mathbf{x}_i)} \frac{\mathrm{lrd}_k(\mathbf{x}_j)}{\mathrm{lrd}_k(\mathbf{x}_i)}}{|\,k\mathrm{NN}(\mathbf{x}_i)|} \tag{9}$$

Local Correlation Integral (LOCI)

LOCI (Papadimitriou et al, 2003) is an unsupervised outlier detection method which analyzes the density of an observation at multiple neighborhood radii φr of a given maximum radius r, where $\varphi \in (0, 1]$. For each observation $\mathbf{x}_i$, a (local) r-neighborhood $\mathcal{N}(\mathbf{x}_i, r) = \{\mathbf{x} | d(\mathbf{x}_i, \mathbf{x}) \leq r\}$ and a (local) r-density $n(\mathbf{x}_i, r) = |\mathcal{N}(\mathbf{x}_i, r)|$ are defined.

The average φr-density inside a r-neighborhood around an observation $\mathbf{x}_i$ is then defined as:

$$\hat{n}(\mathbf{x}_i, r, \varphi) = \frac{\sum_{\mathbf{x}_j \in \mathcal{N}(\mathbf{x}_i, r)} n(\mathbf{x}_j, \varphi r)}{n(\mathbf{x}_i, r)}, \tag{10}$$

and the multi-granularity deviation factor (MDEF) is given by:

$$\mathrm{MDEF}(\mathbf{x}_i, r, \varphi) = 1 - \frac{n(\mathbf{x}_i, \varphi r)}{\hat{n}(\mathbf{x}_i, r, \varphi)} \tag{11}$$

An observation $\mathbf{x}_i$ is classified using the following score:

$$\sigma\,\mathrm{MDEF}(\mathbf{x}_i, r, \varphi) = \frac{\sigma_{\hat{n}}(\mathbf{x}_i, r, \varphi)}{\hat{n}(\mathbf{x}_i, r, \varphi)}, \tag{12}$$

which is the normalized standard deviation $\sigma_{\hat{n}}(\mathbf{x}_i, r, \varphi)$ of $n(\mathbf{x}_i, \varphi r)$ for $\mathbf{x}_i \in \mathcal{N}(\mathbf{x}_i, r)$. With these quantities, the LOCI score is computed as follows:

$$\mathrm{LOCI}(\mathbf{x}_i, \varphi) = \max_{r \in \mathcal{R}} \left\{ \frac{\mathrm{MDEF}(\mathbf{x}_i, r, \varphi)}{\sigma\,\mathrm{MDEF}(\mathbf{x}_i, r, \varphi)} \right\} \tag{13}$$

k-Nearest Neighbor (kNN$_{global}$)

The k-Nearest Neighbor approach, which we call here kNN$_{global}$, has been originally introduced as an unsupervised distance-based outlier detection method (Ramaswamy et al, 2000). Its score is the numerator of Equation (5):

$$\mathrm{kNN}_{global}(\mathbf{x}_i, k) = d(\mathbf{x}_i, \mathrm{NN_k}(\mathbf{x}_i)), \tag{14}$$

which makes the score global rather than local.

Angle-Based Outlier Detection (ABOD)

ABOD (Kriegel et al, 2008) is a global outlier detection algorithm which uses not only the distances between points but primarily the variance of the angles between points. ABOD computes the variance of the angles between point $\mathbf{x}_i$ and all other pairs of points in the dataset $\mathbf{X}$, weighted by the inverse of the distances to the respective points. This weighting factor is important since the angle to a pair of points varies naturally more for larger distances. The Angle-Based Outlier Factor (ABOF) is defined as follows:

$$\mathrm{ABOF}(\mathbf{x}_i) = \mathrm{VAR}_{\mathbf{x}_j,\mathbf{x}_k \in \mathbf{X}_{\mathrm{train}}} \left(\frac{\langle \mathbf{x}_i - \mathbf{x}_j, \mathbf{x}_i - \mathbf{x}_k \rangle}{\|\mathbf{x}_i - \mathbf{x}_j\|^2 \cdot \|\mathbf{x}_i - \mathbf{x}_k\|^2} \right) \tag{15}$$

Subspace Outlier Degree (SOD)

SOD (Kriegel et al, 2009) analyzes for each object how well it fits into the subspace that is spanned by a set of reference objects. In order to define the reference set, the authors suggest using Shared Nearest Neighbors (SNN) in the full space. Once this reference set has been defined, the *relevant subspace* is determined as the set of attributes in which the variance of the objects of the reference set to its center is small compared to the expected variance. The outlier scoring of SOD is computed as the Euclidean distance of an object to the center of the reference set in the relevant subspace, normalized by the number of relevant attributes.

Global-Local Outlier Scores from Hierarchies (GLOSH)

GLOSH (Campello et al, 2015) is an unsupervised outlier detection algorithm based on the hierarchical density estimates provided by the hierarchical clustering algorithm HDBSCAN*. After a density-based clustering hierarchy is computed for the whole dataset, the GLOSH score for each observation $\mathbf{x}_i$ can be computed based on the difference in density around $\mathbf{x}_i$ and the highest density inside the cluster closest to $\mathbf{x}_i$ (from a density-connectivity perspective) in the HDBSCAN* hierarchy, defined as follows:

$$\mathrm{GLOSH}(\mathbf{x}_i) = \frac{\lambda_{\max}(C_{\mathbf{x}_i}) - \lambda(\mathbf{x}_i)}{\lambda_{\max}(C_{\mathbf{x}_i})}, \tag{16}$$

where $\lambda(\mathbf{x}_i)$ is the density of $\mathbf{x}_i$ and $\lambda_{\max}(C_{\mathbf{x}_i})$ is the highest density of an observation inside the closest cluster $C_{\mathbf{x}_i}$, where densities are estimated by a k-nearest neighbor density estimator. The closest cluster $C_{\mathbf{x}_i}$ is the one that $\mathbf{x}_i$ belongs to at the density level of $\mathbf{x}_i$.

To apply GLOSH in a one-class classification scenario, we can construct initially the HDBSCAN* hierarchy using the training data, and then use this hierarchy as a fixed "model" to compute outlier scores for unseen data. In order to classify a new instance $\mathbf{x}_i$, we must determine which is the closest cluster $C_{\mathbf{x}_i}$ in the fixed hierarchy. This can be achieved by first adding a given instance $\mathbf{x}_i$ to the Minimum Spanning Tree (MST) which underlies the

HDBSCAN* hierarchy; $\mathbf{x}_i$ is connected to the training instance $\mathbf{x}_j$ with the smallest "distance" in the density space in which the MST is constructed. We can find the closest cluster $C_{\mathbf{x}_i}$ for an instance $\mathbf{x}_i$ by removing the edges of the MST in decreasing order of weight. The closest cluster $C_{\mathbf{x}_i}$ is the one that $\mathbf{x}_i$ belongs before being detached from the MST.

There are two situations that may occur when connecting the new instance $\mathbf{x}_i$ to the training instance $\mathbf{x}_j$. The first situation is that the edge connecting $\mathbf{x}_i$ and $\mathbf{x}_j$ is removed before $\mathbf{x}_j$ is separated from the MST. In this case, the closest cluster to $\mathbf{x}_i$ will be the cluster that $\mathbf{x}_j$ belongs to when $\mathbf{x}_i$ is detached. We show an example of this situation in Figure 1.

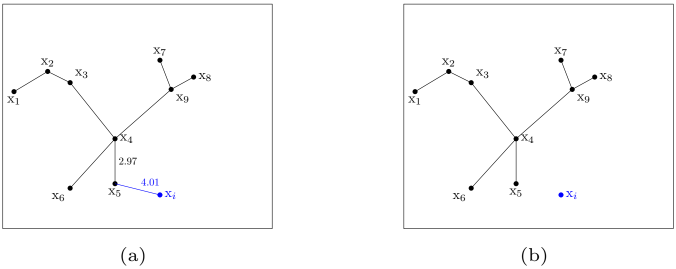

Fig. 1: MST which underlies the HDBSCAN* hierarchy in the density space with minimum cluster size (m_{clSize}) = 3. The new instance $\mathbf{x}_i$ is connected to the training instance with the smallest distance $\mathbf{x}_5$ (Figure 1(a)). The edge connecting $\mathbf{x}_i$ and $\mathbf{x}_5$ is removed before $\mathbf{x}_5$ is separated from the MST and so the closest cluster to $\mathbf{x}_i$ is the one $\mathbf{x}_5$ is assigned at this density level. In this case, it is the cluster where all instances remain clustered (Figure 1(b))

The second situation is that $\mathbf{x}_j$ is separated from the MST before the edge between $\mathbf{x}_i$ and $\mathbf{x}_j$ is removed. In this case, the closest cluster for $\mathbf{x}_i$ is the same as the closest cluster for $\mathbf{x}_j$. For this situation, we show an example in Figure 2.

Once we have identified the closest cluster for $\mathbf{x}_i$, we can compute its GLOSH score using Equation (16).

Isolation Forest (iForest)

iForest (Liu et al, 2008, 2012) is an unsupervised outlier detection algorithm based on the concept of isolation which can be seen as a particular kind of density estimate. The concept of isolation in this context means "separating an instance from the rest of the instances", which is achieved by building an ensemble of binary trees called isolation trees (iTree). An iTree recursively divides a dataset by randomly selecting an attribute and a split value until either the resulting partitions/nodes have only one instance or all instances in a partition/node has the same value. Anomalies are more likely to be isolated

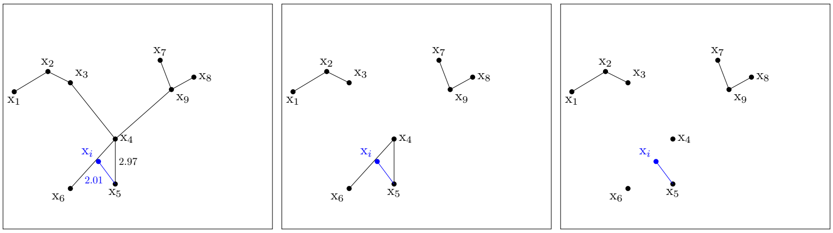

(a) (b) (c)

Fig. 2: MST which underlies the HDBSCAN* hierarchy in the density space with minimum cluster size (m_{clSize}) = 3. The new instance $\mathbf{x}_i$ is connected to the training instance with the smallest distance $\mathbf{x}_5$ (Figure 2(a)). The edge connecting $\mathbf{x}_5$ is separated from the MST before the edge between $\mathbf{x}_i$ and $\mathbf{x}_5$ (Figure 2(c)) and so the closest cluster to $\mathbf{x}_i$ is the closest cluster for $\mathbf{x}_5$. In this case, it is the cluster with the instances $\mathbf{x}_4$, $\mathbf{x}_5$ and $\mathbf{x}_6$ (Figure 2(b))

closer to the root of an iTree, whereas normal instances are more likely to be isolated at deeper levels of an iTree. Therefore, anomalies are those instances that have short average path lengths in the iTrees.

1.2 Time Complexity

The computational complexity of the one-class classification algorithms can be analyzed for the two different sub-tasks: the training time $\mathcal{O}(f_{tr}(N))$ and the testing (or consultation) time $\mathcal{O}(f_{te}(M))$, as a function of the training set size (N) and the test set size (M), respectively. In general, the algorithms can be divided into two groups, eager learning and lazy learning (Webb, 2011). In regular classification problems, the majority of computation of eager learning algorithms occurs at training time, while lazy learning algorithms spend more time when testing. In one-class classification, however, if one wants to determine the threshold to separate inliers from outliers when using lazy learning algorithms, the model should also be applied to the training data (for example, as discussed in Section 3.3), which can result in higher complexity in the training time than in the testing when $N > M$. On the other hand, if one is not interested in labeling, but only in the ranking, the complexity for training time is usually null[1]. Another disadvantage of lazy learning algorithms is the fact that the training data has to be stored for consultation during the testing phase, which also imposes a storage restriction.

In Table 1, we provide the complexity of the methods discussed here. For networks such as Auto-encoder and Deep SVDD, the training time complexity depends on the training algorithm used, but it is usually linear in the training set size (N) per each training iteration. Some algorithms, such as the Stochastic Gradient Descent (SGD) methods (Goodfellow et al, 2016), can perform the

[1]For some methods, we still have to apply the model in the training data anyway, for example, for LOF adapted to OCC, we still have to pre-compute the required quantities.

Table 1: Time Complexity of Methods.
N: training set size, M: test set size, d: dataset dimensionality, t: method parameter. *per iteration

	Training time $\mathcal{O}(f_{tr}(N))$	Testing time $\mathcal{O}(f_{te}(M))$
Auto Enc.	$\mathcal{O}(N(t+d+2dt))*$	$\mathcal{O}(dM)$
DSVDD	$\mathcal{O}(t)*$	$\mathcal{O}(dM)$
GMM	$\mathcal{O}(d^3Nt+d^2Nt)$	$\mathcal{O}(d^3Mt)$
iForest	$\mathcal{O}(t_1^2t_2)$	$\mathcal{O}(Mt_1)$
LP	$\mathcal{O}(dN^3)$	$\mathcal{O}(dM)$
SVDD	$\mathcal{O}(dN^3)$	$\mathcal{O}(dM)$
ABOD	$\mathcal{O}(dN^3)$	$\mathcal{O}(dMN^2)$
GLOSH	$\mathcal{O}(dN^2)$	$\mathcal{O}(dMN)$
kNN$_{global}$	$\mathcal{O}(dN^2)$	$\mathcal{O}(dMN)$
kNN$_{local}$	$\mathcal{O}(dN^2)$	$\mathcal{O}(dMN)$
LOCI	$\mathcal{O}(dN^3)$	$\mathcal{O}(dMN^2)$
LOF	$\mathcal{O}(dN^2)$	$\mathcal{O}(dMN)$
PW	$\mathcal{O}(dN^2)$	$\mathcal{O}(dMN)$
SOD	$\mathcal{O}(dN^2)$	$\mathcal{O}(dMN)$

iteration in time complexity less than N, by using only a sample of the training data to compute an unbiased estimate of the gradient. Most deep learning methods, such as Deep SVDD, use SGD to alleviate the computational burden caused by deep number of layers/neurons. The bottleneck in the training of a neural network is usually on the number of weights to train. While for Deep SVDD, there is no predetermined number of weights, for Auto-encoder, the number of weights is equal to $(t + d + 2dt)$, where d is the dimensionality of the dataset, and t is the user-defined number of neurons. Therefore, the use of Auto-encoder for high-dimensional datasets can be expensive. The time complexity in the number of weights also depends on the training algorithm to be used, for example, in the case of Conjugate Gradient, it is linear, while for Levenberg-Marquardt, it is cubic (LeCun et al, 1998). The convergence for Levenberg-Marquardt, however, is usually much faster, but the high time complexity makes it suitable only for small network sizes. Note that, although the time complexity per iteration for Deep SVDD ($\mathcal{O}(\#weights)$) is smaller when compared to autoencoder ($\mathcal{O}(N \times \#weights)$) due to the SGD, the number of iterations required for the convergence of the autoencoder is smaller (Goodfellow et al, 2016). Also, usually the number of weights of the Deep SVDD $\gg$ number of weights of the autoencoder.

The training for GMMs consists of finding the distribution parameters of the Gaussians (covariance matrices and means). It is usually achieved using an Expectation-Maximization (EM) algorithm. For each iteration of the EM algorithm, the E-step takes $\mathcal{O}(d^3Nt)$ and the M-step takes $\mathcal{O}(d^2Nt)$, where d is the dimensionality of the dataset, and t is the user-defined number of clusters. Noticing that, due to the inversion of the covariance matrix ($\mathcal{O}(d^3)$), this

algorithm is expensive for high-dimensional datasets. For testing, the computation of the likelihood of each testing point belonging to the clusters takes $\mathcal{O}(d^3Mt)$.

The training time complexity for iForest uses only a user-defined sampling t_1 of the dataset to build the iTrees. The time complexity to build each of the t_2 iTrees defined by the user is quadratic in t_1, which gives an overall complexity for training equal to $\mathcal{O}(t_1^2 t_2)$. The structure of the iTrees is equivalent to that of Binary Search Trees (BST), which makes the expected time complexity for testing a new instance $\mathcal{O}(\log t_1)$, but the worst-case is $\mathcal{O}(t_1)$.

Both LP and SVDD have to solve an optimization problem during the training phase. While LP has to solve a linear programming (LP) problem, SVDD has to solve a quadratic programming (QP) problem. Both problems can be solved in polynomial time ($\mathcal{O}(N^3)$) using, for example, Interior Point Methods (Woodsend, 2009; Campbell and Bennett, 2000). When using a linear kernel, however, some algorithms in the literature can solve the problem efficiently in linear time (Joachims, 2006; Erfani et al, 2015).

For ABOD, the time complexity relies on the computation of the angles between each point and all other pairs of points in the dataset, which makes the overall time complexity for training $\mathcal{O}(N^3)$ and testing $\mathcal{O}(MN^2)$. Similarly, the time complexity for LOCI relies on the computation of the MDEF for each observation. When considering all possible radii r, it also takes $\mathcal{O}(N^3)$ for training and $\mathcal{O}(MN^2)$ for testing.

For kNN$_{global}$, kNN$_{local}$, LOF, and SOD the most expensive operation is the computation of the k nearest neighbors for all N observations. Without using any spatial index structures, this computation takes $\mathcal{O}(N^2)$. However, with the use of spatial index structures, such as k-d trees and R-trees (Friedman et al, 1977; Roussopoulos et al, 1995), the k nearest neighbors for all N observations can in practice often be determined much faster. For testing new instances, we have to compute the k nearest neighbors of these instances to all instances of the training set ($\mathcal{O}(MN)$). Similarly to k-nearest neighbor-based classifiers, the PW needs to compute the distance to all the observations in the training set in order to estimate the densities. Therefore, the PW time complexity is the same as that of the k-nearest neighbor-based methods.

The training phase of GLOSH consists of building the HDBSCAN* hierarchy. The two most expensive operations are the computation of the core distance, which involves computing the k nearest neighbors for all N observations ($\mathcal{O}(N^2)$), and the construction of the MST ($\mathcal{O}(N^2)$). Therefore, the overall time complexity for training is $\mathcal{O}(N^2)$. For testing, GLOSH has to compute the core distance for each testing point ($\mathcal{O}(MN)$), and find their respective closest objects in the MST ($\mathcal{O}(MN)$). In total, the testing time complexity is $\mathcal{O}(MN)$.

2 Detailed Results

In the following, we present the datasets and results in detail. Tables 2 and 3 show the characteristics of the datasets used in the experiments (number of features, the number of objects in each class, and the total number of objects in the datasets).

Tables 4-7, 8-11, and 12-15 display, respectively, ROC AUC, Adjusted-Prec@n, and MCC values for Type I experiments for each of the algorithms equipped with their best parameter value according to the different model selection methods (Cross-validation, SDS, Uniform Objects, Perturbation). Tables 16-19, 20-23, and 24-27 display, respectively, ROC AUC, Adjusted-Prec@n, and MCC values for Type II experiments for each of the algorithms equipped with their best parameter value according to the different model selection methods (Cross-validation, SDS, Uniform Objects, Perturbation). The highest achieved values for each dataset are shown in bold, and we visualize the distribution of the table values in Figures 4-23.

Tables 28 and 29 display, respectively, ROC AUC and AdjustedPrec@n values for the different approaches to guide the selection of the base models combined into the ensemble. The highest achieved values for each dataset are shown in bold.

Finally, Figure 3 show the complete critical difference diagrams (ROC AUC, AdjustedPrec@n, and MCC) with average ranks of the methods equipped with their best parameter value according to the different practical model selection methods (SDS, Uniform Objects, Perturbation).

Table 2: Description of the datasets used in the experiments.

	Features	Class Size											Total
Abalone	10	1407	1323	1447	-	-	-	-	-	-	-	-	4177
Arrhythmia	278	237	183	-	-	-	-	-	-	-	-	-	420
Artificial-characters	7	591	593	699	295	432	493	399	571	497	293	-	4863
Balancescale	4	288	49	288	-	-	-	-	-	-	-	-	625
Ball-bearing	32	913	3237	-	-	-	-	-	-	-	-	-	4150
Biomed	5	127	67	-	-	-	-	-	-	-	-	-	194
Breast	9	238	225	-	-	-	-	-	-	-	-	-	463
Cancer	33	151	47	-	-	-	-	-	-	-	-	-	198
Car-evaluation	6	384	69	1210	65	-	-	-	-	-	-	-	1728
Cardiotocography	34	1648	292	175	-	-	-	-	-	-	-	-	2115
Cellcycle	17	47	31	18	121	-	-	-	-	-	-	-	217
Cnae	856	112	120	118	116	116	114	117	120	119	-	-	1052
Colon	1908	22	40	-	-	-	-	-	-	-	-	-	62
Delft	64	[71, 376]	[229, 1124]	-	-	-	-	-	-	-	-	-	[300, 1500]
Dermatology	34	112	61	72	49	52	20	-	-	-	-	-	366
Diabetes	8	500	268	-	-	-	-	-	-	-	-	-	768
Ecoli	7	143	77	2	2	35	20	5	52	-	-	-	336
Flare	12	32	70	130	72	30	87	-	-	-	-	-	421
Glass	9	69	76	17	13	9	29	-	-	-	-	-	213
Hayes-roth	4	28	25	31	-	-	-	-	-	-	-	-	84
Heart	13	137	160	-	-	-	-	-	-	-	-	-	297
Hepatitis	19	123	32	-	-	-	-	-	-	-	-	-	155
Housing	13	458	48	-	-	-	-	-	-	-	-	-	506
Imports	25	71	88	-	-	-	-	-	-	-	-	-	159
Ionosphere	32	225	125	-	-	-	-	-	-	-	-	-	350
Iris	4	48	50	49	-	-	-	-	-	-	-	-	147
Led	7	7	10	16	6	9	9	8	8	7	5	-	85
Liver	6	142	199	-	-	-	-	-	-	-	-	-	341
Lung-cancer	56	9	13	10	-	-	-	-	-	-	-	-	32
Multiple-features	649	200	199	200	200	199	200	199	198	199	200	-	1994
Optdigits	62	554	554	562	571	557	572	568	558	558	566	-	5620
Pageblocks	10	4884	509	-	-	-	-	-	-	-	-	-	5393
Sat	36	1072	479	961	415	470	1038	-	-	-	-	-	4435
Seeds	7	70	70	70	-	-	-	-	-	-	-	-	210
Semeion	256	161	158	162	159	159	161	159	161	158	155	-	1593
Sonar	60	111	97	-	-	-	-	-	-	-	-	-	208
Soybean-small	21	10	10	10	17	-	-	-	-	-	-	-	47
Spectf	44	55	212	-	-	-	-	-	-	-	-	-	267
Survival	3	207	76	-	-	-	-	-	-	-	-	-	283
Synthetic	[20, 39]	[1613, 7529]	[41, 201]	-	-	-	-	-	-	-	-	-	[1654, 7730]
Systhetic-control	60	100	100	100	100	100	100	-	-	-	-	-	600
Texture	40	497	497	498	497	497	498	498	498	498	497	498	5473
User-knowledge	5	102	129	122	24	26	-	-	-	-	-	-	403
Vehicle	18	212	217	218	199	-	-	-	-	-	-	-	846
Vertebra-column	6	60	100	150	-	-	-	-	-	-	-	-	310
Vowels	10	48	48	48	48	48	48	48	48	48	48	48	528
Waveform	21	1657	1647	1696	-	-	-	-	-	-	-	-	5000
Wine	13	59	71	48	-	-	-	-	-	-	-	-	178
Yeastgalactose	80	83	15	93	14	-	-	-	-	-	-	-	205
Zoo	16	19	12	5	5	4	6	8	-	-	-	-	59

Table 3: Synthetic and Delft dataset variants. Both sets are aggregated and reported in the results as Synthetic and Delft respectively, as they are obtained from a single source.

	Features	Inliers	Outliers	Total
Delft1x3	64	71	229	300
Delft2x2	64	100	300	400
Delft3x2	64	137	463	600
Delft5x1	64	133	367	500
Delft5x3	64	376	1124	1500
Gauss20d4c	20	3364	89	3453
Gauss20d6c	20	4719	124	4843
Gauss22d5c	22	3978	100	4078
Gauss22d6c	22	4692	102	4794
Gauss22d9c	22	6841	194	7035
Gauss23d4c	23	3323	83	3406
Gauss23d9c	23	7182	177	7359
Gauss24d2c	24	1847	52	1899
Gauss24d3c	24	2580	69	2649
Gauss24d4c	24	3405	83	3488
Gauss24d7c	24	5653	141	5794
Gauss25d5c	25	3672	97	3769
Gauss25d9c	25	6982	179	7161
Gauss26d4c	26	3268	66	3334
Gauss27d5c	27	4113	84	4197
Gauss27d6c	27	4871	129	5000
Gauss28d4c	28	3377	98	3475
Gauss28d7c	28	5689	143	5832
Gauss29d3c	29	2579	59	2638
Gauss30d4c	30	3042	83	3125
Gauss31d4c	31	2750	74	2824
Gauss33d3c	33	2215	52	2267
Gauss33d5c	33	3630	92	3722
Gauss35d6c	35	4134	99	4233
Gauss36d8c	36	5998	135	6133
Gauss36d9c	36	6838	173	7011
Gauss37d3c	37	2409	51	2460
Gauss38d9c	38	7529	201	7730
Gauss39d2c	39	1613	41	1654
Gauss39d5c	39	4238	116	4354

Table 4: Detailed results of the methods with the parameters selected using Cross-validation over all datasets of the Type I experiments (single source-class inliers) with respect to ROC AUC. We visualize the distribution of these values in Figure 4(a).

ROC AUC	ABOD	Auto Enc.	GLOSH	GMM	iForest	KNN G.	SVDD	KNN L.	LOCI	LOF	LP	Parzen	SOD	DSVDD
Abalone	0.7	0.73	0.66	0.73	0.75	0.74	**0.76**	0.67	**0.76**	0.68	0.74	0.74	0.68	0.68
Arrhythmia	0.72	0.77	0.73	0.72	**0.79**	0.73	0.74	0.72	0.72	0.71	0.71	0.5	0.76	0.74
Artificial-characters	0.84	0.91	0.87	**0.92**	0.78	0.86	0.89	0.85	0.85	0.87	0.88	0.87	0.85	0.9
Balancescale	0.82	0.92	0.86	**0.95**	0.76	0.84	0.91	0.86	0.82	0.87	0.89	0.8	0.67	0.87
Ball-bearing	0.93	**1**	0.98	**1**	**1**	0.97	0.97	0.97	0.95	0.99	0.97	0.97	**1**	0.99
Biomed	0.62	0.67	0.68	0.73	0.75	**0.85**	0.81	0.84	0.73	0.74	0.68	0.79	0.76	0.82
Breast	0.97	0.84	**0.99**	0.9	0.98	0.98	0.98	0.94	**0.99**	**0.99**	0.93	**0.99**	0.96	0.91
Cancer	0.64	0.56	0.61	0.51	0.44	0.72	0.75	**0.83**	0.66	0.71	0.6	0.7	0.76	0.57
Car-evaluation	0.9	0.95	0.97	**0.99**	0.93	0.97	**0.99**	0.97	**0.99**	0.98	**0.99**	0.93	0.94	0.93
Cardiotocography	0.59	**0.99**	0.65	0.98	0.9	0.64	0.65	0.65	0.63	0.67	0.68	0.66	0.79	0.98
Cellcycle	0.79	0.66	0.8	0.65	0.66	**0.83**	0.68	0.75	0.81	**0.83**	0.72	0.79	0.6	0.78
Cnae	0.73	0.71	0.77	0.87	0.45	0.77	0.8	0.79	0.8	0.81	0.77	0.5	**0.91**	0.63
Colon	0.59	**0.73**	**0.73**	0.64	0.72	0.66	0.59	0.58	0.67	0.66	0.61	0.5	0.62	0.59
Delft	0.72	0.8	**0.96**	**0.96**	0.63	0.92	**0.96**	0.93	0.88	0.94	**0.96**	0.93	0.94	0.72
Dermatology	0.93	0.94	0.96	**0.99**	0.94	0.97	0.98	0.93	0.94	0.94	0.98	0.96	0.98	0.97
Diabetes	0.63	0.63	0.63	0.62	0.64	0.65	**0.67**	0.6	0.66	0.66	0.65	0.66	0.53	0.66
Ecoli	0.88	0.96	0.9	0.89	**0.97**	0.89	0.81	0.89	0.9	0.88	0.87	0.9	0.71	0.88
Flare	0.75	0.78	0.77	0.78	0.67	0.77	**0.82**	0.78	0.77	0.79	**0.82**	0.78	0.77	0.79
Glass	0.79	**0.84**	0.73	0.79	0.68	0.71	0.83	0.72	0.78	0.72	0.81	0.74	0.82	0.81
Hayes-roth	0.61	**0.79**	0.75	0.69	0.71	0.66	0.73	0.7	0.71	0.72	0.67	0.63	0.67	0.72
Heart	0.61	0.81	0.62	0.85	**0.9**	0.6	0.55	0.53	0.63	0.62	0.55	0.6	0.7	0.73
Hepatitis	0.41	0.65	0.36	**0.84**	0.83	0.39	0.51	0.19	0.49	0.37	0.49	0.39	0.65	0.7
Housing	0.7	0.81	0.64	0.87	**0.88**	0.69	0.72	0.61	0.63	0.65	0.68	0.7	0.78	0.83
Imports	0.67	0.82	0.82	0.84	0.81	0.54	0.86	0.55	0.75	0.87	**0.88**	0.87	0.75	0.82
Ionosphere	0.95	**0.99**	0.98	0.97	0.87	0.98	0.98	0.91	0.86	0.96	0.97	0.98	0.98	0.96
Iris	0.99	0.99	0.96	0.99	0.98	0.97	**1**	0.99	0.98	0.99	**1**	0.99	0.99	0.97
Led	0.81	0.78	0.82	0.91	0.84	0.92	**0.93**	0.86	0.79	0.82	**0.93**	**0.93**	0.84	0.71
Liver	0.6	**0.71**	0.59	0.6	0.6	0.6	0.54	0.53	0.59	0.6	0.52	0.6	0.46	0.54
Lung-cancer	0.75	**0.86**	0.76	0.78	0.67	0.65	0.71	0.78	0.83	0.82	0.71	0.71	0.76	0.6
Multiple-features	0.98	0.98	0.98	**0.99**	0.98	**0.99**	**0.99**	0.96	0.97	0.98	0.82	0.5	0.97	0.95
Optdigits	0.98	0.99	**1**	0.99	0.98	**1**	**1**	0.98	0.98	**1**	**1**	**1**	0.98	0.97
Pageblocks	0.52	0.92	0.83	**0.96**	0.92	0.63	0.86	0.9	0.91	0.94	0.8	0.68	0.76	0.74
Sat	0.95	0.91	0.95	0.87	**0.96**	0.95	**0.96**	0.91	0.95	0.93	0.95	**0.96**	0.95	0.91
Seeds	**0.99**	**0.99**	**0.99**	**0.99**	**0.99**	0.97	0.97	0.97	0.97	0.98	0.97	0.98	0.97	0.97
Semeion	0.94	0.93	**0.97**	**0.97**	0.95	**0.97**	0.96	0.91	0.94	0.96	**0.97**	0.96	**0.97**	0.78
Sonar	0.61	0.66	0.66	0.75	0.61	0.71	0.75	0.64	0.69	0.71	0.74	0.75	**0.81**	0.64
Soybean-small	**1**	**1**	**1**	**1**	0.98	**1**	**1**	0.93	**1**	**1**	**1**	**1**	**1**	**1**
Spectf	**0.85**	0.79	**0.85**	0.83	0.82	0.84	0.84	0.83	**0.85**	0.84	0.76	0.84	0.79	0.73
Survival	0.64	0.58	0.64	0.67	0.7	**0.77**	**0.77**	0.69	0.62	0.74	0.72	0.75	0.69	0.48
Systhetic-control	0.94	0.95	0.99	0.99	0.95	0.99	0.97	0.99	0.98	**1**	0.99	0.96	**1**	0.96
Texture	0.97	**1**	0.99	**1**	0.97	0.99	**1**	0.99	0.98	**1**	0.99	**1**	0.99	0.98
User-knowledge	0.91	0.94	0.91	0.94	0.86	0.91	0.93	0.87	0.89	0.88	0.92	0.93	0.94	**0.95**
Vehicle	0.75	0.86	0.74	**0.92**	0.78	0.75	0.79	0.74	0.74	0.74	0.79	0.76	0.85	0.86
Vertebra-column	0.89	0.9	0.87	0.86	0.87	0.89	**0.92**	0.9	0.9	0.9	0.89	0.9	0.9	0.83
Vowels	0.99	**1**	0.99	**1**	0.91	0.99	**1**	0.97	0.99	0.99	**1**	**1**	0.98	0.99
Waveform	0.89	0.87	0.88	0.87	**0.9**	0.88	**0.9**	0.84	0.89	0.87	0.89	**0.9**	0.81	0.73
Wine	0.84	0.88	0.82	0.92	0.91	0.82	0.85	0.84	0.82	0.82	0.79	0.82	0.88	**0.93**
Yeastgalactose	0.99	0.93	**1**	0.99	0.98	**1**	**1**	0.99	0.99	0.99	**1**	**1**	0.98	0.97
Zoo	0.95	**1**	0.89	**1**	**1**	0.87	**1**	0.81	0.82	0.87	**1**	**1**	**1**	0.84
	0.8	0.85	0.83	**0.87**	0.83	0.82	0.85	0.81	0.83	0.84	0.83	0.81	0.83	0.82

Table 5: Detailed results of the methods with the parameters selected using Self-Adaptive Data Shifting (SDS) over all datasets of the Type I experiments (single source-class inliers) with respect to ROC AUC. We visualize the distribution of these values in Figure 9(a).

ROC AUC	ABOD	Auto Enc.	GLOSH	GMM	iForest	KNN G.	SVDD	KNN L.	LOCI	LOF	LP	Parzen	SOD	DSVDD
Abalone	0.71	0.65	0.59	0.65	**0.75**	0.62	0.71	0.58	0.74	0.61	0.73	0.62	0.66	0.65
Arrhythmia	0.72	0.7	0.74	0.74	0.74	0.74	0.73	0.69	0.71	0.73	0.5	0.5	**0.75**	**0.75**
Artificial-characters	0.84	0.89	0.87	**0.91**	0.78	0.87	0.89	0.86	0.85	0.87	0.88	0.86	0.83	0.89
Balancescale	0.82	0.93	0.83	**0.95**	0.75	0.82	0.91	0.7	0.74	0.86	0.87	0.8	0.64	0.83
Ball-bearing	0.93	**1**	0.97	**1**	**1**	0.97	0.97	0.97	0.95	0.98	0.97	0.97	**1**	0.99
Biomed	0.6	0.7	0.54	0.71	0.72	0.58	0.73	0.64	0.69	0.62	0.66	**0.79**	0.51	0.77
Breast	0.96	0.81	0.89	0.93	**0.98**	0.79	0.95	0.92	**0.98**	0.89	0.93	0.88	0.96	0.86
Cancer	0.64	0.55	0.68	**0.72**	0.59	0.66	0.71	0.68	0.66	0.68	0.67	0.66	0.63	0.53
Car-evaluation	0.9	0.94	0.87	**0.99**	0.84	0.94	**0.99**	0.94	0.97	**0.99**	**0.99**	0.93	0.92	0.93
Cardiotocography	0.58	**0.98**	0.66	0.97	0.86	0.64	0.6	0.58	0.64	0.59	0.68	0.56	0.69	0.97
Cellcycle	**0.78**	0.71	0.77	0.62	0.67	0.74	0.64	0.54	0.75	0.64	0.62	0.66	0.6	0.68
Cnae	0.74	0.68	0.74	**0.86**	0.4	0.73	0.76	0.7	0.74	0.73	0.74	0.5	0.76	0.49
Colon	0.59	0.44	**0.69**	0.66	0.61	0.66	0.59	0.61	0.66	0.66	0.5	0.5	0.59	0.49
Delft	0.7	0.77	0.92	0.95	0.67	0.92	**0.96**	0.93	0.87	0.94	0.95	0.86	0.9	0.65
Dermatology	0.91	0.91	0.96	**0.99**	0.84	0.97	0.98	0.92	0.94	0.94	0.98	0.97	**0.99**	0.87
Diabetes	0.64	0.55	**0.65**	0.6	0.64	0.64	0.62	0.57	0.63	0.64	0.64	0.63	0.6	0.58
Ecoli	0.88	0.81	0.89	0.74	**0.96**	0.88	0.84	0.9	0.9	0.9	0.86	0.87	0.6	0.87
Flare	0.76	0.75	0.7	**0.79**	0.72	0.74	0.78	0.69	0.76	0.72	0.76	0.75	**0.79**	0.74
Glass	0.78	0.76	0.71	0.8	0.62	0.58	0.8	0.61	0.73	0.65	0.81	0.71	**0.84**	0.71
Hayes-roth	0.59	0.53	0.65	0.63	0.61	0.69	0.67	0.71	0.68	0.69	**0.74**	0.63	0.68	0.55
Heart	0.61	0.62	0.53	0.77	**0.85**	0.6	0.55	0.59	0.53	0.57	0.54	0.61	0.64	0.68
Hepatitis	0.44	0.65	0.63	**0.88**	**0.88**	0.45	0.38	0.55	0.56	0.56	0.33	0.39	0.72	0.75
Housing	0.69	0.75	0.55	**0.88**	0.84	0.66	0.72	0.56	0.65	0.58	0.66	0.66	0.73	0.76
Imports	0.66	0.56	0.58	0.68	0.63	0.61	0.74	0.56	0.73	0.59	0.71	0.68	**0.76**	0.73
Ionosphere	0.94	0.97	**0.98**	0.97	0.93	**0.98**	**0.98**	0.9	0.85	0.96	0.97	**0.98**	0.96	0.96
Iris	0.99	**1**	0.98	0.98	0.98	0.98	**1**	0.99	0.98	0.98	**1**	0.99	0.98	0.94
Led	0.93	0.78	0.85	0.93	0.79	0.88	0.94	0.85	0.71	0.81	**0.96**	0.93	0.74	0.6
Liver	0.61	0.56	0.57	0.58	0.63	0.61	0.58	0.46	**0.65**	0.63	0.57	0.61	0.58	0.59
Lung-cancer	0.75	**0.85**	0.73	0.76	0.57	0.75	0.75	0.72	0.74	0.75	0.71	0.75	0.68	0.48
Multiple-features	0.97	0.94	0.97	**0.99**	0.98	**0.99**	0.98	0.94	0.97	0.96	0.55	0.5	0.97	0.91
Optdigits	0.98	0.99	**1**	0.99	0.98	**1**	**1**	0.98	0.98	**1**	**1**	**1**	0.98	0.96
Pageblocks	0.52	0.9	0.74	0.84	**0.92**	0.63	0.8	0.83	0.78	**0.92**	0.77	0.62	0.71	0.74
Sat	0.95	0.87	0.94	0.85	**0.96**	0.94	0.95	0.89	0.93	0.91	0.95	0.95	0.95	0.9
Seeds	0.98	**0.99**	0.93	**0.99**	**0.99**	0.98	0.98	0.94	0.97	0.96	0.98	0.98	0.98	0.95
Semeion	0.94	0.88	**0.97**	**0.97**	0.86	**0.97**	0.96	0.91	0.94	0.95	**0.97**	0.96	0.96	0.61
Sonar	0.6	0.63	0.65	0.74	0.66	0.66	0.72	0.57	0.64	0.6	0.7	0.63	**0.75**	0.64
Soybean-small	**1**	0.88	**1**	**1**	**1**	**1**	**1**	0.93	**1**	**1**	**1**	**1**	0.88	0.82
Spectf	0.85	0.71	0.84	0.7	0.82	0.83	0.84	0.81	**0.86**	0.8	0.57	0.83	0.8	0.79
Survival	0.64	0.54	0.63	0.69	0.7	0.61	**0.76**	0.69	0.63	0.71	0.72	0.65	0.63	0.48
Systhetic-control	0.94	0.91	0.99	0.9	0.96	0.99	0.97	0.99	0.97	**1**	0.5	0.96	0.99	0.85
Texture	0.97	**1**	0.99	**1**	0.97	0.99	**1**	0.99	0.98	**1**	0.99	0.99	0.99	0.98
User-knowledge	0.91	0.93	0.9	0.94	0.87	0.93	0.94	0.83	0.9	0.9	0.93	0.93	**0.95**	0.92
Vehicle	0.74	0.85	0.68	**0.92**	0.79	0.76	0.78	0.73	0.77	0.72	0.8	0.74	0.85	0.8
Vertebra-column	0.89	0.89	0.89	**0.92**	0.88	0.9	0.91	0.85	0.87	0.88	0.9	0.89	0.84	0.75
Vowels	0.99	0.98	0.98	0.99	0.92	0.99	**1**	0.95	0.98	0.98	**1**	0.99	0.99	0.96
Waveform	0.89	0.86	0.87	0.86	**0.9**	0.87	0.88	0.83	0.89	0.85	0.88	**0.9**	0.63	0.7
Wine	0.85	0.88	0.75	**0.96**	0.9	0.82	0.73	0.8	0.81	0.81	0.8	0.83	0.77	0.8
Yeastgalactose	0.99	0.98	**1**	0.98	0.91	0.99	**1**	0.96	0.99	0.99	**1**	**1**	0.99	0.88
Zoo	0.87	**1**	0.98	**1**	0.96	0.99	**1**	0.92	0.89	0.84	**1**	**1**	0.91	0.74
	0.8	0.8	0.8	**0.85**	0.81	0.81	0.83	0.78	0.81	0.81	0.79	0.79	0.8	0.76

Table 6: Detailed results of the methods with the parameters selected using Uniform Objects over all datasets of the Type I experiments (single source-class inliers) with respect to ROC AUC. We visualize the distribution of these values in Figure 9(b).

ROC AUC	ABOD	Auto Enc.	GLOSH	GMM	iForest	KNN G.	SVDD	KNN L.	LOCI	LOF	LP	Parzen	SOD	DSVDD
Abalone	0.71	0.65	0.59	0.66	**0.75**	0.63	0.72	0.61	0.74	0.61	0.61	0.73	0.65	0.66
Arrhythmia	0.72	0.7	0.71	0.74	0.73	0.73	**0.75**	0.62	0.73	0.73	0.5	0.5	0.71	0.62
Artificial-characters	0.84	0.89	0.87	**0.92**	0.78	0.86	0.78	0.85	0.84	0.87	0.88	0.83	0.84	0.88
Balancescale	0.82	0.88	0.86	**0.94**	0.8	0.85	0.87	0.86	0.78	0.85	0.89	0.77	0.64	0.85
Ball-bearing	0.93	0.97	0.97	**1**	**1**	0.97	0.73	0.97	0.93	0.98	0.97	0.91	**1**	0.99
Biomed	0.6	0.7	0.59	0.72	0.7	0.58	0.67	**0.78**	0.69	0.64	0.7	0.7	0.6	0.7
Breast	0.96	0.82	0.95	0.8	0.98	0.94	0.96	0.89	**0.99**	0.83	0.93	0.98	0.95	0.79
Cancer	0.64	0.54	0.68	**0.72**	0.6	0.62	0.6	0.65	0.66	0.68	0.63	0.67	0.6	0.54
Car-evaluation	0.9	0.88	0.96	**0.99**	0.94	0.98	0.91	0.97	**0.99**	0.93	0.88	0.93	0.91	0.93
Cardiotocography	0.58	0.97	0.58	**0.98**	0.9	0.58	0.51	0.59	0.58	0.64	0.68	0.53	0.69	0.87
Cellcycle	0.78	0.67	**0.82**	0.66	0.61	0.81	0.7	0.77	0.76	0.78	0.64	0.78	0.6	0.7
Cnae	0.74	0.69	0.77	0.87	0.38	0.76	0.78	0.72	0.74	0.77	0.73	0.5	**0.88**	0.5
Colon	0.59	0.61	0.61	0.61	**0.7**	0.61	0.58	0.55	0.66	0.69	0.5	0.5	0.59	0.57
Delft	0.7	0.75	0.78	**0.96**	0.64	0.84	0.78	0.88	0.87	0.9	0.91	0.64	0.89	0.56
Dermatology	0.91	0.91	0.95	**0.99**	0.94	0.96	0.94	0.84	0.94	0.88	0.98	0.82	0.96	0.81
Diabetes	**0.64**	0.53	0.62	0.61	**0.64**	**0.64**	0.5	0.57	0.63	**0.64**	0.62	0.61	0.58	0.53
Ecoli	0.88	0.71	0.88	0.73	**0.95**	0.88	0.86	0.9	0.87	0.89	0.87	0.89	0.59	0.9
Flare	0.76	0.73	0.71	**0.79**	0.67	0.73	**0.79**	0.71	0.74	0.74	**0.79**	0.76	**0.79**	0.73
Glass	0.78	0.77	0.71	0.77	0.66	0.69	0.7	0.67	0.69	0.72	0.79	0.76	**0.82**	0.61
Hayes-roth	0.59	0.7	0.66	0.51	0.6	0.6	0.52	0.69	**0.73**	0.71	0.61	0.6	0.72	0.61
Heart	0.61	0.57	0.56	0.75	**0.85**	0.6	0.59	0.56	0.54	0.46	0.54	0.6	0.71	0.67
Hepatitis	0.44	0.69	0.32	0.88	**0.89**	0.45	0.55	0.54	0.41	0.39	0.35	0.4	0.67	0.6
Housing	0.69	0.78	0.62	**0.85**	0.8	0.68	0.6	0.56	0.71	0.58	0.66	0.6	0.66	0.72
Imports	0.66	0.56	0.61	0.69	0.63	0.69	0.59	0.56	0.73	0.56	0.63	0.63	**0.82**	0.77
Ionosphere	0.94	0.97	0.96	**0.98**	0.93	0.94	0.88	0.9	0.81	0.9	0.9	0.94	0.97	0.95
Iris	0.99	**1**	0.98	0.99	0.98	0.98	0.99	0.98	0.98	0.98	0.99	0.98	0.98	0.92
Led	0.93	0.7	0.84	0.91	0.77	0.88	**0.95**	0.83	0.71	0.86	0.93	0.93	0.78	0.65
Liver	0.61	0.55	0.63	0.6	0.63	0.61	0.43	0.56	0.63	0.6	0.57	0.6	**0.66**	0.57
Lung-cancer	0.75	**0.85**	0.73	0.72	0.64	0.75	0.75	0.64	0.74	0.75	0.71	0.65	0.68	0.55
Multiple-features	0.97	0.96	0.93	**0.99**	0.98	0.98	0.79	0.94	0.97	0.92	0.5	0.5	0.97	0.73
Optdigits	0.98	0.99	**1**	0.99	0.97	**1**	0.96	0.98	0.98	**1**	0.92	0.99	0.98	0.94
Pageblocks	0.52	0.78	0.81	**0.96**	0.91	0.63	0.76	0.8	0.91	0.93	0.78	0.64	0.75	0.67
Sat	0.95	0.84	0.93	0.85	**0.96**	0.94	0.87	0.85	0.94	0.9	0.95	0.95	0.95	0.89
Seeds	0.98	0.98	0.93	**0.99**	0.98	0.97	0.97	0.91	0.97	0.93	0.98	0.98	0.98	0.86
Semeion	0.94	0.88	0.96	**0.97**	0.94	0.96	0.95	0.92	0.94	0.96	0.96	0.95	0.96	0.66
Sonar	0.6	0.63	0.55	0.64	0.63	0.64	0.56	0.59	0.59	0.56	0.49	0.57	**0.81**	0.51
Soybean-small	**1**	0.88	**1**	**1**	0.96	**1**	**1**	0.96	**1**	**1**	**1**	**1**	**1**	0.82
Spectf	0.85	0.82	0.84	0.83	0.82	0.84	**0.86**	0.81	**0.86**	0.84	0.57	0.84	0.79	0.76
Survival	0.64	0.5	0.63	0.63	0.69	0.62	0.59	0.6	0.62	**0.73**	0.68	0.65	0.63	0.5
Systhetic-control	0.94	0.91	**0.99**	**0.99**	0.96	0.98	0.92	**0.99**	0.98	**0.99**	0.5	0.96	**0.99**	0.78
Texture	0.97	0.99	0.99	0.99	0.96	0.98	0.96	0.99	0.98	**1**	0.95	0.96	0.99	0.97
User-knowledge	0.91	**0.96**	0.91	0.94	0.89	0.91	0.88	0.86	0.9	0.9	0.85	0.92	**0.96**	0.94
Vehicle	0.74	0.74	0.72	**0.92**	0.79	0.74	0.56	0.69	0.76	0.69	0.78	0.66	0.83	0.73
Vertebra-column	0.89	0.84	0.88	0.85	0.82	0.9	**0.92**	0.82	0.89	0.89	0.9	0.89	0.81	0.75
Vowels	**0.99**	0.96	**0.99**	**0.99**	0.91	**0.99**	0.82	0.97	**0.99**	0.98	0.93	0.96	0.98	0.95
Waveform	0.89	0.86	0.88	0.88	**0.9**	0.88	0.89	0.84	0.89	0.87	0.86	**0.9**	0.75	0.71
Wine	0.85	0.87	0.82	**0.93**	0.9	0.81	0.83	0.76	0.81	0.8	0.8	0.83	0.72	0.73
Yeastgalactose	0.99	0.98	0.98	0.96	0.97	**1**	**1**	0.93	0.99	0.99	**1**	**1**	0.98	0.83
Zoo	0.87	**1**	0.94	**1**	0.94	0.99	**1**	0.92	0.93	0.84	**1**	0.98	0.94	0.72
	0.8	0.8	0.8	**0.85**	0.82	0.81	0.78	0.78	0.81	0.8	0.77	0.77	0.81	0.74

Table 7: Detailed results of the methods with the parameters selected using Perturbation over all datasets of the Type I experiments (single source-class inliers) with respect to ROC AUC. We visualize the distribution of these values in Figure 9(c).

ROC AUC	ABOD	Auto Enc.	GLOSH	GMM	iForest	KNN G.	SVDD	KNN L.	LOCI	LOF	LP	Parzen	SOD	DSVDD
Abalone	0.71	0.64	0.64	0.67	**0.75**	0.65	0.63	0.61	0.74	0.61	0.61	0.73	0.64	0.67
Arrhythmia	0.72	0.7	0.74	0.74	0.74	0.73	0.75	0.69	0.73	0.72	0.5	0.5	**0.78**	0.66
Artificial-characters	0.84	0.9	0.87	**0.92**	0.79	0.86	0.82	0.86	0.85	0.87	0.88	0.86	0.83	0.89
Balancescale	0.82	0.86	0.85	**0.93**	0.79	0.83	0.86	0.85	0.82	0.86	0.85	0.8	0.64	0.82
Ball-bearing	0.93	0.99	0.97	**1**	**1**	0.97	0.94	0.97	0.95	0.99	0.97	0.94	**1**	0.99
Biomed	0.6	0.7	0.58	**0.71**	0.7	0.58	0.7	0.61	0.62	0.64	0.7	0.66	0.6	0.68
Breast	0.96	0.82	0.76	0.77	**0.98**	0.81	0.97	0.73	**0.98**	0.7	0.93	0.89	0.94	0.82
Cancer	0.64	0.54	0.68	**0.72**	0.59	0.66	0.58	0.65	0.69	0.68	0.63	0.66	0.62	0.54
Car-evaluation	0.9	0.9	0.97	**0.99**	0.94	0.97	0.91	0.97	**0.99**	0.93	0.88	0.93	0.92	0.93
Cardiotocography	0.58	0.97	0.66	**0.98**	0.88	0.64	0.61	0.65	0.63	0.63	0.68	0.54	0.74	0.97
Cellcycle	**0.78**	0.67	0.64	0.65	0.58	0.6	0.69	0.65	0.73	0.7	0.64	**0.78**	0.64	0.74
Cnae	0.74	0.67	0.72	0.87	0.38	0.71	0.78	0.69	0.76	0.74	0.72	0.5	**0.88**	0.5
Colon	0.59	**0.75**	0.7	0.61	**0.75**	0.59	0.58	0.7	0.66	0.63	0.5	0.5	0.64	0.39
Delft	0.7	0.84	0.94	**0.96**	0.64	0.91	0.85	0.92	0.89	0.92	0.91	0.86	0.86	0.64
Dermatology	0.91	0.91	0.95	**0.99**	0.88	0.96	0.95	0.9	0.94	0.93	0.97	0.93	**0.99**	0.88
Diabetes	0.64	0.59	**0.66**	0.58	0.65	0.64	0.54	0.59	0.64	0.65	0.62	0.63	0.6	0.56
Ecoli	0.88	0.81	0.88	0.74	**0.97**	0.88	0.84	0.9	0.85	0.6	0.86	0.89	0.59	0.86
Flare	0.76	0.75	0.71	0.78	0.69	0.78	0.8	0.74	0.75	0.72	**0.81**	0.77	0.77	0.71
Glass	0.78	0.75	0.7	0.79	0.6	0.7	0.71	0.62	0.69	0.64	0.8	0.8	**0.83**	0.72
Hayes-roth	0.59	0.7	0.63	**0.77**	0.54	0.53	0.53	0.63	0.67	0.69	0.51	0.57	0.58	0.61
Heart	0.61	0.62	0.56	0.75	**0.85**	0.61	0.63	0.55	0.57	0.59	0.54	0.61	0.73	0.64
Hepatitis	0.44	0.65	0.63	0.88	**0.89**	0.45	0.4	0.54	0.41	0.35	0.35	0.39	0.68	0.64
Housing	0.69	0.72	0.61	**0.88**	0.86	0.66	0.61	0.57	0.71	0.66	0.66	0.66	0.66	0.75
Imports	0.66	0.61	**0.82**	0.69	0.67	0.55	0.59	0.74	0.64	0.6	0.63	0.76	0.79	0.79
Ionosphere	0.94	**0.99**	0.96	0.98	0.9	0.97	0.9	0.89	0.85	0.92	0.9	0.94	0.97	0.95
Iris	0.99	**1**	0.98	0.99	0.96	0.99	0.99	0.98	0.98	0.98	0.99	0.99	0.98	0.92
Led	0.93	0.71	0.73	0.9	0.76	0.87	**0.95**	0.83	0.67	0.87	0.93	0.93	0.82	0.65
Liver	0.61	0.62	0.57	0.58	0.63	0.61	0.47	0.56	**0.65**	0.63	0.57	0.61	0.59	0.57
Lung-cancer	0.75	**0.82**	0.63	0.72	0.74	0.67	0.75	0.72	0.74	0.75	0.71	0.65	0.57	0.58
Multiple-features	0.97	0.96	0.96	**0.99**	0.96	**0.99**	0.95	0.94	0.97	0.97	0.5	0.5	0.97	0.87
Optdigits	0.98	0.99	**1**	0.99	0.97	**1**	0.99	0.98	0.98	**1**	0.92	**1**	0.98	0.95
Pageblocks	0.52	0.88	0.65	0.88	0.92	0.62	0.81	0.91	0.91	**0.94**	0.78	0.64	0.72	0.75
Sat	**0.95**	0.86	0.93	0.85	**0.95**	0.94	0.89	0.9	0.93	0.9	**0.95**	**0.95**	**0.95**	0.89
Seeds	0.98	0.98	0.92	**0.99**	0.98	0.98	0.96	0.94	0.97	0.93	0.98	0.98	0.98	0.93
Semeion	0.94	0.9	0.96	**0.97**	0.9	0.95	0.95	0.9	0.93	0.96	0.96	0.86	0.93	0.59
Sonar	0.6	0.65	0.65	0.64	0.67	0.66	0.58	0.57	0.58	0.61	0.58	0.63	**0.75**	0.57
Soybean-small	**1**	**1**	0.95	**1**	0.96	0.95	**1**	0.96	**1**	**1**	**1**	**1**	0.83	0.88
Spectf	**0.85**	0.82	**0.85**	0.83	0.82	0.83	**0.85**	0.82	0.84	0.83	0.57	0.84	0.78	0.66
Survival	0.64	0.5	0.63	0.67	0.72	0.67	0.65	0.65	0.63	**0.73**	0.69	0.65	0.56	0.52
Systhetic-control	0.94	0.89	**0.99**	**0.99**	0.94	0.98	0.93	**0.99**	0.98	**0.99**	0.5	0.96	0.98	0.78
Texture	0.97	0.99	0.99	0.99	0.97	0.99	0.96	0.99	0.98	**1**	0.96	0.97	0.99	0.97
User-knowledge	0.91	**0.95**	0.88	0.94	0.81	0.88	0.88	0.84	0.88	0.87	0.86	0.92	**0.95**	0.94
Vehicle	0.74	0.74	0.7	**0.92**	0.79	0.73	0.61	0.71	0.77	0.7	0.78	0.71	0.84	0.81
Vertebra-column	0.89	0.84	0.88	0.85	0.86	0.9	**0.92**	0.86	0.89	0.89	0.9	0.89	0.85	0.74
Vowels	**0.99**	0.97	**0.99**	**0.99**	0.9	**0.99**	0.89	0.98	0.97	0.98	0.97	**0.99**	0.95	0.97
Waveform	0.89	0.86	0.88	0.87	0.89	0.88	0.88	0.84	0.88	0.87	0.86	**0.9**	0.75	0.71
Wine	0.85	0.87	0.83	**0.93**	0.91	0.84	0.71	0.77	0.82	0.81	0.8	0.82	0.85	0.85
Yeastgalactose	0.99	0.96	**1**	0.96	0.87	**1**	**1**	0.93	0.98	0.99	**1**	**1**	0.98	0.84
Zoo	0.87	0.98	0.89	**1**	0.96	**1**	**1**	0.95	0.91	0.84	**1**	0.98	0.91	0.78
	0.8	0.81	0.8	**0.85**	0.81	0.8	0.79	0.79	0.81	0.8	0.77	0.79	0.8	0.76

Table 8: Detailed results of the methods with the parameters selected using Cross-validation over all datasets of the Type I experiments (single source-class inliers) with respect to AdjustedPrec@n. We visualize the distribution of these values in Figure 4(b).

AdjustedPrec@n	ABOD	Auto Enc.	GLOSH	GMM	iForest	KNN G.	SVDD	KNN L.	LOCI	LOF	LP	Parzen	SOD	DSVDD
Abalone	0.29	0.3	0.21	0.32	0.32	0.32	**0.36**	0.18	0.35	0.25	0.34	0.31	0.24	0.26
Arrhythmia	0.31	0.41	0.31	0.26	**0.56**	0.31	0.36	0.26	0.31	0.31	0.31	-0.03	0.46	0.32
Artificial-characters	0.31	0.37	0.33	**0.39**	0.25	0.31	**0.39**	0.28	0.36	0.32	0.37	0.32	0.21	0.38
Balancescale	0.35	0.59	0.47	**0.83**	0.4	0.49	0.6	0.5	0.42	0.51	0.54	0.4	0.28	0.51
Ball-bearing	0.75	0.95	0.87	**0.97**	0.96	0.86	0.87	0.8	0.74	0.89	0.86	0.86	0.95	0.9
Biomed	0.24	0.3	0.3	0.36	0.36	**0.59**	0.53	0.53	0.47	0.36	0.3	0.53	0.47	0.57
Breast	0.83	0.61	0.89	0.67	**0.93**	0.89	0.89	0.78	0.89	0.91	0.78	0.89	0.8	0.7
Cancer	0.21	0.13	0.13	-0.01	-0.01	0.21	0.28	**0.42**	0.13	0.21	0.28	0.21	0.35	0.12
Car-evaluation	0.58	0.78	0.69	0.87	0.61	0.65	**0.9**	0.65	0.82	0.66	0.86	0.63	0.48	0.63
Cardiotocography	0.13	0.84	0.19	**0.91**	0.6	0.14	0.14	0.16	0.15	0.2	0.24	0.2	0.36	0.86
Cellcycle	0.45	0.3	0.38	0.43	0.38	0.4	0.36	0.47	0.43	**0.5**	0.36	0.4	0.14	0.42
Cnae	0.34	0.42	0.37	0.6	0.03	0.39	0.44	0.45	0.46	0.41	0.39	-0.04	**0.66**	0.18
Colon	-0.12	**0.62**	0.25	0.06	0.06	0.06	-0.12	0.25	0.06	0.06	0.06	0.25	0.06	0.18
Delft	0.28	0.48	**0.75**	0.74	0.14	0.61	0.74	0.68	0.64	0.66	0.74	0.66	0.7	0.28
Dermatology	0.7	0.66	0.73	**0.9**	0.72	0.77	0.86	0.71	0.79	0.72	0.83	0.77	0.86	0.81
Diabetes	0.22	0.16	0.22	0.09	0.18	0.22	0.23	0.15	**0.26**	0.25	0.25	0.23	0.08	0.21
Ecoli	0.65	**0.76**	**0.76**	**0.76**	**0.76**	**0.76**	0.29	**0.76**	**0.76**	**0.76**	**0.76**	**0.76**	0.29	0.46
Flare	0.34	0.41	**0.43**	**0.43**	0.16	0.41	0.4	0.35	0.41	**0.43**	0.39	0.38	0.39	0.41
Glass	0.31	0.42	0.45	0.33	0.27	0.43	0.43	0.33	0.31	0.33	**0.47**	0.33	0.36	0.37
Hayes-roth	0.06	0.35	**0.53**	0.15	0.14	0.24	0.25	0.25	0.35	0.43	0.15	0.06	0.34	0.24
Heart	0.15	0.45	0.15	0.62	**0.66**	0.15	0.11	-0.02	0.18	0.18	0.11	0.15	0.28	0.32
Hepatitis	-0.25	0.17	-0.25	0.38	**0.58**	-0.25	0.17	-0.25	-0.25	-0.25	-0.25	-0.25	-0.04	0.12
Housing	0.15	0.33	0.15	0.33	**0.57**	0.27	0.15	0.15	0.27	0.08	0.21	0.08	0.15	0.34
Imports	0.22	0.48	0.48	0.61	0.48	0.09	0.61	-0.04	0.35	**0.74**	0.61	0.61	0.35	0.56
Ionosphere	0.69	**0.88**	0.81	0.81	0.5	0.81	0.81	0.63	0.5	0.81	0.81	0.81	**0.88**	0.8
Iris	**0.95**	0.89	0.79	0.89	0.84	0.84	**0.95**	0.89	0.84	0.89	**0.95**	0.84	**0.95**	0.82
Led	0.41	0.17	-0.05	0.22	0.48	0.54	0.65	0.37	0.16	0.21	**0.69**	**0.69**	0.01	0.13
Liver	0.14	**0.33**	0.14	0.14	0.2	0.2	0.08	0.02	0.2	0.2	0.02	0.2	0.02	0.07
Lung-cancer	0.47	0.47	**0.75**	0.47	0.47	0.47	0.47	0.47	0.47	0.06	0.47	0.47	0.47	0.07
Multiple-features	0.79	0.79	0.86	**0.93**	0.86	0.88	0.88	0.7	0.81	0.86	0.62	0.02	0.76	0.66
Optdigits	0.85	0.92	0.94	0.89	0.83	0.94	0.94	0.79	0.88	0.94	**0.95**	0.93	0.82	0.75
Pageblocks	0.03	0.63	0.32	**0.66**	0.56	0.18	0.24	0.57	0.52	**0.66**	0.18	0.01	0.26	0.5
Sat	0.74	0.63	0.75	0.51	0.75	0.76	0.75	0.59	0.72	0.68	0.74	**0.77**	0.69	0.58
Seeds	0.89	0.89	0.86	**0.93**	**0.93**	0.79	0.82	0.82	0.86	0.79	0.82	0.82	0.82	0.83
Semeion	0.71	0.62	0.79	0.8	0.69	0.79	0.78	0.59	0.77	0.77	**0.81**	0.74	0.8	0.28
Sonar	0.07	0.36	0.26	0.26	0.07	0.26	0.41	0.22	0.31	0.36	0.36	0.41	**0.51**	0.23
Soybean-small	**1**	**1**	**1**	**1**	0.84	**1**	**1**	0.68	**1**	**1**	**1**	**1**	**1**	0.97
Spectf	0.43	0.43	0.43	0.31	0.2	**0.54**	0.43	0.43	**0.54**	0.43	0.31	0.43	0.08	0.17
Survival	0.13	0.13	0.18	0.23	0.18	**0.36**	**0.36**	0.32	0.18	0.27	**0.36**	**0.36**	0.27	-0.03
Systhetic-control	0.63	0.66	0.92	0.88	0.65	0.88	0.8	0.9	0.84	**0.96**	0.89	0.76	0.92	0.71
Texture	0.83	**0.96**	0.93	0.95	0.8	0.92	0.93	0.86	0.87	0.94	0.9	0.94	0.92	0.81
User-knowledge	0.47	**0.67**	0.43	0.55	0.48	0.51	0.62	0.43	0.51	0.36	0.57	0.52	0.62	0.64
Vehicle	0.31	0.52	0.41	**0.69**	0.39	0.42	0.43	0.33	0.39	0.34	0.41	0.39	0.56	0.5
Vertebra-column	0.56	0.55	0.44	0.49	0.45	0.54	0.59	0.57	0.62	0.55	0.52	0.58	**0.65**	0.48
Vowels	0.88	0.93	0.9	**0.98**	0.54	0.92	0.97	0.8	0.86	0.85	0.93	0.96	0.85	0.83
Waveform	0.59	0.51	0.56	0.52	0.6	0.56	**0.61**	0.47	0.59	0.52	0.56	**0.61**	0.43	0.26
Wine	0.51	0.67	0.47	**0.71**	0.67	0.43	0.52	0.52	0.43	0.47	0.38	0.47	**0.71**	0.69
Yeastgalactose	0.88	0.79	**0.97**	0.88	0.88	**0.97**	**0.97**	0.86	0.88	0.88	**0.97**	**0.97**	0.88	0.81
Zoo	0.82	**1**	0.63	**1**	**1**	0.63	**1**	0.56	0.63	0.56	**1**	**1**	**1**	0.5
	0.45	0.57	0.52	**0.59**	0.51	0.52	0.56	0.47	0.51	0.52	0.53	0.5	0.51	0.47

Table 9: Detailed results of the methods with the parameters selected using Self-Adaptive Data Shifting (SDS) over all datasets of the Type I experiments (single source-class inliers) with respect to AdjustedPrec@n. We visualize the distribution of these values in Figure 10(a).

AdjustedPrec@n	ABOD	Auto Enc.	GLOSH	GMM	iForest	KNN G.	SVDD	KNN L.	LOCI	LOF	LP	Parzen	SOD	DSVDD
Abalone	0.31	0.19	0.09	0.18	**0.34**	0.15	0.31	0.05	0.3	0.11	0.3	0.15	0.2	0.2
Arrhythmia	0.31	0.26	**0.36**	**0.36**	0.31	**0.36**	0.31	0.26	0.31	**0.36**	-0.03	-0.03	**0.36**	0.34
Artificial-characters	0.31	0.36	0.33	**0.39**	0.24	0.32	0.38	0.32	0.35	0.33	0.36	0.31	0.18	0.36
Balancescale	0.35	**0.6**	0.4	**0.6**	0.38	0.39	0.56	0.39	0.39	0.52	0.54	0.42	0.29	0.44
Ball-bearing	0.75	0.96	0.86	**0.97**	0.96	0.86	0.85	0.82	0.75	0.87	0.87	0.86	0.96	0.9
Biomed	0.24	0.42	0.06	0.36	0.36	0.18	0.47	0.3	0.3	0.24	0.36	**0.53**	0.01	0.46
Breast	0.83	0.52	0.63	0.83	**0.87**	0.48	0.83	0.76	0.8	0.67	0.76	0.63	0.85	0.64
Cancer	0.13	**0.28**	**0.28**	0.13	-0.01	0.21	0.13	0.21	**0.28**	0.13	**0.28**	0.21	0.21	0.03
Car-evaluation	0.58	0.72	0.28	0.84	0.38	0.59	0.87	0.6	0.68	0.83	**0.89**	0.62	0.51	0.53
Cardiotocography	0.13	**0.84**	0.17	**0.84**	0.54	0.16	0.12	0.13	0.17	0.14	0.24	0.09	0.22	0.8
Cellcycle	**0.45**	0.29	0.36	0.43	0.33	0.29	0.36	0.07	0.38	0.19	0.33	0.36	0.33	0.29
Cnae	0.33	0.27	0.31	**0.58**	0.05	0.31	0.33	0.29	0.3	0.24	0.32	-0.04	0.28	0.02
Colon	-0.12	-0.12	**0.25**	0.06	0.06	0.06	-0.12	0.06	0.06	0.06	**0.25**	**0.25**	-0.12	0.03
Delft	0.24	0.44	0.67	0.73	0.15	0.63	**0.75**	0.69	0.63	0.66	0.73	0.55	0.62	0.16
Dermatology	0.7	0.67	0.8	**0.91**	0.41	0.77	0.86	0.64	0.77	0.73	0.82	0.77	0.88	0.48
Diabetes	0.22	0.03	0.21	0.11	0.21	0.22	0.23	0.09	0.16	0.23	**0.28**	0.21	0.15	0.11
Ecoli	**0.76**	0.29	**0.76**	0.17	**0.76**	0.65	0.41	0.65	**0.76**	**0.76**	0.65	**0.76**	0.17	0.41
Flare	0.37	0.37	0.38	**0.47**	0.19	0.36	0.45	0.37	0.42	0.36	0.46	0.37	0.42	0.26
Glass	0.31	0.31	0.29	**0.42**	0.15	0.11	0.27	0.23	0.24	0.27	0.36	0.33	0.4	0.29
Hayes-roth	-0.03	-0.13	0.25	0.24	0.16	0.34	0.15	**0.35**	0.25	0.15	0.34	0.05	0.34	0.02
Heart	0.15	0.21	-0.02	0.45	**0.59**	0.18	0.15	0.11	0.04	0.15	0.11	0.18	0.21	0.25
Hepatitis	-0.25	-0.25	-0.04	0.38	**0.58**	-0.04	-0.25	-0.04	-0.04	0.17	-0.25	-0.25	-0.04	0.21
Housing	0.08	0.21	0.08	0.39	**0.45**	0.08	0.21	0.21	0.08	0.02	0.15	0.21	0.08	0.21
Imports	0.22	0.09	0.09	0.22	-0.04	0.09	**0.48**	0.22	0.35	0.09	0.35	0.22	**0.48**	0.35
Ionosphere	0.69	0.81	0.81	**0.88**	0.69	0.81	0.81	0.56	0.5	0.81	0.81	0.81	0.81	0.78
Iris	0.95	0.95	0.95	0.89	0.89	0.89	**1**	0.89	0.84	0.89	**1**	0.95	0.84	0.78
Led	0.58	0.42	0.06	0.48	0.48	0.17	0.65	0.52	-0.05	0.06	**0.69**	0.58	0.12	-0.04
Liver	0.2	0.14	0.14	0.02	0.2	0.08	0.14	-0.1	**0.33**	0.26	0.02	0.2	0.14	0.19
Lung-cancer	**0.47**	**0.47**	0.22	**0.47**	0.06	**0.47**	**0.47**	0.06	**0.47**	**0.47**	**0.47**	**0.47**	0.06	0.06
Multiple-features	0.79	0.72	0.77	**0.94**	0.86	0.88	0.83	0.59	0.82	0.76	0.12	0.02	0.79	0.57
Optdigits	0.85	0.91	0.94	0.91	0.84	0.94	**0.95**	0.79	0.86	0.92	**0.95**	0.92	0.8	0.69
Pageblocks	0.03	0.55	0.24	0.56	0.56	0.18	0.1	0.51	0.02	**0.61**	0.25	0.13	0.28	0.5
Sat	0.74	0.48	0.71	0.45	0.74	0.72	0.7	0.53	0.69	0.6	**0.75**	**0.75**	0.69	0.54
Seeds	0.86	**0.96**	0.68	0.89	0.93	0.79	0.82	0.75	0.79	0.75	0.86	0.86	0.89	0.74
Semeion	0.71	0.57	0.77	0.8	0.47	0.8	0.78	0.58	0.76	0.69	**0.81**	0.73	0.79	0.09
Sonar	0.12	0.22	0.22	0.31	0.22	0.22	0.26	0.12	0.12	0.12	0.22	0.22	**0.36**	0.2
Soybean-small	**1**	0.84	**1**	**1**	**1**	**1**	**1**	0.68	**1**	**1**	**1**	**1**	0.68	0.46
Spectf	0.43	0.08	0.43	0.31	0.2	0.31	0.43	0.31	**0.54**	0.2	0.08	0.43	0.2	0.31
Survival	0.09	0	0.13	0.18	0.23	0.13	0.32	0.32	0.04	0.23	**0.36**	0.23	0.18	-0.03
Systhetic-control	0.64	0.59	0.91	0.59	0.67	0.9	0.8	0.91	0.8	**0.95**	0.03	0.76	0.89	0.45
Texture	0.83	**0.95**	0.91	**0.95**	0.8	0.92	0.93	0.86	0.87	0.93	0.9	0.87	0.91	0.77
User-knowledge	0.52	0.57	0.47	0.62	0.52	0.48	**0.63**	0.39	0.56	0.39	0.55	0.57	**0.63**	0.54
Vehicle	0.28	0.49	0.21	**0.65**	0.41	0.43	0.39	0.32	0.42	0.3	0.43	0.34	0.48	0.4
Vertebra-column	0.56	0.5	0.57	0.58	0.5	0.57	0.57	0.44	0.51	0.48	**0.6**	0.56	0.45	0.32
Vowels	0.83	0.78	0.77	0.94	0.55	0.92	**0.96**	0.72	0.86	0.76	0.92	0.89	0.86	0.7
Waveform	0.59	0.5	0.52	0.5	**0.6**	0.52	0.54	0.43	0.57	0.48	0.55	**0.6**	0.06	0.24
Wine	0.47	0.67	0.33	**0.79**	0.63	0.38	0.31	0.38	0.43	0.42	0.38	0.47	0.42	0.49
Yeastgalactose	0.88	0.86	0.88	0.92	0.79	0.88	**0.97**	0.83	0.88	0.88	**0.97**	**0.97**	0.86	0.62
Zoo	0.82	**1**	**1**	**1**	**1**	**1**	**1**	0.56	0.82	0.74	**1**	**1**	0.66	0.41
	0.46	0.47	0.46	**0.57**	0.47	0.47	0.52	0.42	0.47	0.47	0.49	0.47	0.45	0.38

Table 10: Detailed results of the methods with the parameters selected using Uniform Objects over all datasets of the Type I experiments (single source-class inliers) with respect to AdjustedPrec@n. We visualize the distribution of these values in Figure 10(b).

AdjustedPrec@n	ABOD	Auto Enc.	GLOSH	GMM	iForest	KNN G.	SVDD	KNN L.	LOCI	LOF	LP	Parzen	SOD	DSVDD
Abalone	0.31	0.2	0.09	0.21	**0.33**	0.17	0.29	0.1	0.31	0.12	0.14	0.31	0.22	0.19
Arrhythmia	0.31	0.26	0.31	**0.46**	0.31	0.41	0.36	0.12	0.36	0.26	-0.03	-0.03	0.31	0.2
Artificial-characters	0.31	0.34	0.3	**0.39**	0.22	0.31	0.15	0.32	0.37	0.31	0.33	0.27	0.2	0.33
Balancescale	0.35	0.49	0.43	**0.8**	0.45	0.49	0.4	0.5	0.46	0.49	0.42	0.42	0.32	0.44
Ball-bearing	0.75	0.81	0.87	**0.97**	0.95	0.86	0.26	0.78	0.76	0.89	0.87	0.68	0.94	0.86
Biomed	0.24	0.42	0.12	0.36	0.36	0.18	0.3	**0.53**	0.3	0.24	0.36	0.36	0.18	0.37
Breast	0.83	0.57	0.78	0.48	**0.93**	0.76	0.8	0.67	0.89	0.54	0.78	0.89	0.76	0.47
Cancer	0.13	0.21	**0.28**	0.13	0.06	**0.28**	0.13	0.21	**0.28**	0.13	**0.28**	**0.28**	0.13	0.02
Car-evaluation	0.58	0.58	0.67	**0.87**	0.58	0.76	0.59	0.64	0.82	0.64	0.56	0.62	0.37	0.57
Cardiotocography	0.13	0.74	0.12	**0.91**	0.61	0.08	-0.02	0.11	0.12	0.14	0.24	0.01	0.24	0.52
Cellcycle	0.45	0.29	0.39	0.43	0.38	0.43	0.31	0.27	0.43	**0.54**	0.36	0.36	0.25	0.24
Cnae	0.33	0.28	0.38	**0.6**	-0.03	0.39	0.44	0.3	0.29	0.34	0.36	-0.04	0.59	0.06
Colon	-0.12	**0.25**	-0.12	0.06	**0.25**	-0.12	-0.12	0.06	0.06	0.06	**0.25**	**0.25**	-0.12	0.14
Delft	0.24	0.33	0.51	**0.74**	0.12	0.52	0.33	0.55	0.58	0.61	0.59	0.16	0.6	0.05
Dermatology	0.7	0.67	0.72	**0.91**	0.75	0.72	0.75	0.48	0.73	0.58	0.82	0.56	0.73	0.33
Diabetes	0.22	0.05	0.21	0.09	0.19	0.23	0.02	0.08	0.18	0.18	**0.25**	0.21	0.12	0.06
Ecoli	**0.76**	0.17	**0.76**	0.17	0.53	0.65	0.53	0.65	**0.76**	**0.76**	**0.76**	**0.76**	0.17	0.65
Flare	0.37	0.41	0.34	**0.45**	0.13	0.36	0.29	0.39	0.38	0.42	0.28	0.43	0.42	0.26
Glass	0.31	0.31	0.26	**0.42**	0.26	0.22	0.19	0.16	0.33	0.33	0.32	0.29	0.36	0.13
Hayes-roth	-0.03	0.17	0.26	-0.03	-0.03	0.06	-0.12	0.26	0.25	**0.43**	0.06	0.15	0.34	0.18
Heart	0.15	0.11	0.11	0.28	**0.59**	0.18	0.15	0.08	0.08	-0.06	0.04	0.15	0.32	0.25
Hepatitis	-0.25	0.38	-0.25	0.38	**0.58**	-0.04	-0.04	-0.04	-0.04	-0.25	-0.25	-0.25	-0.04	0.17
Housing	0.08	0.21	0.15	**0.33**	**0.33**	0.15	0.15	0.21	0.08	-0.04	0.15	0.02	0.02	0.16
Imports	0.22	0.09	0.09	0.35	-0.04	0.35	0.09	0.22	0.35	0.09	0.22	0.09	**0.61**	0.4
Ionosphere	0.69	0.75	0.81	**0.88**	0.69	0.69	0.69	0.63	0.44	0.63	0.75	0.75	**0.88**	0.8
Iris	**0.95**	**0.95**	**0.95**	**0.95**	0.84	**0.95**	**0.95**	0.84	0.89	0.89	**0.95**	0.84	0.84	0.73
Led	0.58	0.1	0.06	0.06	0.29	0.44	**0.69**	0.37	-0.05	0.26	**0.69**	0.58	0.01	0.11
Liver	**0.2**	0.02	0.14	0.14	0.14	0.08	-0.1	0.14	**0.2**	**0.2**	0.02	**0.2**	**0.2**	0.07
Lung-cancer	**0.47**	**0.47**	0.22	**0.47**	0.06	**0.47**	**0.47**	0.19	**0.47**	**0.47**	**0.47**	**0.47**	0.06	0.1
Multiple-features	0.79	0.76	0.66	**0.91**	0.84	0.84	0.35	0.62	0.78	0.66	0.02	0.02	0.76	0.23
Optdigits	0.85	0.91	0.93	0.91	0.79	**0.94**	0.71	0.79	0.87	0.93	0.83	0.88	0.81	0.65
Pageblocks	0.03	0.2	0.32	**0.65**	0.45	0.18	0.34	0.49	0.52	0.63	0.25	0.02	0.3	0.37
Sat	0.74	0.47	0.67	0.46	0.74	0.71	0.49	0.44	0.7	0.56	**0.75**	0.73	0.69	0.52
Seeds	0.86	0.89	0.68	**0.96**	0.89	0.75	0.86	0.61	0.79	0.71	0.89	0.82	0.89	0.54
Semeion	0.71	0.57	0.79	0.79	0.68	0.78	0.68	0.58	0.73	0.74	**0.8**	0.72	**0.8**	0.12
Sonar	0.12	0.22	0.02	0.12	0.12	0.17	0.02	0.22	0.07	-0.03	-0.08	0.02	**0.51**	-0.02
Soybean-small	**1**	0.84	**1**	**1**	0.84	**1**	**1**	0.84	**1**	**1**	**1**	**1**	**1**	0.54
Spectf	0.43	0.43	0.31	0.31	0.2	0.43	**0.54**	0.43	**0.54**	0.43	0.08	**0.54**	0.08	0.24
Survival	0.09	0	0.13	0.09	0.18	0.13	0.18	0.18	0.09	**0.32**	0.27	0.23	0.13	0.02
Systhetic-control	0.64	0.59	0.9	0.87	0.65	0.85	0.54	0.88	0.82	**0.93**	0.03	0.73	0.91	0.3
Texture	0.83	0.9	0.92	**0.93**	0.77	0.89	0.7	0.84	0.86	0.92	0.67	0.77	0.91	0.73
User-knowledge	0.52	**0.7**	0.45	0.53	0.52	0.51	0.35	0.37	0.51	0.46	0.37	0.52	0.69	0.59
Vehicle	0.28	0.37	0.37	**0.65**	0.44	0.38	0.05	0.25	0.4	0.23	0.43	0.2	0.52	0.23
Vertebra-column	0.56	0.42	0.48	0.46	0.43	0.56	0.58	0.38	0.56	0.52	**0.6**	0.56	0.41	0.3
Vowels	0.83	0.62	0.86	0.85	0.51	**0.88**	0.23	0.77	0.87	0.76	0.48	0.68	0.77	0.64
Waveform	0.59	0.5	0.54	0.53	**0.61**	0.54	0.59	0.47	0.59	0.5	0.49	0.6	0.31	0.25
Wine	0.47	0.59	0.52	**0.71**	0.67	0.38	0.47	0.38	0.43	0.47	0.38	0.47	0.39	0.37
Yeastgalactose	0.88	0.86	0.71	0.81	0.88	0.88	**0.97**	0.57	0.88	0.88	**0.97**	**0.97**	0.88	0.55
Zoo	0.82	**1**	**1**	**1**	**1**	**1**	**1**	0.66	0.82	0.74	**1**	0.82	0.82	0.29
	0.46	0.46	0.45	**0.55**	0.47	0.49	0.4	0.42	0.49	0.46	0.43	0.43	0.46	0.33

Table 11: Detailed results of the methods with the parameters selected using Perturbation over all datasets of the Type I experiments (single source-class inliers) with respect to AdjustedPrec@n. We visualize the distribution of these values in Figure 10(c).

AdjustedPrec@n	ABOD	Auto Enc.	GLOSH	GMM	iForest	KNN G.	SVDD	KNN L.	LOCI	LOF	LP	Parzen	SOD	DSVDD
Abalone	0.31	0.16	0.18	0.22	**0.33**	0.19	0.15	0.1	0.31	0.12	0.17	0.31	0.19	0.23
Arrhythmia	0.31	0.26	0.36	**0.46**	0.31	0.41	0.36	0.26	0.36	0.26	-0.03	-0.03	0.36	0.2
Artificial-characters	0.31	0.37	0.32	**0.39**	0.23	0.32	0.2	0.32	0.37	0.34	0.33	0.3	0.18	0.35
Balancescale	0.35	0.42	0.42	**0.78**	0.42	0.43	0.39	0.46	0.44	0.45	0.36	0.42	0.32	0.39
Ball-bearing	0.75	0.96	0.86	**0.97**	0.96	0.86	0.72	0.82	0.75	0.89	0.87	0.75	0.94	0.89
Biomed	0.24	0.42	0.12	0.3	0.36	0.18	**0.47**	0.24	0.24	0.42	0.36	0.3	0.12	0.28
Breast	0.83	0.54	0.39	0.43	**0.93**	0.52	0.8	0.39	0.8	0.33	0.76	0.63	0.74	0.54
Cancer	0.13	0.21	**0.28**	0.13	0.06	0.21	**0.28**	0.21	0.21	0.21	**0.28**	0.21	0.21	0.05
Car-evaluation	0.58	0.6	0.7	**0.86**	0.59	0.68	0.64	0.7	0.82	0.64	0.48	0.62	0.37	0.6
Cardiotocography	0.13	0.74	0.17	**0.91**	0.61	0.16	0.15	0.17	0.15	0.12	0.24	0.07	0.31	0.81
Cellcycle	**0.45**	0.29	0.36	0.43	0.22	0.13	0.28	0.25	0.31	0.24	0.36	0.4	0.25	0.31
Cnae	0.33	0.27	0.28	0.6	0.04	0.27	0.43	0.24	0.33	0.27	0.29	-0.04	**0.64**	0.06
Colon	-0.12	**0.44**	**0.44**	0.06	0.25	-0.12	-0.12	0.25	0.06	0.06	0.25	0.25	-0.12	-0.09
Delft	0.24	0.55	0.73	**0.74**	0.13	0.62	0.5	0.65	0.64	0.63	0.59	0.47	0.51	0.15
Dermatology	0.7	0.67	0.74	**0.91**	0.47	0.77	0.73	0.61	0.77	0.71	0.82	0.68	0.88	0.45
Diabetes	0.22	0.13	**0.28**	0.06	0.21	0.23	0.08	0.09	0.21	0.25	0.25	0.21	0.15	0.04
Ecoli	**0.76**	0.29	**0.76**	0.17	**0.76**	0.65	0.41	0.65	0.53	0.06	0.65	**0.76**	0.17	0.46
Flare	0.37	0.42	0.29	**0.47**	0.12	0.33	0.34	0.33	0.38	0.25	0.3	0.41	0.37	0.26
Glass	0.31	0.39	0.21	**0.44**	0.12	0.33	0.18	0.16	0.16	0.09	0.36	0.29	0.36	0.3
Hayes-roth	-0.03	0.17	0.35	**0.42**	-0.14	0.05	-0.12	0.15	0.16	0.25	-0.03	0.06	0.14	0.13
Heart	0.15	0.21	0.01	0.28	**0.59**	0.11	0.21	0.04	0.15	0.15	0.04	0.18	0.35	0.2
Hepatitis	-0.25	-0.25	-0.04	0.38	**0.58**	-0.04	-0.25	-0.04	-0.04	-0.25	-0.25	-0.25	-0.04	0.04
Housing	0.08	0.21	0.08	0.39	**0.51**	0.08	0.08	0.15	0.08	0.02	0.15	0.21	0.02	0.27
Imports	0.22	0.22	**0.48**	0.35	0.09	-0.04	0.09	**0.48**	0.09	0.09	0.22	0.35	0.35	0.45
Ionosphere	0.69	**0.88**	0.81	**0.88**	0.56	0.81	0.69	0.63	0.5	0.75	0.75	0.75	**0.88**	0.78
Iris	**0.95**	**0.95**	**0.95**	**0.95**	0.84	**0.95**	**0.95**	0.84	0.84	0.89	**0.95**	**0.95**	0.84	0.72
Led	0.58	0.06	-0.05	-0.05	0.43	0.33	**0.69**	0.48	-0.05	0.37	**0.69**	0.58	0.12	0.04
Liver	0.2	0.2	0.14	0.08	0.2	0.08	-0.1	0.14	**0.33**	0.26	0.02	0.2	0.14	0.13
Lung-cancer	**0.47**	**0.47**	**0.47**	**0.47**	**0.47**	**0.47**	**0.47**	**0.47**	**0.47**	**0.47**	**0.47**	**0.47**	0.06	0.2
Multiple-features	0.79	0.76	0.73	**0.91**	0.77	0.88	0.61	0.61	0.79	0.8	0.02	0.02	0.77	0.43
Optdigits	0.85	0.91	0.94	0.91	0.81	**0.95**	0.86	0.8	0.88	0.94	0.83	0.92	0.8	0.66
Pageblocks	0.03	0.52	0.17	0.54	0.53	0.15	0.34	0.56	0.52	**0.67**	0.25	0.02	0.31	0.5
Sat	0.74	0.47	0.67	0.46	0.73	0.72	0.5	0.53	0.68	0.56	**0.75**	**0.75**	0.69	0.52
Seeds	0.86	0.89	0.61	**0.96**	0.89	0.79	0.82	0.75	0.79	0.71	0.89	0.86	0.89	0.7
Semeion	0.71	0.56	0.74	0.79	0.51	0.72	0.7	0.56	0.68	0.74	**0.8**	0.54	0.72	0.07
Sonar	0.12	0.12	0.22	0.12	0.22	0.22	0.07	0.12	0.17	0.02	0.07	0.22	**0.26**	0.11
Soybean-small	**1**	**1**	0.68	**1**	0.84	0.84	**1**	0.84	**1**	**1**	**1**	**1**	0.55	0.74
Spectf	0.43	0.43	0.31	0.31	0.2	0.43	0.43	0.31	0.43	0.31	0.08	**0.54**	0.2	0.11
Survival	0.09	0	0.13	0.23	0.23	0.18	0.23	0.18	0.04	**0.32**	0.27	0.23	0.04	0.02
Systhetic-control	0.64	0.53	0.86	0.87	0.63	0.85	0.57	0.88	0.83	**0.92**	0.03	0.76	0.81	0.31
Texture	0.83	0.9	0.92	**0.93**	0.79	0.91	0.7	0.85	0.86	0.92	0.71	0.8	0.91	0.76
User-knowledge	0.52	0.68	0.38	0.56	0.47	0.47	0.41	0.34	0.57	0.34	0.35	0.52	**0.69**	0.56
Vehicle	0.28	0.37	0.31	**0.65**	0.42	0.35	0.08	0.28	0.42	0.27	0.43	0.28	0.52	0.4
Vertebra-column	0.56	0.42	0.48	0.46	0.52	0.59	**0.61**	0.47	0.56	0.5	0.6	0.56	0.56	0.3
Vowels	0.83	0.78	**0.91**	0.89	0.51	0.88	0.41	0.8	0.82	0.83	0.66	0.85	0.72	0.72
Waveform	0.59	0.5	0.54	0.52	**0.6**	0.54	0.56	0.47	0.57	0.51	0.49	**0.6**	0.31	0.25
Wine	0.47	0.59	0.52	**0.71**	0.67	0.42	0.36	0.34	0.43	0.51	0.38	0.47	0.62	0.57
Yeastgalactose	0.88	0.77	**0.97**	0.81	0.57	**0.97**	**0.97**	0.6	0.88	0.88	**0.97**	**0.97**	0.75	0.58
Zoo	0.82	0.82	0.74	**1**	**1**	**1**	**1**	0.74	0.74	0.74	**1**	0.82	0.61	0.4
	0.46	0.47	0.47	**0.55**	0.47	0.47	0.43	0.43	0.47	0.45	0.43	0.45	0.44	0.37

Table 12: Detailed results of the methods with the parameters selected using Cross-validation over all datasets of the Type I experiments (single source-class inliers) with respect to MCC. We visualize the distribution of these values in Figure 4(c).

MCC	ABOD	Auto Enc.	GLOSH	GMM	iForest	KNN G.	SVDD	KNN L.	LOCI	LOF	LP	Parzen	SOD	DSVDD
Abalone	0.25	**0.37**	0.25	0.31	0.35	0.32	0.32	0.28	**0.37**	0.28	0.29	**0.37**	0.3	0.24
Arrhythmia	0.27	0.37	0.33	0	**0.4**	0.33	0	0.3	0.33	0.3	0	0	0.22	0.27
Artificial-characters	0.35	0.52	0.43	**0.55**	0.26	0.43	0.41	0.38	0.4	0.44	0.4	0.26	0.38	0.49
Balancescale	0.46	0.61	0.54	**0.64**	0.44	0.52	0.34	0.54	0.42	0.51	0.39	0.45	0.36	0.54
Ball-bearing	0.63	**0.96**	0.87	**0.96**	**0.96**	0.84	0.62	0.81	0.73	0.87	0.07	0.5	0.95	0.88
Biomed	0.32	0.15	0.35	0.32	0.32	0.35	0.1	0.45	0.34	0.37	0.11	0.08	0.21	**0.54**
Breast	0.78	0.55	**0.92**	0.39	0.86	**0.92**	0.79	0.77	**0.92**	0.91	0.8	0.9	0.78	0.71
Cancer	0.24	0.03	0.23	0	0.04	0.21	0.27	0.23	0.27	0.31	0	0.19	**0.36**	0.12
Car-evaluation	0.59	0.74	0.82	**0.91**	0.51	0.69	0.26	0.66	0.76	0.74	0.63	0.66	0.6	0.61
Cardiotocography	0.08	0.79	0.15	**0.91**	0.56	0.11	0.19	0.17	0.11	0.19	0.15	0	0.32	0.84
Cellcycle	0.29	0.18	0.4	0.31	0.23	0.47	0.22	0.28	0.26	0.37	0.47	**0.52**	0.24	0.26
Cnae	0.13	0.4	0.18	**0.58**	-0.06	0.17	0.23	0.23	0.17	0.26	0.24	0	0.44	0.17
Colon	0.1	0	-0.04	0	0.24	-0.17	0	-0.17	0.14	0.29	0	0	**0.32**	0.04
Delft	0.17	0.34	**0.75**	0.3	0.08	0.57	0.12	0.71	0.58	0.72	0.18	0.32	0.69	0.19
Dermatology	0.6	0.31	0.71	0.35	0.61	0.77	0.51	0.66	0.67	0.68	0.62	0.58	**0.87**	0.37
Diabetes	0.14	0.14	0.14	0.04	0.16	0.16	0.09	0.15	0.08	0.16	**0.24**	0.16	0.03	0.19
Ecoli	0.48	0	0.76	0.68	0.72	0.64	0.36	0.72	**0.79**	0.68	0.55	0.76	0.26	0.41
Flare	0.29	**0.43**	0.31	0.4	0.18	0.36	0.38	0.29	0.34	0.33	0.39	0.37	**0.43**	0.38
Glass	0.32	0.04	0.26	0.27	0.22	0.26	0.09	0.29	0.15	0.24	0.22	0.27	**0.34**	0.23
Hayes-roth	0.3	0.34	**0.4**	0.1	0.19	0.35	0.14	0.34	0.34	0.28	0.05	0.27	0.34	0.17
Heart	0.11	0.18	0.09	0.09	**0.64**	0.1	0.08	0.13	0.11	0.03	0.1	0.08	0.15	0.33
Hepatitis	-0.09	0.13	-0.13	0.17	0.33	-0.13	0	-0.2	-0.13	-0.13	0	-0.22	-0.04	**0.34**
Housing	0.07	0.28	0.01	0.32	**0.47**	0.07	0.15	0.12	0.18	0.08	0.05	0.09	0.15	0.31
Imports	0.24	0	0.48	0	**0.54**	0.18	0	0.27	0.34	0.34	0	0	0.32	0.48
Ionosphere	0.61	0.79	**0.85**	0.64	0.49	0.82	0.57	0.63	0.45	0.81	0.69	0.68	0.78	0.77
Iris	**0.92**	0.61	0.79	0.87	0.79	0.78	**0.92**	0.87	0.83	0.89	0.87	0.52	0.9	0.73
Led	0.3	0.1	0.28	0	0.32	0.21	0.32	0.23	0.19	0.3	0.52	**0.53**	0.28	0
Liver	-0.21	0.11	-0.21	**0.15**	-0.16	-0.15	0	-0.21	-0.21	-0.21	0	-0.02	-0.01	0.1
Lung-cancer	0.08	0	**0.5**	0	0.05	0.2	0.21	0.37	0.24	0.29	0	0.39	0.22	-0.07
Multiple-features	0.75	0.77	0.85	0	**0.86**	**0.86**	0.51	0.68	0.77	0.83	0	0	0.73	0.59
Optdigits	0.83	0.92	**0.94**	0.67	0.83	**0.94**	0.26	0.78	0.86	0.93	0.06	0.74	0.82	0.73
Pageblocks	0.17	0.62	0.32	**0.66**	0.59	0.18	0.2	0.59	0.49	0.62	0.19	0.17	0.25	0.48
Sat	0.68	0.65	0.72	0.41	0.69	0.7	0.49	0.59	0.66	0.64	0.42	**0.75**	0.69	0.59
Seeds	0.84	0.6	0.82	0.84	**0.85**	0.71	0.78	0.79	0.82	0.77	0.69	0.72	0.8	0.77
Semeion	0.61	0.09	0.75	0	0.7	0.76	0.16	0.5	0.61	0.74	0	0.67	**0.79**	0.16
Sonar	-0.04	0.15	0.24	0.25	0.05	0.2	0	0.12	0.13	0.2	0.24	0.12	**0.38**	0.18
Soybean-small	0.92	0.12	0.94	0	0.92	0.79	0.38	0.9	0.94	0.92	0.12	0.77	**1**	0.07
Spectf	0.42	0	0.4	0	**0.49**	0.47	0.21	0.47	**0.49**	0.45	0	0.47	0.38	0.07
Survival	0.09	0.01	0	**0.25**	0.15	0.16	0.11	0.07	0.09	0.11	0.06	0.14	0.21	-0.06
Systhetic-control	0.74	0	0.88	0	0.68	0.88	0.44	0.91	0.85	**0.95**	0	0.65	0.91	0.35
Texture	0.76	**0.95**	0.93	0.94	0.75	0.91	0.87	0.86	0.84	0.94	0.89	0.46	0.91	0.8
User-knowledge	0.49	**0.69**	0.51	**0.69**	0.49	0.57	0.3	0.48	0.5	0.51	0.32	0.2	0.66	0.55
Vehicle	0.31	0.51	0.31	**0.66**	0.38	0.31	0.39	0.28	0.25	0.31	0.31	0.15	0.55	0.5
Vertebra-column	0.5	0.34	0.56	0.24	0.53	0.55	0.53	0.62	**0.65**	0.64	0.22	0.53	0.63	0.48
Vowels	0.85	0.23	0.85	0.62	0.45	**0.91**	0.47	0.74	0.79	0.82	0.54	0.41	0.83	0.66
Waveform	0.56	0.56	0.59	0.57	**0.6**	0.58	0.59	0.52	**0.6**	0.58	0.48	0.59	0.46	0.38
Wine	0.5	0.58	0.54	0.42	**0.73**	0.51	0.51	0.53	0.53	0.54	0.35	0.54	0.61	0.36
Yeastgalactose	0.87	0.32	0.91	0.22	0.91	0.91	0.58	0.79	0.9	0.84	0.62	**0.94**	0.89	0.34
Zoo	0.5	0.2	0.67	0.25	**0.72**	0.6	0.11	0.5	0.5	0.58	0.36	0.59	0.53	0.16
	0.41	0.36	**0.49**	0.37	0.47	0.47	0.32	0.45	0.46	**0.49**	0.28	0.37	**0.49**	0.38

Table 13: Detailed results of the methods with the parameters selected using Self-Adaptive Data Shifting (SDS) over all datasets of the Type I experiments (single source-class inliers) with respect to MCC. We visualize the distribution of these values in Figure 11(a).

MCC	ABOD	Auto Enc.	GLOSH	GMM	iForest	KNN G.	SVDD	KNN L.	LOCI	LOF	LP	Parzen	SOD	DSVDD
Abalone	0.25	0.23	0.18	0.22	**0.36**	0.23	0.29	0.17	0.35	0.19	0.31	0.17	0.24	0.22
Arrhythmia	0.33	0.24	0.37	0	0.31	0.37	0.31	0.28	0.35	0.35	0	0	**0.38**	0.29
Artificial-characters	0.36	0.5	0.42	**0.55**	0.26	0.43	0.44	0.4	0.4	0.44	0.43	0.41	0.36	0.47
Balancescale	0.4	0.58	0.56	**0.74**	0.4	0.57	0.34	0.52	0.35	0.57	0.38	0.48	0.43	0.46
Ball-bearing	0.63	**0.93**	0.86	0.84	0.87	0.85	0.83	0.83	0.73	0.9	0.09	0.55	0.89	0.79
Biomed	0.11	0.32	0.25	0.22	0.31	0.14	0.33	0.29	0.31	**0.35**	0.13	0.16	0.15	0.3
Breast	0.7	0.45	0.61	0.52	**0.82**	0.41	0.66	0.73	0.81	0.6	0.71	0.56	0.76	0.53
Cancer	0.23	0.02	0.22	0.27	0.19	0.21	0.16	0.2	**0.3**	0.18	0.14	0.19	0.23	0.07
Car-evaluation	0.54	0.69	0.56	**0.88**	0.3	0.78	0.31	0.78	0.58	0.75	0.57	0.63	0.61	0.57
Cardiotocography	0.08	0.78	0.13	**0.83**	0.44	0.13	0.12	0.1	0.1	0.12	0.15	0	0.17	0.76
Cellcycle	0.28	0.22	0.33	**0.37**	0.27	0.34	0.26	0.04	0.12	0.11	0.34	0.26	0.18	0.23
Cnae	0.13	0.12	0.14	**0.51**	-0.09	0.14	0.29	0.14	0.11	0.19	0.32	0	0.24	0
Colon	0.1	-0.11	0.24	0	0.16	0	0	0.2	0.1	**0.4**	0	0	0.12	-0.03
Delft	0.17	0.36	0.56	0.35	0.16	0.54	0.25	0.63	0.49	**0.67**	0.22	0.47	0.55	0.16
Dermatology	0.57	0.62	0.75	0.46	0.4	0.69	0.31	0.6	0.65	0.65	0.43	0.63	**0.85**	0.48
Diabetes	0.16	-0.01	**0.26**	0.07	0.14	0.17	0.23	0.11	0.06	0.17	0.22	0.13	0.1	0.09
Ecoli	0.48	0.29	0.76	0.37	0.75	0.52	0.52	0.72	0.75	**0.79**	0.65	0.52	0.11	0.43
Flare	0.32	0.39	0.25	0.38	0.18	0.26	0.21	0.18	0.27	0.24	0.34	0.28	**0.42**	0.3
Glass	0.28	0.35	0.23	0.25	0.12	0.11	0.16	0.08	0.22	0.11	0.24	0.28	**0.44**	0.33
Hayes-roth	0.23	0.18	0.27	**0.37**	0.07	0.22	0.01	0.27	0.28	0.29	0.16	0.19	0.25	0
Heart	0.11	0.12	0.08	0.28	**0.52**	0.02	0.17	0.11	-0.01	0	0.14	0.16	0.11	0.23
Hepatitis	-0.13	**0.37**	0.08	0.33	0.31	-0.17	-0.25	0.05	0.05	0.17	-0.1	-0.22	-0.17	0.22
Housing	0.19	0.23	0.07	**0.36**	0.31	0.09	0.17	0.06	0.23	0.07	0.11	0.12	0.18	0.26
Imports	0.18	-0.06	0.08	**0.44**	0.04	0.18	0.13	0.25	0.34	0.13	0.13	0.35	0.43	0.36
Ionosphere	0.61	0.53	**0.73**	0.42	0.64	0.71	0.5	0.69	0.56	0.71	0.5	0.61	0.67	0.68
Iris	0.86	0.79	0.75	0.71	0.84	0.81	0.63	0.79	0.73	**0.87**	0.82	0.76	0.76	0.6
Led	0.35	0.35	0.26	0.05	0.25	0.16	0.3	0.1	0.11	0.24	0.47	**0.58**	0.13	0.09
Liver	-0.16	0.04	0.06	**0.18**	-0.01	-0.01	0.05	-0.11	0.01	0.07	0.14	0.09	0.16	0.09
Lung-cancer	0.08	0	0.33	0	-0.14	0.2	0	0.37	0.17	0.13	0	**0.6**	0.53	0
Multiple-features	0.74	0.65	0.79	0	**0.87**	0.85	0.75	0.63	0.72	0.71	0	0	0.76	0.53
Optdigits	0.8	0.88	0.91	0.87	0.82	**0.93**	0.63	0.78	0.75	0.9	0.11	0.9	0.8	0.64
Pageblocks	0.01	0.45	0.2	**0.51**	0.48	0.14	0.22	0.39	0.17	0.45	0.18	0.03	0.23	0.4
Sat	0.67	0.46	0.66	0.46	**0.7**	0.66	0.69	0.53	0.64	0.57	0.49	0.69	0.67	0.54
Seeds	0.81	0.82	0.75	0.84	**0.9**	0.81	0.72	0.79	0.79	0.75	0.74	0.75	0.77	0.71
Semeion	0.32	0.5	0.52	0	0.4	0.47	0.07	0.33	0.12	0.5	0	0.42	**0.76**	0.08
Sonar	-0.08	0.07	0.05	**0.31**	0.28	0.12	0.25	0.19	0.18	0.08	0.23	0.12	0.29	0.21
Soybean-small	**1**	0.12	**1**	0.12	0.85	0.81	0	0.81	0.85	0.92	0	0.71	0.41	0.3
Spectf	0.41	0.26	0.43	0	0.49	0.34	-0.07	0.35	0.34	0.44	0	0.43	**0.51**	0.3
Survival	0.16	0.19	0.16	0.11	0.22	0.16	0.27	0.18	0.09	**0.33**	0.22	0.19	0.03	-0.02
Systhetic-control	0.73	0.57	**0.91**	0	0.7	0.86	0.29	0.88	0.84	0.89	0	0.5	0.84	0.35
Texture	0.74	**0.92**	0.88	**0.92**	0.74	0.87	0.82	0.84	0.84	0.91	0.87	0.76	0.87	0.73
User-knowledge	0.49	0.62	0.54	0.37	0.5	0.56	0.29	0.35	0.49	0.54	0.3	0.45	**0.66**	0.53
Vehicle	0.28	0.5	0.27	**0.65**	0.39	0.3	0.4	0.3	0.28	0.27	0.34	0.2	0.5	0.42
Vertebra-column	0.61	0.53	0.55	**0.64**	0.5	0.57	0.58	0.53	0.57	0.55	0.39	0.57	0.53	0.28
Vowels	0.78	0.79	0.72	0.77	0.49	**0.87**	0.51	0.73	0.73	0.78	0.66	0.85	0.86	0.66
Waveform	0.54	0.58	0.59	0.54	**0.6**	0.58	0.44	0.5	0.56	0.54	0.33	0.58	0.25	0.29
Wine	0.49	0.58	0.37	**0.73**	0.61	0.47	0.36	0.46	0.43	0.44	0.41	0.51	0.28	0.43
Yeastgalactose	0.89	0.75	**0.91**	0.19	0.76	**0.91**	0.21	0.76	0.8	0.78	0.28	0.77	0.89	0.36
Zoo	0.67	0.36	**0.78**	0.42	0.65	0.72	0	0.48	0.65	0.43	0.28	0.46	0.51	0.23
	0.4	0.41	**0.46**	0.4	0.43	0.43	0.31	0.42	0.42	0.45	0.28	0.38	0.44	0.35

Table 14: Detailed results of the methods with the parameters selected using Uniform Objects over all datasets of the Type I experiments (single source-class inliers) with respect to MCC. We visualize the distribution of these values in Figure 11(b).

MCC	ABOD	Auto Enc.	GLOSH	GMM	iForest	KNN G.	SVDD	KNN L.	LOCI	LOF	LP	Parzen	SOD	DSVDD
Abalone	0.17	0.03	0.01	0.03	**0.3**	0.04	0.18	0.13	0.29	0.08	0.18	0.1	0.1	0.11
Arrhythmia	0.31	0.25	0.22	0	0.18	0.22	**0.33**	0.22	0.26	0.19	0	0	0.22	0.22
Artificial-characters	0.26	0.36	0.26	**0.46**	0.19	0.25	0.27	0.33	0.29	0.33	0.45	0.22	0.27	0.35
Balancescale	0.46	0.53	0.55	**0.77**	0.46	0.61	0.53	0.58	0.46	0.51	0.61	0.45	0.36	0.48
Ball-bearing	0.39	0.34	0.26	**0.96**	0.95	0.24	0.27	0.39	0.35	0.33	0.09	0.25	0.65	0.69
Biomed	0.27	0.19	0.27	0.3	0.35	0.39	0.17	0.22	**0.44**	0.22	0.2	0.39	0.3	0.29
Breast	0.69	0.5	0.72	0.3	0.86	0.68	0.8	0.53	0.87	0.5	0.75	**0.88**	0.77	0.41
Cancer	0.21	0.17	0.21	0.32	0.06	0.15	**0.37**	0.21	0.21	0.04	0.14	0.26	0	0.03
Car-evaluation	0.57	0.64	0.81	**0.9**	0.51	0.85	0.67	0.76	0.81	0.74	0.63	0.7	0.54	0.63
Cardiotocography	0.01	**0.64**	0.01	0.57	0.11	0.01	0.05	0.02	0.04	0.05	0.14	0.04	0.04	0.27
Cellcycle	0.29	0.15	**0.4**	0.38	0.11	0.33	0.35	0.31	0.34	0.26	0.35	0.39	0.19	0.21
Cnae	0.07	0.05	0.05	**0.57**	-0.02	0.05	0.2	0.09	0.09	0.08	0.13	0	0.13	-0.03
Colon	0.1	-0.11	0.2	0	**0.35**	0.2	0.18	0.1	-0.04	0.16	0	0	0.21	0.1
Delft	0.2	0.32	0.32	0.45	0.04	0.36	0.38	0.48	0.45	0.48	**0.55**	0.16	0.32	0.1
Dermatology	0.22	0.7	0.2	0.69	0.4	0.33	0.36	0.25	0.31	0.44	**0.76**	0.25	0.66	0.4
Diabetes	0.02	0.06	-0.04	0	0.18	0.01	-0.03	0.01	0.07	0.01	**0.19**	0.06	0	0.02
Ecoli	0.55	0.25	**0.76**	0.34	0.58	0.52	0.61	0.72	0.5	**0.76**	0.55	0.72	-0.01	0.46
Flare	0.31	0.3	0.24	0.41	0.11	0.25	0.38	0.29	0.26	0.31	0.36	0.28	**0.44**	0.27
Glass	0.24	0.4	0.25	0.24	0.25	0.16	0.18	0.17	0.16	0.19	0.39	**0.42**	0.27	0.08
Hayes-roth	0.3	0.4	0.37	-0.05	0.28	0.37	0.09	0.26	0.39	**0.42**	0.19	0.34	0.31	0.2
Heart	0.2	0	0.06	0.27	**0.37**	0.05	0.03	0.13	-0.08	0.06	0.15	0.09	-0.09	0.15
Hepatitis	-0.09	0.29	-0.09	**0.38**	0	-0.09	0	-0.13	-0.09	-0.09	-0.17	-0.09	-0.09	0.2
Housing	0.06	0.09	0.05	0.19	**0.22**	0.05	0.05	0.12	0.18	0.07	0.12	0.06	0.09	0.12
Imports	-0.04	-0.04	-0.04	**0.41**	0.22	0.08	0.13	0.05	0.34	0.16	0.22	0.08	0.28	0.29
Ionosphere	0.55	0.73	**0.81**	0.79	0.69	0.7	0.62	0.63	0.36	0.68	0.72	0.78	**0.81**	0.77
Iris	0.86	**0.95**	0.75	0.92	0.79	0.76	0.84	0.78	0.79	0.85	0.92	0.84	0.78	0.68
Led	0.32	0.05	0.37	0	0.23	0.25	0.45	0.23	0.14	0.2	0.57	**0.61**	0.27	0.13
Liver	-0.16	0.01	-0.21	-0.11	-0.04	-0.11	-0.04	0.08	-0.04	-0.1	0.14	-0.11	**0.27**	0.11
Lung-cancer	0.03	0	**0.47**	0	0.22	0.33	0	0.28	0	0	0	**0.47**	0.26	-0.04
Multiple-features	0.37	**0.73**	0.36	0	0.2	0.41	0.34	0.38	0.47	0.36	0	0	0.33	0.14
Optdigits	**0.67**	0.43	0.55	0.5	0.28	0.52	0.61	0.55	0.62	0.58	0.1	0.55	0.44	0.47
Pageblocks	0.04	0.22	0.19	0.32	0.49	0.19	0.19	0.51	0.17	**0.6**	0.19	0.18	0.13	0.19
Sat	0.53	0.24	0.25	0.2	**0.64**	0.25	0.39	0.26	0.53	0.28	0.49	0.33	0.39	0.33
Seeds	0.78	0.78	0.78	**0.87**	**0.87**	0.72	0.71	0.66	0.78	0.8	0.76	0.8	0.64	0.53
Semeion	0.43	0.5	0.34	0	0.27	0.41	**0.68**	0.39	0.37	0.39	0.04	0.4	0.47	0.17
Sonar	0	0.07	0.09	0.17	0.1	0.09	-0.04	0.2	-0.08	-0.09	-0.13	-0.08	**0.35**	0.05
Soybean-small	**1**	0	0.94	0	0.92	0.75	0.38	0.69	0.9	0.92	0.29	0.79	0.69	0.18
Spectf	0.41	0.21	0.47	0	0.42	0.34	**0.5**	0.38	0.34	0.38	0	0.36	0.32	0.33
Survival	0.09	0.05	0.14	0.04	0.13	0.07	0.13	**0.2**	0.07	0.08	0.11	0.07	0.17	0.05
Systhetic-control	0.72	0.57	0.87	0	0.67	0.87	0.66	0.85	0.83	**0.88**	0	0.75	0.84	0.31
Texture	0.43	0.65	0.58	0.65	0.59	0.54	0.6	0.58	0.65	**0.68**	0.54	0.47	0.54	0.56
User-knowledge	0.49	**0.74**	0.55	0.69	0.47	0.53	0.52	0.42	0.53	0.53	0.45	0.56	0.64	0.61
Vehicle	0.17	0.15	0.21	**0.45**	0.11	0.15	0.23	0.22	0.2	0.23	0.43	0.12	0.27	0.27
Vertebra-column	0.36	0.36	0.45	0.36	0.4	0.49	**0.63**	0.39	0.5	0.44	0.39	0.38	0.35	0.3
Vowels	0.74	0.61	**0.78**	0.77	0.46	0.77	0.4	0.63	0.77	0.72	0.51	0.6	0.72	0.62
Waveform	0.41	0.49	0.49	0.5	**0.57**	0.49	0.46	0.48	0.18	0.49	0.55	0.47	0.13	0.32
Wine	0.46	0.51	0.38	**0.66**	0.53	0.4	0.49	0.3	0.49	0.35	0.44	0.5	-0.05	0.25
Yeastgalactose	0.91	0.79	0.83	0.36	0.84	0.93	0.59	0.63	0.87	0.71	0.71	0.91	**0.94**	0.41
Zoo	0.67	0.36	**0.8**	0.42	0.61	0.72	0.2	0.6	0.53	0.5	0.36	0.55	0.63	0.09
	0.35	0.34	0.37	0.36	**0.38**	0.36	0.35	0.36	0.36	0.36	0.32	0.35	0.35	0.28

Table 15: Detailed results of the methods with the parameters selected using Perturbation over all datasets of the Type I experiments (single source-class inliers) with respect to MCC. We visualize the distribution of these values in Figure 11(c).

MCC	ABOD	Auto Enc.	GLOSH	GMM	iForest	KNN G.	SVDD	KNN L.	LOCI	LOF	LP	Parzen	SOD	DSVDD
Abalone	0.21	0.11	0.16	0.1	**0.3**	0.11	0.18	0.13	0.29	0.14	0.18	0.12	0.1	0.12
Arrhythmia	0.31	0.25	0.37	0	0.32	0.34	0.3	0.22	0.26	0.32	0	0	**0.42**	0.26
Artificial-characters	0.27	0.44	0.33	**0.48**	0.22	0.38	0.33	0.34	0.33	0.34	0.45	0.36	0.33	0.42
Balancescale	0.44	0.53	0.55	**0.77**	0.52	0.59	0.52	0.55	0.45	0.51	0.52	0.47	0.42	0.43
Ball-bearing	0.39	0.89	0.71	**0.96**	**0.96**	0.72	0.59	0.75	0.56	0.87	0.09	0.44	0.94	0.88
Biomed	0.21	0.27	0.31	0.3	0.35	0.38	0.24	0.19	**0.44**	0.3	0.2	0.16	0.32	0.33
Breast	0.69	0.51	0.31	0.29	**0.86**	0.4	0.82	0.08	0.85	0.03	0.73	0.64	0.74	0.45
Cancer	0.24	0.17	0.31	0.32	0.23	0.22	**0.33**	0.15	0.29	0.29	0.14	0.3	0.13	-0.05
Car-evaluation	0.54	0.62	**0.85**	0.82	0.5	0.81	0.65	0.79	0.8	0.74	0.59	0.65	0.55	0.64
Cardiotocography	0.06	0.64	0.07	0.57	0.21	0.09	0.08	0.13	0.06	0.12	0.14	0.01	0.17	**0.7**
Cellcycle	0.09	0.17	0.09	0.32	0.14	0.17	0.31	0.2	0.1	0.12	0.3	**0.36**	0.08	0.17
Cnae	0.13	0.14	0.13	**0.57**	-0.02	0.11	0.25	0.13	0.16	0.18	0.21	0	0.39	-0.02
Colon	0.1	0	0.2	0	0.1	0.1	0	0.52	-0.04	**0.56**	0	0	0	-0.06
Delft	0.2	0.54	**0.67**	0.45	0.18	0.58	0.5	0.61	0.61	**0.67**	0.55	0.51	0.45	0.14
Dermatology	0.5	0.7	0.7	0.69	0.34	0.72	0.69	0.54	0.62	0.62	0.74	0.49	**0.86**	0.5
Diabetes	0.02	0.09	0.14	0.05	0.16	0.11	0.04	0.06	0.05	0.12	**0.19**	0.04	0.16	0.04
Ecoli	0.48	0.29	0.68	0.37	0.68	0.52	0.43	**0.72**	0.46	0.23	0.65	**0.72**	-0.01	0.42
Flare	0.25	0.4	0.31	0.41	0.13	0.3	0.38	0.28	0.31	0.3	0.38	0.35	**0.5**	0.22
Glass	0.26	0.26	0.21	0.29	0.11	0.19	0.18	0.12	0.17	0.23	0.22	**0.37**	**0.37**	0.32
Hayes-roth	0.21	**0.4**	0.23	0.15	0.24	0.23	0.06	0.2	0.3	0.24	0.07	0.26	0.31	0.13
Heart	0.08	0.09	0	0.27	**0.47**	-0.08	0.05	0.03	-0.11	-0.02	0.15	-0.02	0.14	0.15
Hepatitis	-0.09	0.14	-0.09	0.38	**0.54**	-0.09	-0.13	-0.13	-0.09	-0.13	-0.17	-0.09	-0.2	0.13
Housing	0.11	0.25	0.08	0.19	**0.42**	0.16	0.12	0.01	0.2	0.04	0.12	0.08	0.06	0.21
Imports	0.18	0.09	**0.54**	0.41	0.27	0.15	0.17	0.22	0.13	0.09	0.22	0.28	0.43	0.5
Ionosphere	0.66	0.79	**0.81**	0.79	0.54	**0.81**	0.72	0.52	0.52	0.68	0.72	0.78	**0.81**	0.78
Iris	0.86	0.87	0.73	**0.95**	0.78	0.76	0.89	0.77	0.8	0.87	0.92	0.89	0.78	0.65
Led	0.29	0	0.2	0	0.16	0.2	0.42	0.22	0.13	0.19	**0.52**	**0.52**	0.29	0.04
Liver	-0.16	0.08	-0.04	**0.17**	-0.01	-0.11	-0.01	-0.04	-0.11	-0.11	0.14	0.06	0.09	0.08
Lung-cancer	0.08	0	0.33	0	**0.36**	0.33	0	0.05	0.17	0.2	0	**0.36**	0.31	0.01
Multiple-features	0.63	**0.73**	0.46	0	0.5	0.5	0.5	0.46	0.54	0.56	0	0	0.58	0.44
Optdigits	0.82	0.83	**0.92**	0.79	0.7	**0.92**	0.83	0.77	0.84	**0.92**	0.1	0.89	0.76	0.63
Pageblocks	0.04	0.5	0.17	0.54	0.57	0.17	0.17	0.56	0.17	**0.63**	0.19	0.04	0.32	0.42
Sat	0.62	0.25	0.4	0.2	**0.65**	0.39	0.43	0.32	0.53	0.31	0.49	0.48	0.39	0.39
Seeds	0.78	0.71	0.73	**0.87**	**0.87**	0.76	0.77	0.63	0.78	0.76	0.78	0.83	0.73	0.72
Semeion	0.22	0.25	0.59	0	0.25	0.58	**0.68**	0.45	0.46	0.59	0.04	0.5	0.4	0.02
Sonar	0.03	0.22	0.19	0.17	0.3	0.23	0.07	-0.04	0	0.17	-0.01	0.12	**0.35**	0.11
Soybean-small	0.9	0	0.9	0	**0.92**	0.9	0.25	0.74	0.7	0.75	0	**0.92**	0.34	0.03
Spectf	0.47	0.21	**0.5**	0	0.45	0.4	0.43	0.31	0.42	0.47	0	0.47	0.35	0.12
Survival	0.05	0.05	0.07	0.05	0.19	0.12	0.03	**0.23**	0.07	0.08	0.13	0.07	0.07	0.04
Systhetic-control	0.73	0.41	0.87	0	0.58	0.86	0.64	0.88	0.82	**0.9**	0	0.81	0.73	0.29
Texture	0.52	0.65	0.58	0.65	0.62	0.55	0.61	0.6	0.67	**0.68**	0.62	0.56	0.54	0.58
User-knowledge	0.47	**0.71**	0.51	0.66	0.42	0.43	0.54	0.35	0.41	0.5	0.47	0.57	0.61	0.58
Vehicle	0.21	0.15	0.24	**0.45**	0.25	0.22	0.28	0.27	0.24	0.23	0.43	0.19	0.35	0.35
Vertebra-column	0.36	0.36	0.45	0.36	0.5	0.48	**0.65**	0.51	0.49	0.4	0.39	0.4	0.47	0.28
Vowels	0.82	0.79	0.84	0.8	0.43	**0.85**	0.51	0.69	0.8	0.79	0.6	0.76	0.78	0.69
Waveform	0.47	0.53	0.53	0.54	0.55	0.53	0.55	0.5	**0.56**	0.55	0.55	0.53	0.13	0.31
Wine	0.5	0.54	0.45	**0.66**	0.61	0.45	0.38	0.47	0.43	0.46	0.41	0.47	0.51	0.53
Yeastgalactose	0.65	0.4	**0.89**	0.36	0.53	**0.89**	0.42	0.61	0.74	0.72	0.42	**0.89**	0.75	0.36
Zoo	0.67	0.09	0.71	0.17	**0.74**	0.58	0.2	0.64	0.49	0.49	0.36	0.53	0.6	0.16
	0.36	0.37	**0.43**	0.37	0.42	0.41	0.37	0.37	0.39	0.4	0.3	0.39	0.4	0.32

Table 16: Detailed results of the methods with the parameters selected using Cross-validation over all datasets of the Type II experiments (multiple source-class inliers) with respect to ROC AUC. We visualize the distribution of these values in Figure 5(a).

ROC AUC	ABOD	Auto Enc.	GLOSH	GMM	iForest	KNN G.	SVDD	KNN L.	LOCI	LOF	LP	Parzen	SOD	DSVDD
Abalone	0.69	0.68	0.63	0.69	0.66	0.69	0.73	0.62	0.67	0.63	**0.74**	0.72	0.61	0.66
Artificial-characters	0.61	0.69	0.69	0.7	0.63	0.68	**0.72**	0.64	0.66	0.7	0.7	0.68	0.67	0.67
Balancescale	0.83	0.68	0.82	0.76	0.84	0.81	0.79	0.79	0.84	**0.85**	0.83	**0.85**	0.69	0.72
Car-evaluation	0.65	0.81	0.89	0.91	0.9	0.91	0.94	0.88	**0.95**	**0.95**	0.93	0.72	0.8	0.79
Cardiotocography	0.58	0.96	0.64	**0.99**	0.91	0.63	0.69	0.64	0.63	0.65	0.69	0.65	0.71	0.98
Cellcycle	0.78	0.83	**0.84**	0.79	0.75	**0.84**	0.78	0.77	0.73	0.77	0.76	**0.84**	0.75	0.76
Cnae	0.57	0.63	0.71	0.79	0.47	0.69	0.66	0.76	0.67	0.78	0.59	0.5	**0.85**	0.66
Dermatology	0.66	0.88	0.86	**0.9**	0.76	0.81	0.8	0.82	0.78	0.86	0.84	0.8	0.86	**0.9**
Flare	0.67	0.76	0.69	0.6	0.61	0.73	0.75	0.72	0.74	0.72	0.75	0.72	**0.78**	0.66
Glass	0.73	0.77	**0.82**	0.78	0.72	0.78	0.79	0.7	0.75	0.81	**0.82**	0.79	0.79	0.75
Hayes-roth	0.79	0.68	0.81	0.84	0.68	0.84	**0.89**	0.72	0.86	0.79	0.86	0.83	0.72	0.69
Iris	0.96	0.97	0.95	0.97	0.88	0.96	**0.98**	0.92	0.95	**0.98**	0.97	**0.98**	0.93	0.95
Led	**0.72**	0.45	0.53	0.67	0.57	0.59	0.64	0.53	0.7	0.65	0.64	0.66	0.56	0.63
Lung-cancer	0.5	0.31	0.47	0.5	0.44	0.5	0.25	0.62	0.34	**0.69**	0.5	0.5	0.5	0.52
Multiple-features	0.69	0.87	0.92	0.93	0.85	0.91	**0.96**	0.87	0.89	0.94	0.9	0.5	0.9	0.9
Optdigits	0.75	0.91	**0.99**	0.92	0.81	**0.99**	**0.99**	0.93	0.88	0.98	0.94	0.97	0.93	0.87
Sat	0.74	0.77	0.84	0.77	0.76	0.83	**0.86**	0.79	0.77	0.82	0.83	0.84	0.85	0.82
Seeds	0.9	0.92	0.94	**0.96**	0.86	0.94	0.95	0.9	0.92	**0.96**	0.95	0.95	0.93	0.93
Semeion	0.67	0.79	0.89	0.79	0.79	0.89	0.86	0.8	0.8	0.89	**0.91**	0.79	0.87	0.76
Soybean-small	0.81	0.96	0.96	**1**	**1**	0.87	0.92	0.92	0.94	**1**	0.99	0.88	**1**	0.95
Systhetic-control	0.71	0.95	0.97	0.82	0.75	0.97	0.96	0.98	0.92	**0.99**	0.97	0.91	0.89	0.9
Texture	0.78	0.93	0.96	**0.98**	0.76	0.95	0.97	0.95	0.88	**0.98**	0.82	0.87	0.96	0.91
User-knowledge	0.71	0.73	0.77	**0.83**	0.76	0.78	**0.83**	0.75	0.75	0.78	0.8	0.8	0.75	0.69
Vehicle	0.61	0.75	0.69	**0.81**	0.69	0.72	0.72	0.61	0.71	0.66	0.74	0.71	0.76	0.72
Vertebra-column	0.59	0.73	0.67	0.69	0.64	0.58	**0.87**	0.67	0.71	0.72	0.65	0.7	0.7	0.79
Vowels	0.71	0.91	0.93	0.95	0.56	0.94	**0.98**	0.91	0.94	0.94	0.97	0.97	0.94	0.83
Waveform	0.75	0.68	0.81	0.76	0.8	0.81	0.79	0.69	0.82	0.76	0.8	**0.86**	0.65	0.76
Wine	0.74	0.93	0.73	**0.99**	0.91	0.69	0.77	0.7	0.71	0.73	0.78	0.72	0.71	0.94
Yeastgalactose	0.9	**0.95**	0.9	0.9	0.89	0.92	0.93	0.85	0.9	0.89	0.91	0.93	0.93	**0.95**
Zoo	0.74	**0.99**	0.84	0.96	0.76	0.9	0.91	0.8	0.89	0.94	0.88	0.9	0.89	0.86
Synthetic	0.73 ± 0.09	0.92 ± 0.03	0.95 ± 0.02	**0.99 ± 0.02**	0.90 ± 0.03	0.91 ± 0.04	0.94 ± 0.02	0.87 ± 0.04	0.90 ± 0.03	0.95 ± 0.02	0.93 ± 0.03	0.88 ± 0.06	0.82 ± 0.04	0.72 ± 0.05
	0.72	0.8	0.81	**0.83**	0.75	0.8	0.82	0.78	0.79	**0.83**	0.82	0.78	0.8	0.8

Table 17: Detailed results of the methods with the parameters selected using Self-Adaptive Data Shifting (SDS) over all datasets of the Type II experiments (multiple source-class inliers) with respect to ROC AUC. We visualize the distribution of these values in Figure 12(a).

ROC AUC	ABOD	Auto Enc.	GLOSH	GMM	iForest	KNN G.	SVDD	KNN L.	LOCI	LOF	LP	Parzen	SOD	DSVDD
Abalone	0.72	0.7	0.62	0.66	**0.75**	0.6	0.69	0.62	0.74	0.63	0.73	0.59	0.63	0.66
Artificial-characters	0.64	0.65	0.66	0.69	0.64	**0.71**	0.66	0.66	0.66	0.68	0.66	0.69	0.69	0.64
Balancescale	0.93	0.9	0.9	0.94	0.95	0.85	**0.97**	0.83	0.92	0.89	0.95	0.95	0.78	0.77
Car-evaluation	0.65	0.73	0.65	0.89	0.75	0.79	0.85	0.79	0.71	**0.97**	0.9	0.72	0.76	0.75
Cardiotocography	0.58	0.98	0.61	**0.99**	0.91	0.64	0.64	0.62	0.62	0.63	0.68	0.52	0.56	0.97
Cellcycle	0.79	0.77	0.69	0.75	0.7	0.78	0.76	0.56	0.7	0.76	0.77	**0.81**	0.75	0.73
Cnae	0.57	0.6	0.59	**0.78**	0.31	0.6	0.6	0.61	0.67	0.63	0.55	0.5	0.69	0.56
Dermatology	0.72	**0.93**	0.87	0.92	0.77	0.88	0.89	0.78	0.83	0.84	0.88	0.88	0.84	**0.93**
Flare	0.72	0.56	0.73	0.61	0.61	0.72	0.76	0.65	**0.79**	0.64	0.76	0.74	0.62	0.7
Glass	0.74	**0.8**	0.77	0.78	0.71	0.73	0.77	0.72	0.71	0.74	0.77	0.74	0.77	0.69
Hayes-roth	0.82	0.71	0.63	0.83	0.58	0.73	0.84	0.81	0.85	**0.87**	**0.87**	0.81	0.65	0.62
Iris	0.96	0.9	0.96	0.96	0.95	0.97	0.97	0.96	0.96	0.96	0.97	**0.98**	0.93	0.87
Led	0.68	0.68	0.53	0.69	0.56	0.59	**0.72**	0.56	0.5	0.69	0.66	0.63	0.61	0.52
Lung-cancer	**0.5**	0.44	**0.5**	**0.5**	0.38	0.44	**0.5**	**0.5**	0.47	0.44	**0.5**	**0.5**	**0.5**	0.39
Multiple-features	0.69	0.8	**0.92**	0.91	0.85	0.9	0.88	0.86	0.85	**0.92**	0.9	0.5	0.87	0.86
Optdigits	0.75	0.83	**0.99**	0.91	0.81	**0.99**	0.96	0.92	0.88	0.98	**0.99**	0.97	0.92	0.8
Sat	0.82	0.74	0.86	0.78	0.87	0.86	0.86	0.81	0.78	0.83	0.87	0.87	**0.88**	0.78
Seeds	0.91	0.88	0.94	**0.96**	0.92	0.93	0.94	0.83	0.87	0.88	0.94	0.95	0.89	0.91
Semeion	0.67	0.73	0.89	0.76	0.68	**0.9**	0.84	0.77	0.78	0.88	0.87	0.77	0.85	0.69
Soybean-small	0.82	0.89	0.99	**1**	0.93	0.91	0.97	0.85	0.86	0.97	0.99	0.9	0.95	0.84
Systhetic-control	0.71	0.76	0.97	0.78	0.78	0.97	0.91	0.98	0.91	**0.99**	0.96	0.84	0.83	0.75
Texture	0.78	0.89	0.96	0.97	0.74	0.96	0.92	0.95	0.8	**0.98**	0.8	0.88	0.95	0.82
User-knowledge	0.71	0.76	0.75	**0.81**	0.74	0.78	0.78	0.7	0.73	0.74	0.73	0.77	0.67	0.67
Vehicle	0.61	0.74	0.68	**0.79**	0.69	0.73	0.67	0.6	0.65	0.65	0.71	0.59	0.75	0.71
Vertebra-column	0.69	0.73	0.69	0.72	**0.81**	0.67	0.73	0.74	0.77	0.76	0.76	0.68	0.66	0.72
Vowels	0.71	0.8	0.92	**0.95**	0.56	**0.95**	0.84	0.91	0.91	0.82	0.88	0.9	0.94	0.81
Waveform	0.75	0.52	0.77	0.73	0.8	0.77	0.79	0.69	0.76	0.72	0.79	**0.84**	0.67	0.71
Wine	0.74	0.77	0.72	**0.99**	0.98	0.72	0.67	0.73	0.7	0.73	0.68	0.68	0.6	0.83
Yeastgalactose	0.9	0.9	0.91	0.76	0.86	0.92	0.9	0.82	0.89	0.86	0.9	**0.93**	0.9	0.89
Zoo	0.73	0.9	0.89	**0.95**	0.67	0.9	0.87	0.78	0.9	0.92	0.89	0.9	0.58	0.68
Synthetic	0.73 ± 0.09	0.90 ± 0.08	0.94 ± 0.02	0.94 ± 0.09	0.89 ± 0.08	0.89 ± 0.08	**0.95 ± 0.02**	0.86 ± 0.08	0.81 ± 0.16	0.91 ± 0.08	0.88 ± 0.13	0.89 ± 0.05	0.80 ± 0.07	0.75 ± 0.05
	0.73	0.77	0.79	**0.83**	0.74	0.8	0.8	0.75	0.77	0.8	0.81	0.77	0.76	0.74

Table 18: Detailed results of the methods with the parameters selected using Uniform Objects over all datasets of the Type II experiments (multiple source-class inliers) with respect to ROC AUC. We visualize the distribution of these values in Figure 12(b).

ROC AUC	ABOD	Auto Enc.	GLOSH	GMM	iForest	KNN G.	SVDD	KNN L.	LOCI	LOF	LP	Parzen	SOD	DSVDD
Abalone	0.72	0.65	0.59	0.68	0.74	0.6	0.63	0.61	**0.75**	0.64	0.66	0.74	0.6	0.64
Artificial-characters	0.64	0.67	0.69	**0.71**	0.64	0.7	0.51	0.65	0.66	0.68	0.61	0.65	0.68	0.65
Balancescale	0.93	0.8	0.92	0.92	**0.95**	0.94	**0.95**	0.94	0.93	0.93	**0.95**	**0.95**	0.76	0.83
Car-evaluation	0.65	0.58	0.89	0.93	0.88	0.92	0.72	0.91	0.71	**0.94**	0.62	0.72	0.78	0.75
Cardiotocography	0.58	0.8	0.65	**0.98**	0.9	0.63	0.5	0.61	0.58	0.63	0.68	0.49	0.67	0.9
Cellcycle	0.79	0.75	0.69	0.77	0.71	**0.81**	0.73	0.68	0.69	0.74	0.79	0.8	0.75	0.76
Cnae	0.57	0.61	0.72	0.77	0.41	0.7	0.56	0.66	0.61	0.72	0.53	0.5	**0.83**	0.55
Dermatology	0.72	0.86	0.91	**0.93**	0.75	0.87	0.64	0.8	0.81	0.87	0.76	0.66	0.92	0.88
Flare	0.72	0.66	0.65	0.61	0.56	0.7	0.64	0.67	**0.75**	0.7	0.62	0.73	0.73	0.64
Glass	0.74	0.7	**0.79**	0.72	0.71	0.78	0.6	0.75	0.77	0.69	0.63	0.68	0.73	0.61
Hayes-roth	0.82	0.68	0.79	0.88	0.64	0.81	0.89	0.86	0.85	0.81	**0.91**	0.82	0.72	0.59
Iris	0.96	0.95	0.91	0.96	0.9	0.96	0.91	0.9	0.96	0.95	**0.98**	0.97	0.77	0.86
Led	0.68	0.64	0.53	**0.74**	0.71	0.52	0.65	0.54	0.61	0.73	0.57	0.6	0.63	0.58
Lung-cancer	0.5	0.5	0.62	0.38	**0.78**	0.5	0.25	0.25	0.38	0.56	0.5	0.5	0.5	0.64
Multiple-features	0.69	0.81	0.9	**0.93**	0.84	0.9	0.51	0.85	0.83	0.86	0.5	0.5	0.86	0.81
Optdigits	0.75	0.85	**0.99**	0.83	0.79	0.98	0.59	0.9	0.88	0.96	**0.99**	0.89	0.92	0.78
Sat	0.82	0.73	0.86	0.73	0.86	0.85	0.65	0.81	0.81	0.83	0.87	0.8	**0.88**	0.79
Seeds	0.91	0.8	0.92	**0.96**	0.89	0.94	0.77	0.81	0.89	0.9	0.87	0.95	0.9	0.87
Semeion	0.67	0.74	**0.89**	0.73	0.77	**0.89**	0.62	0.7	0.78	0.83	0.82	0.74	0.85	0.68
Soybean-small	0.82	0.89	0.91	**1**	0.88	0.78	0.84	0.93	0.84	0.94	0.88	0.7	**1**	0.76
Systhetic-control	0.71	0.62	0.86	0.67	0.76	0.88	0.59	0.98	0.83	**0.99**	0.5	0.72	0.83	0.76
Texture	0.78	0.65	0.95	0.86	0.68	0.94	0.54	0.94	0.79	**0.97**	0.56	0.61	0.96	0.78
User-knowledge	0.71	0.66	0.75	**0.79**	0.75	0.77	0.7	0.71	0.73	0.75	0.7	0.74	0.75	0.67
Vehicle	0.61	0.59	0.65	**0.75**	0.65	0.73	0.52	0.62	0.59	0.61	0.67	0.56	0.73	0.67
Vertebra-column	0.69	0.82	0.69	0.76	0.77	0.68	**0.88**	0.68	0.76	0.78	0.74	0.68	0.62	0.7
Vowels	0.71	0.72	0.67	0.79	0.57	**0.87**	0.48	0.55	0.71	0.71	0.51	0.68	**0.87**	0.77
Waveform	0.75	0.52	0.79	0.65	0.77	0.79	0.55	0.69	0.79	0.75	0.73	**0.86**	0.66	0.72
Wine	0.74	0.78	0.66	**0.99**	0.88	0.72	0.65	0.65	0.7	0.67	0.69	0.64	0.72	0.68
Yeastgalactose	0.9	0.92	0.91	0.77	0.87	**0.93**	0.6	0.81	0.91	0.88	0.82	0.89	0.92	0.82
Zoo	0.73	0.89	0.87	**0.97**	0.78	0.9	0.42	0.79	0.84	0.84	0.8	0.76	0.67	0.5
Synthetic	0.73 ± 0.09	0.83 ± 0.13	$\mathbf{0.93 \pm 0.03}$	0.92 ± 0.09	0.88 ± 0.08	0.89 ± 0.09	0.60 ± 0.08	0.85 ± 0.08	0.79 ± 0.15	0.91 ± 0.09	0.86 ± 0.13	0.79 ± 0.12	0.81 ± 0.07	0.72 ± 0.04
	0.73	0.73	0.79	**0.81**	0.76	0.8	0.64	0.74	0.76	0.8	0.72	0.72	0.77	0.72

Table 19: Detailed results of the methods with the parameters selected using Perturbation over all datasets of the Type II experiments (multiple source-class inliers) with respect to ROC AUC. We visualize the distribution of these values in Figure 12(c).

ROC AUC	ABOD	Auto Enc.	GLOSH	GMM	iForest	KNN G.	SVDD	KNN L.	LOCI	LOF	LP	Parzen	SOD	DSVDD
Abalone	0.72	0.64	0.61	0.68	**0.74**	0.6	0.67	0.61	**0.74**	0.64	0.67	**0.74**	0.6	0.69
Artificial-characters	0.64	0.64	0.67	0.7	0.65	**0.71**	0.54	0.65	0.66	0.68	0.61	0.69	0.67	0.65
Balancescale	0.93	0.8	0.92	0.91	**0.97**	0.92	0.95	0.88	0.93	0.9	0.95	0.95	0.75	0.81
Car-evaluation	0.65	0.58	0.82	0.93	0.88	0.92	0.72	0.89	0.95	**0.97**	0.64	0.72	0.79	0.75
Cardiotocography	0.58	0.9	0.62	**0.98**	0.9	0.63	0.56	0.61	0.62	0.62	0.68	0.52	0.65	**0.98**
Cellcycle	0.79	**0.8**	0.68	0.76	0.71	0.67	0.74	0.6	0.59	0.59	0.78	0.76	0.78	0.62
Cnae	0.57	0.59	0.6	0.77	0.41	0.62	0.56	0.66	0.61	0.69	0.54	0.5	**0.84**	0.56
Dermatology	0.72	0.86	0.89	0.93	0.74	0.86	0.64	0.79	0.82	0.75	0.78	0.74	0.92	**0.95**
Flare	0.72	**0.81**	0.6	0.64	0.61	0.7	0.74	0.7	0.78	0.66	0.74	0.74	0.76	0.66
Glass	0.74	0.75	0.77	0.77	0.7	0.78	0.67	0.74	**0.79**	0.77	0.64	0.71	0.78	0.72
Hayes-roth	0.82	0.68	0.74	**0.88**	0.66	0.82	**0.88**	0.83	0.86	0.78	**0.88**	0.82	0.71	0.62
Iris	0.96	0.95	0.94	0.96	0.9	0.96	**0.97**	0.9	0.96	0.95	**0.97**	**0.97**	0.77	0.92
Led	**0.68**	0.64	0.47	0.65	0.67	0.52	0.65	0.54	0.5	0.6	0.55	0.6	0.55	0.41
Lung-cancer	0.5	0.31	0.59	0.38	**0.78**	0.5	0.25	0.5	0.38	0.75	0.5	0.5	0.5	0.6
Multiple-features	0.69	0.81	0.9	**0.93**	0.84	0.9	0.56	0.85	0.83	0.86	0.5	0.5	0.86	0.86
Optdigits	0.75	0.86	**0.99**	0.9	0.73	**0.99**	0.71	0.93	0.86	0.98	**0.99**	0.96	0.91	0.79
Sat	0.82	0.73	0.86	0.73	0.87	0.85	0.62	0.81	0.8	0.83	0.87	0.85	**0.88**	0.8
Seeds	0.91	0.8	0.88	**0.96**	0.89	0.93	0.87	0.81	0.87	0.9	0.93	0.95	0.89	0.83
Semeion	0.67	0.74	0.81	0.73	0.78	0.81	0.62	0.72	0.79	0.8	**0.82**	0.67	0.76	0.58
Soybean-small	0.82	0.92	0.93	**1**	0.88	0.86	0.84	0.92	0.9	0.89	0.88	0.87	**1**	0.83
Systhetic-control	0.71	0.63	0.88	0.67	0.76	0.89	0.6	0.98	0.86	**0.99**	0.5	0.85	0.83	0.78
Texture	0.78	0.86	0.96	0.86	0.68	0.95	0.54	0.95	0.79	**0.97**	0.56	0.68	0.96	0.77
User-knowledge	0.71	0.65	0.78	**0.81**	0.74	0.8	0.68	0.71	0.73	0.78	0.66	0.74	0.72	0.64
Vehicle	0.61	0.65	0.61	**0.75**	0.66	0.73	0.5	0.61	0.65	0.6	0.67	0.59	0.73	0.69
Vertebra-column	0.69	0.82	0.69	0.79	0.81	0.68	**0.87**	0.69	0.7	0.77	0.74	0.68	0.67	0.71
Vowels	0.71	0.74	0.74	0.86	0.57	0.88	0.5	0.67	0.67	0.79	0.53	0.7	**0.91**	0.77
Waveform	0.75	0.52	0.79	0.76	0.78	0.79	0.58	0.69	0.78	0.75	0.73	**0.85**	0.66	0.71
Wine	0.74	0.76	0.68	**0.99**	0.88	0.72	0.67	0.64	0.7	0.73	0.69	0.68	0.75	0.85
Yeastgalactose	0.9	0.91	0.91	0.77	0.86	0.92	0.7	0.73	0.87	0.87	0.82	**0.93**	0.91	**0.93**
Zoo	0.73	0.9	0.88	**0.97**	0.69	0.89	0.48	0.79	0.88	**0.97**	0.8	0.88	0.63	0.6
Synthetic	0.73 ± 0.09	0.88 ± 0.08	**0.93 ± 0.03**	0.92 ± 0.09	0.89 ± 0.08	0.89 ± 0.09	0.78 ± 0.09	0.86 ± 0.08	0.80 ± 0.16	0.91 ± 0.08	0.86 ± 0.13	0.84 ± 0.09	0.81 ± 0.07	0.75 ± 0.05
	0.73	0.74	0.77	**0.81**	0.76	0.79	0.66	0.75	0.76	0.79	0.72	0.74	0.77	0.74

Table 20: Detailed results of the methods with the parameters selected using Cross-validation over all datasets of the Type II experiments (multiple source-class inliers) with respect to AdjustedPrec@n. We visualize the distribution of these values in Figure 5(b).

AdjustedPrec@n	ABOD	Auto Enc.	GLOSH	GMM	iForest	KNN G.	SVDD	KNN L.	LOCI	LOF	LP	Parzen	SOD	DSVDD
Abalone	0.22	0.21	0.19	0.26	0.19	0.24	**0.35**	0.18	0.26	0.2	0.33	0.33	0.14	0.24
Artificial-characters	0.05	0.18	0.15	**0.25**	0.09	0.17	0.2	0.14	0.12	0.22	0.18	0.14	0.18	0.19
Balancescale	0.42	0.4	0.53	0.47	0.48	**0.55**	0.52	0.53	0.47	**0.55**	0.52	0.47	0.23	0.33
Car-evaluation	0.23	0.44	0.46	**0.69**	0.51	0.49	**0.69**	0.4	0.62	0.65	0.66	0.32	0.46	0.34
Cardiotocography	0.08	0.76	0.16	**0.89**	0.62	0.17	0.21	0.16	0.11	0.16	0.26	0.18	0.3	0.88
Cellcycle	0.33	0.36	0.39	0.2	0.27	0.42	0.25	0.33	0.2	0.34	0.23	**0.47**	0.35	0.27
Cnae	0.01	0.06	0.1	0.19	0	0.1	0.09	0.21	0.08	0.25	0.05	0.01	**0.28**	0.14
Dermatology	0.14	0.65	0.5	**0.7**	0.31	0.44	0.46	0.42	0.39	0.49	0.47	0.44	0.58	0.69
Flare	0.02	0.32	0.05	0.05	0.09	0.17	0.24	0.18	0.27	0.22	0.29	0.05	**0.33**	0.14
Glass	0.26	0.37	0.41	0.19	0.25	0.29	0.33	0.32	0.3	0.34	**0.44**	0.33	0.33	0.32
Hayes-roth	0.43	0.42	0.56	0.43	0.18	**0.71**	**0.71**	0.28	**0.71**	0.56	0.58	0.56	0.43	0.3
Iris	0.79	0.84	**0.9**	0.84	0.59	**0.9**	0.84	0.8	0.79	**0.9**	0.84	0.84	0.79	0.81
Led	0.83	0.78	0.78	0.78	**0.85**	**0.85**	0.78	0.78	0.83	0.83	0.78	0.83	0.74	0.79
Lung-cancer	**0.62**	**0.62**	**0.62**	**0.62**	**0.62**	**0.62**	**0.62**	**0.62**	**0.62**	**0.62**	**0.62**	**0.62**	**0.62**	0.48
Multiple-features	0.13	0.36	0.51	0.6	0.32	0.53	**0.62**	0.49	0.32	**0.62**	0.36	0.05	0.44	0.52
Optdigits	0.19	0.48	**0.84**	0.46	0.27	**0.84**	**0.84**	0.6	0.24	0.76	0.76	0.71	0.6	0.46
Sat	0.23	0.38	0.46	0.36	0.32	0.45	0.49	0.4	0.31	0.43	0.44	0.46	**0.51**	0.43
Seeds	0.64	0.64	0.75	0.79	0.64	0.75	**0.82**	0.64	0.64	0.79	**0.82**	0.79	0.71	0.77
Semeion	0.13	0.25	0.43	0.21	0.25	0.42	0.36	0.28	0.18	0.42	**0.44**	0.25	0.43	0.3
Soybean-small	0.59	0.84	0.88	**1**	**1**	0.59	0.75	0.71	0.71	**1**	0.88	0.62	**1**	0.85
Systhetic-control	0.27	0.72	0.78	0.44	0.29	0.81	0.77	0.87	0.67	**0.91**	0.83	0.44	0.55	0.59
Texture	0.14	0.54	0.68	0.77	0.28	0.62	0.71	0.72	0.35	**0.83**	0.54	0.37	0.66	0.59
User-knowledge	0.16	0.3	0.33	**0.37**	0.14	**0.37**	0.36	0.23	0.29	0.3	0.29	0.36	0.29	0.26
Vehicle	0.12	0.33	0.32	**0.46**	0.25	0.29	0.28	0.13	0.2	0.2	0.26	0.24	0.38	0.29
Vertebra-column	0.21	0.38	0.23	0.34	0.2	0.21	**0.5**	0.28	0.36	0.35	0.2	0.34	0.34	0.45
Vowels	0.13	0.45	0.57	0.64	0.05	0.6	**0.75**	0.44	0.46	0.58	0.71	0.73	0.59	0.38
Waveform	0.32	0.24	0.45	0.39	0.42	0.44	0.41	0.29	0.45	0.37	0.42	**0.52**	0.23	0.4
Wine	0.4	0.8	0.32	**0.88**	0.7	0.32	0.41	0.37	0.35	0.41	0.46	0.32	0.19	0.72
Yeastgalactose	0.68	0.83	0.86	0.51	0.77	0.77	0.79	0.46	**0.88**	0.74	0.86	0.77	0.83	0.78
Zoo	0.78	0.86	0.92	0.92	0.65	**1**	0.92	0.54	0.92	0.92	0.92	0.92	0.82	0.73
Synthetic	0.23 ± 0.15	0.33 ± 0.11	0.42 ± 0.12	**0.65 ± 0.14**	0.29 ± 0.12	0.38 ± 0.11	0.43 ± 0.11	0.27 ± 0.11	0.28 ± 0.11	0.46 ± 0.12	0.39 ± 0.1	0.34 ± 0.14	0.13 ± 0.09	0.11 ± 0.05
	0.32	0.49	0.5	0.52	0.39	0.5	**0.54**	0.43	0.44	0.53	0.51	0.45	0.48	0.48

Table 21: Detailed results of the methods with the parameters selected using Self-Adaptive Data Shifting (SDS) over all datasets of the Type II experiments (multiple source-class inliers) with respect to AdjustedPrec@n. We visualize the distribution of these values in Figure 13(a).

AdjustedPrec@n	ABOD	Auto Enc.	GLOSH	GMM	iForest	KNN G.	SVDD	KNN L.	LOCI	LOF	LP	Parzen	SOD	DSVDD
Abalone	0.28	0.31	0.16	0.27	**0.33**	0.18	0.28	0.19	**0.33**	0.2	0.31	0.13	0.19	0.21
Artificial-characters	0.07	0.12	0.18	**0.26**	0.11	0.2	0.2	0.19	0.11	0.23	0.2	0.15	0.2	0.17
Balancescale	0.66	0.66	0.53	0.69	0.72	0.58	**0.81**	0.56	0.68	0.5	0.74	0.72	0.43	0.41
Car-evaluation	0.23	0.31	0.19	0.6	0.24	0.5	0.69	0.5	0.42	**0.79**	0.66	0.34	0.45	0.33
Cardiotocography	0.1	0.8	0.11	**0.9**	0.62	0.17	0.17	0.13	0.08	0.16	0.25	0	0.19	0.86
Cellcycle	**0.39**	0.23	0.17	0.14	0.16	0.22	0.25	-0.02	0.22	0.34	0.3	0.36	0.22	0.24
Cnae	0.01	0.05	0.03	**0.18**	-0.1	0.02	0.07	0.05	0.1	0.08	0.02	0.01	-0.03	0.05
Dermatology	0.23	0.67	0.5	**0.71**	0.4	0.54	0.62	0.26	0.46	0.35	0.62	0.55	0.46	**0.71**
Flare	0.03	0.1	0.05	0.02	0.11	0.12	0.24	0.11	**0.26**	0.09	0.21	0.05	0.02	0.14
Glass	0.26	**0.39**	**0.39**	0.28	0.23	0.21	0.31	0.35	0.16	0.35	0.33	0.31	0.33	0.19
Hayes-roth	0.43	0.43	0.15	0.58	0.16	0.56	**0.71**	**0.71**	0.56	**0.71**	0.58	0.43	0.42	0.21
Iris	0.79	0.79	0.84	0.79	0.79	0.84	**0.89**	0.84	0.79	0.84	0.84	0.79	0.79	0.7
Led	**0.83**	0.78	0.74	0.78	0.78	0.74	**0.83**	0.74	0.74	0.78	**0.83**	**0.83**	0.78	0.79
Lung-cancer	**0.62**	**0.62**	**0.62**	**0.62**	**0.62**	**0.62**	**0.62**	**0.62**	**0.62**	**0.62**	**0.62**	**0.62**	**0.62**	0.55
Multiple-features	0.14	0.27	0.51	0.45	0.31	**0.53**	0.51	0.39	0.28	0.52	0.35	0.05	0.38	0.43
Optdigits	0.19	0.35	0.83	0.46	0.27	0.84	0.7	0.59	0.22	0.76	**0.85**	0.72	0.57	0.32
Sat	0.29	0.37	0.5	0.42	0.48	0.5	**0.57**	0.47	0.38	0.5	0.54	0.52	0.56	0.41
Seeds	0.64	0.57	0.71	0.75	0.71	0.75	**0.82**	0.5	0.57	0.57	0.68	0.79	0.61	0.66
Semeion	0.13	0.18	0.43	0.22	0.12	**0.44**	0.3	0.22	0.15	0.39	0.35	0.21	0.39	0.17
Soybean-small	0.59	0.71	**1**	**1**	0.84	0.75	0.88	0.59	0.59	0.88	0.88	0.75	0.84	0.64
Systhetic-control	0.27	0.39	0.79	0.41	0.29	0.82	0.74	0.88	0.59	**0.92**	0.77	0.26	0.6	0.34
Texture	0.14	0.47	0.61	0.75	0.25	0.62	0.67	0.73	0.13	**0.82**	0.44	0.38	0.65	0.39
User-knowledge	0.16	0.31	0.22	**0.39**	0.16	0.31	0.3	0.19	0.23	0.26	0.27	0.27	0.28	0.21
Vehicle	0.13	0.31	0.24	**0.45**	0.26	0.29	0.24	0.2	0.14	0.17	0.28	0.13	0.33	0.27
Vertebra-column	0.37	0.47	0.37	0.29	**0.52**	0.37	0.35	0.29	0.43	0.44	0.3	0.37	0.29	0.38
Vowels	0.14	0.34	0.58	**0.68**	0.08	0.61	0.58	0.45	0.3	0.38	0.52	**0.68**	0.58	0.32
Waveform	0.32	0.04	0.4	0.34	0.41	0.4	0.42	0.28	0.36	0.34	0.42	**0.49**	0.24	0.32
Wine	0.4	0.55	0.4	**0.91**	0.87	0.4	0.32	0.31	0.35	0.36	0.36	0.45	0.19	0.47
Yeastgalactose	0.68	0.69	**0.86**	0.23	0.6	0.77	**0.86**	0.54	0.69	0.73	**0.86**	0.74	0.78	0.65
Zoo	0.78	0.59	0.92	0.82	0.78	**1**	0.92	0.82	0.92	**1**	0.92	0.92	0.54	0.47
Synthetic	0.21 ± 0.14	0.34 ± 0.12	0.38 ± 0.11	**0.45 ± 0.15**	0.29 ± 0.12	0.34 ± 0.1	0.44 ± 0.1	0.24 ± 0.12	0.21 ± 0.14	0.35 ± 0.12	0.33 ± 0.15	0.33 ± 0.14	0.13 ± 0.09	0.12 ± 0.04
	0.34	0.43	0.47	0.51	0.4	0.5	**0.53**	0.42	0.4	0.5	0.51	0.43	0.43	0.4

Table 22: Detailed results of the methods with the parameters selected using Uniform Objects over all datasets of the Type II experiments (multiple source-class inliers) with respect to AdjustedPrec@n. We visualize the distribution of these values in Figure 13(b).

AdjustedPrec@n	ABOD	Auto Enc.	GLOSH	GMM	iForest	KNN G.	SVDD	KNN L.	LOCI	LOF	LP	Parzen	SOD	DSVDD
Abalone	0.28	0.23	0.09	0.25	0.31	0.18	0.23	0.2	**0.39**	0.21	0.22	0.32	0.12	0.2
Artificial-characters	0.07	0.16	0.16	**0.28**	0.11	0.18	0.03	0.17	0.11	0.15	0.16	0.12	0.18	0.17
Balancescale	0.66	0.46	0.72	0.68	0.74	**0.79**	0.76	0.68	0.72	0.72	0.74	0.72	0.43	0.52
Car-evaluation	0.23	0.21	0.46	0.68	0.5	0.65	0.35	0.62	0.42	**0.75**	0.25	0.34	0.45	0.32
Cardiotocography	0.1	0.44	0.13	**0.83**	0.53	0.13	-0.04	0.17	0.07	0.15	0.25	-0.02	0.29	0.6
Cellcycle	**0.39**	0.23	0.18	0.2	0.25	0.33	0.33	0.08	0.1	0.22	0.23	0.3	0.31	0.23
Cnae	0.01	0.07	0.1	0.18	0	0.11	-0.01	0.08	0.04	0.14	0	0.01	**0.25**	0.03
Dermatology	0.23	0.57	0.6	**0.78**	0.35	0.53	0.14	0.36	0.4	0.46	0.4	0.14	0.67	0.61
Flare	0.03	0.12	0.07	0	0.07	0.08	0.2	0.15	0.26	0.05	0.16	0.05	**0.27**	0.12
Glass	0.26	0.24	0.35	0.3	0.25	0.26	0.26	0.34	0.31	0.26	**0.36**	0.25	0.34	0.08
Hayes-roth	0.43	0.42	0.56	0.71	0.18	0.56	**0.85**	0.71	0.71	0.56	0.72	0.56	0.3	0.19
Iris	0.79	0.79	0.74	0.84	0.69	0.84	0.69	0.74	**0.85**	0.79	0.84	0.79	0.52	0.65
Led	**0.83**	**0.83**	0.74	0.78	**0.83**	0.78	0.78	0.78	0.74	0.78	0.78	**0.83**	0.78	0.81
Lung-cancer	**0.62**	**0.62**	**0.62**	**0.62**	**0.62**	**0.62**	**0.62**	**0.62**	**0.62**	**0.62**	**0.62**	**0.62**	**0.62**	**0.62**
Multiple-features	0.14	0.31	0.5	**0.52**	0.35	**0.52**	0.01	0.43	0.29	0.43	0.05	0.05	0.39	0.37
Optdigits	0.19	0.37	0.84	0.31	0.24	0.81	0.11	0.5	0.23	0.71	**0.85**	0.42	0.58	0.3
Sat	0.29	0.34	0.5	0.35	0.47	0.48	0.26	0.47	0.31	0.5	0.54	0.26	**0.56**	0.39
Seeds	0.64	0.46	0.75	**0.79**	0.68	0.75	0.5	0.54	0.64	0.57	0.57	**0.79**	0.64	0.59
Semeion	0.13	0.18	**0.43**	0.19	0.22	0.4	0.11	0.14	0.15	0.32	0.33	0.19	0.41	0.15
Soybean-small	0.59	0.71	0.75	**1**	0.75	0.3	0.59	0.75	0.59	0.88	0.59	0.43	**1**	0.56
Systhetic-control	0.27	0.07	0.43	0.2	0.27	0.43	0.1	0.86	0.37	**0.93**	-0.02	0.31	0.54	0.36
Texture	0.14	0.17	0.64	0.45	0.21	0.55	0.07	0.71	0.1	**0.78**	0.13	0.07	0.65	0.3
User-knowledge	0.16	0.25	0.21	**0.35**	0.15	0.28	0.23	0.25	0.22	0.31	0.21	0.23	0.25	0.22
Vehicle	0.13	0.12	0.22	**0.36**	0.16	0.31	0.04	0.12	0.02	0.15	0.24	0.08	0.3	0.24
Vertebra-column	0.37	0.47	0.37	0.42	0.44	0.37	**0.56**	0.17	0.46	0.47	0.35	0.37	0.24	0.38
Vowels	0.14	0.23	0.15	0.34	0.07	**0.49**	0.05	0.04	0.15	0.22	0.06	0.32	0.47	0.3
Waveform	0.32	0.04	0.42	0.21	0.36	0.42	0.06	0.28	0.38	0.37	0.34	**0.5**	0.23	0.33
Wine	0.4	0.59	0.24	**0.96**	0.7	0.4	0.4	0.28	0.35	0.14	0.4	0.4	0.24	0.26
Yeastgalactose	0.68	0.74	**0.83**	0.26	0.65	0.77	0.38	0.41	0.71	0.78	0.63	0.65	0.81	0.44
Zoo	0.78	0.63	0.86	0.82	0.86	**0.93**	0.51	0.85	0.76	0.9	0.85	0.82	0.64	0.37
Synthetic	0.21 ± 0.14	0.28 ± 0.17	0.36 ± 0.14	**0.40 ± 0.16**	0.28 ± 0.14	0.34 ± 0.1	0.09 ± 0.07	0.22 ± 0.12	0.21 ± 0.15	0.35 ± 0.11	0.28 ± 0.12	0.27 ± 0.15	0.16 ± 0.11	0.1 ± 0.05
	0.34	0.37	0.46	**0.49**	0.4	0.48	0.31	0.42	0.38	0.48	0.4	0.36	0.45	0.36

Table 23: Detailed results of the methods with the parameters selected using Perturbation over all datasets of the Type II experiments (multiple source-class inliers) with respect to AdjustedPrec@n. We visualize the distribution of these values in Figure 13(c).

AdjustedPrec@n	ABOD	Auto Enc.	GLOSH	GMM	iForest	KNN G.	SVDD	KNN L.	LOCI	LOF	LP	Parzen	SOD	DSVDD
Abalone	0.28	0.2	0.14	0.25	0.31	0.16	0.2	0.2	**0.38**	0.21	0.16	0.32	0.12	0.25
Artificial-characters	0.07	0.12	0.18	**0.28**	0.11	0.2	0.08	0.17	0.11	0.22	0.16	0.15	0.21	0.18
Balancescale	0.66	0.46	0.72	0.64	**0.77**	0.69	**0.77**	0.55	0.74	0.63	0.74	0.72	0.4	0.49
Car-evaluation	0.23	0.22	0.48	0.68	0.5	0.65	0.37	0.6	0.62	**0.79**	0.25	0.34	0.53	0.32
Cardiotocography	0.1	0.58	0.14	0.83	0.55	0.17	0.1	0.07	0.1	0.15	0.25	0	0.28	**0.88**
Cellcycle	0.39	0.27	0.15	0.24	0.18	0.04	0.33	0.02	0.12	0.11	0.38	0.25	**0.4**	0.14
Cnae	0.01	0.04	0.04	0.18	0.01	0.07	-0.01	0.06	0.04	0.11	0	0.01	**0.26**	0.04
Dermatology	0.23	0.57	0.55	**0.78**	0.29	0.5	0.23	0.33	0.46	0.29	0.46	0.35	0.65	0.73
Flare	0.03	0.29	0.01	-0.02	0.07	0.08	**0.35**	0.23	0.15	0.07	0.23	0.05	0.29	0.14
Glass	0.26	0.33	0.36	0.31	0.22	0.29	**0.37**	0.33	0.35	0.21	0.35	0.24	0.36	0.25
Hayes-roth	0.43	0.42	0.56	**0.71**	0.18	0.56	**0.71**	0.58	**0.71**	0.56	0.58	0.56	0.3	0.16
Iris	0.79	0.79	0.74	0.84	0.69	0.84	0.84	0.74	**0.85**	0.79	0.84	0.79	0.52	0.68
Led	**0.83**	**0.83**	0.74	0.78	**0.83**	0.78	0.78	0.78	0.74	0.78	0.78	**0.83**	0.78	0.78
Lung-cancer	0.62	0.62	0.62	0.62	0.62	0.62	0.62	0.62	0.62	0.62	0.62	0.62	0.62	**0.7**
Multiple-features	0.14	0.31	0.5	**0.52**	0.32	**0.52**	0.02	0.41	0.29	0.43	0.05	0.05	0.34	0.45
Optdigits	0.19	0.39	0.83	0.41	0.17	0.84	0.29	0.59	0.21	0.76	**0.85**	0.69	0.54	0.31
Sat	0.29	0.33	0.5	0.35	0.47	0.48	0.24	0.47	0.32	0.5	**0.54**	0.42	**0.54**	0.41
Seeds	0.64	0.46	0.68	**0.79**	0.68	0.75	0.64	0.54	0.57	0.57	0.68	**0.79**	0.64	0.53
Semeion	0.13	0.16	0.25	0.19	0.24	0.26	0.12	0.14	0.2	0.31	**0.33**	0.14	0.08	0.05
Soybean-small	0.59	0.71	0.88	**1**	0.75	0.59	0.59	0.75	0.59	0.75	0.59	0.59	**1**	0.59
Systhetic-control	0.27	0.07	0.54	0.2	0.26	0.52	0.11	0.86	0.44	**0.93**	-0.02	0.31	0.55	0.38
Texture	0.14	0.38	0.68	0.45	0.19	0.58	0.07	0.72	0.1	**0.76**	0.13	0.12	0.65	0.27
User-knowledge	0.16	0.24	0.25	**0.39**	0.16	0.31	0.15	0.23	0.23	0.32	0.21	0.22	0.21	0.21
Vehicle	0.13	0.19	0.2	**0.36**	0.17	0.29	-0.02	0.12	0.12	0.16	0.24	0.13	0.3	0.24
Vertebra-column	0.37	0.47	0.37	0.47	**0.54**	0.37	0.51	0.28	0.3	0.44	0.35	0.37	0.24	0.36
Vowels	0.14	0.29	0.19	0.41	0.08	0.49	0.09	0.17	0.13	0.29	0.15	0.32	**0.5**	0.29
Waveform	0.32	0.04	0.43	0.39	0.37	0.43	0.1	0.28	0.38	0.37	0.34	**0.51**	0.23	0.33
Wine	0.4	0.4	0.28	**0.96**	0.7	0.4	0.32	0.23	0.35	0.36	0.4	0.45	0.19	0.5
Yeastgalactose	0.68	0.61	**0.86**	0.26	0.55	0.77	0.53	0.15	0.81	0.73	0.63	0.77	0.72	0.68
Zoo	0.78	0.63	0.86	0.82	0.76	**0.92**	0.51	0.85	0.82	0.82	0.85	**0.92**	0.86	0.45
Synthetic	0.21 ± 0.14	0.31 ± 0.14	0.37 ± 0.14	**0.40 ± 0.16**	0.29 ± 0.13	0.34 ± 0.1	0.22 ± 0.11	0.24 ± 0.11	0.21 ± 0.15	0.35 ± 0.11	0.28 ± 0.12	0.29 ± 0.13	0.15 ± 0.09	0.13 ± 0.04
	0.34	0.38	0.46	**0.5**	0.39	0.47	0.33	0.4	0.4	0.47	0.4	0.4	0.44	0.39

Table 24: Detailed results of the methods with the parameters selected using Cross-validation over all datasets of the Type II experiments (multiple source-class inliers) with respect to MCC. We visualize the distribution of these values in Figure 5(c).

MCC	ABOD	Auto Enc.	GLOSH	GMM	iForest	KNN G.	SVDD	KNN L.	LOCI	LOF	LP	Parzen	SOD	DSVDD
Abalone	0.18	0.12	0.2	0.23	0.21	0.23	0.2	0.21	**0.26**	0.24	0.2	0.17	0.15	0.23
Artificial-characters	0.09	0.22	0.17	**0.29**	0.13	0.21	0.13	0.15	0.17	0.23	0.13	0.17	0.2	0.2
Balancescale	0.43	0.43	0.47	0.46	0.5	0.5	0.29	**0.55**	0.46	0.49	0.35	0.48	0.26	0.35
Car-evaluation	0.23	0.38	0.44	**0.66**	0.54	0.64	0.13	0.51	0.64	0.58	0.31	0.33	0.42	0.35
Cardiotocography	0.11	0.73	0.14	0.76	0.51	0.11	0.11	0.18	0.11	0.14	0.13	0.01	0.24	**0.87**
Cellcycle	0.28	0.31	0.28	0.32	0.21	0.26	0.26	0.28	0.24	0.31	0.3	**0.52**	0.26	0.3
Cnae	0.02	0.08	0.15	0.29	0	0.13	0.02	0.23	0.1	0.25	0.06	0	**0.36**	0.15
Dermatology	0.19	0.59	0.54	0.61	0.36	0.41	0.06	0.41	0.38	0.48	0.07	0.15	0.6	**0.63**
Flare	0.01	**0.38**	0.01	0.07	0.15	0.1	0.32	0.18	0.22	0.19	0.35	0.23	0.34	0.15
Glass	0.27	0.29	0.24	**0.51**	0.25	0.28	0.22	0.19	0.26	0.19	0.29	0	0.42	0.31
Hayes-roth	0.44	0.23	0.5	0.43	0.17	0.5	0.64	0.25	0.5	0.44	**0.65**	0.44	0.44	0.21
Iris	0.8	0.78	0.81	0.67	0.63	0.85	0.77	0.79	0.82	**0.87**	0.75	0.66	0.82	0.82
Led	0.06	-0.02	0.04	0.11	0.09	0	0.03	0	0.11	0.02	0.09	**0.14**	0.07	0.13
Lung-cancer	0.32	0	0.19	0	0.32	0.32	-0.2	0	0	0.32	0	**0.33**	0.12	0.16
Multiple-features	0.22	0.37	0.6	0.22	0.38	0.56	0.28	0.49	0.47	**0.61**	0	0	0.52	0.48
Optdigits	0.24	0.52	0.75	0.51	0.32	**0.76**	0.07	0.58	0.46	0.73	0.04	0.69	0.6	0.45
Sat	0.23	0.38	0.51	0.38	0.32	0.44	0.21	0.39	0.32	0.41	0.22	0.41	**0.52**	0.43
Seeds	0.59	0.46	0.76	**0.77**	0.58	0.75	0.49	0.65	0.64	0.73	0.61	0.65	0.71	0.75
Semeion	0.17	0.27	**0.47**	0.04	0.31	0.45	0.01	0.32	0.23	0.46	0	0.2	0.46	0.28
Soybean-small	0.59	0.62	0.79	0.35	**0.94**	0.73	0.46	0.81	0.79	0.89	0.21	0.63	**0.94**	0.24
Systhetic-control	0.31	0.63	0.8	0.29	0.34	0.79	0.32	0.82	0.69	**0.84**	0	0.31	0.59	0.59
Texture	0.26	0.59	0.67	**0.76**	0.34	0.63	0.44	0.68	0.46	**0.76**	0.52	0.42	0.64	0.54
User-knowledge	0.2	0.34	0.25	**0.44**	0.24	0.32	0.2	0.21	0.25	0.34	0.29	0.08	0.21	0.26
Vehicle	0.14	**0.34**	0.33	**0.34**	0.21	0.22	0.05	0.12	0.17	0.28	0.16	0.08	0.33	0.29
Vertebra-column	0.21	0.4	0.24	0.31	0.21	0.26	0.34	0.29	0.33	0.37	0.18	0.21	0.34	**0.44**
Vowels	0.2	0.52	0.57	0.51	0.12	**0.61**	0.16	0.45	0.6	0.55	0.27	0.16	0.56	0.39
Waveform	0.32	0.21	0.45	0.39	0.39	0.45	0.32	0.31	0.43	0.39	0.27	**0.52**	0.21	0.43
Wine	0.48	0.19	0.41	0.56	0.6	0.41	0.46	0.46	0.36	0.41	0.24	0.46	0.29	**0.61**
Yeastgalactose	0.7	0.55	**0.76**	0.26	0.69	0.72	0.62	0.56	0.71	0.68	0.71	0.69	0.72	0.64
Zoo	0.35	0.17	0.45	0.11	0.21	0.47	0.17	0.47	0.52	0.53	0.33	0.55	**0.63**	0.24
Synthetic	0.18 ± 0.13	0.34 ± 0.08	0.40 ± 0.08	**0.60 ± 0.18**	0.29 ± 0.11	0.33 ± 0.09	0.23 ± 0.06	0.27 ± 0.09	0.28 ± 0.06	0.42 ± 0.08	0.28 ± 0.09	0.29 ± 0.09	0.17 ± 0.07	0.12 ± 0.04
	0.29	0.37	0.43	0.39	0.34	0.44	0.25	0.38	0.39	**0.46**	0.26	0.32	0.43	0.4

Table 25: Detailed results of the methods with the parameters selected using Self-Adaptive Data Shifting (SDS) over all datasets of the Type II experiments (multiple source-class inliers) with respect to MCC. We visualize the distribution of these values in Figure 14(a).

MCC	ABOD	Auto Enc.	GLOSH	GMM	iForest	KNN G.	SVDD	KNN L.	LOCI	LOF	LP	Parzen	SOD	DSVDD
Abalone	0.25	0.3	0.17	0.3	0.34	0.22	0.29	0.21	**0.36**	0.23	0.31	0.07	0.24	0.2
Artificial-characters	0.1	0.14	0.19	**0.26**	0.12	0.2	0.18	0.18	0.17	0.23	0.17	0.16	0.2	0.16
Balancescale	0.69	0.64	**0.71**	0.7	0.6	**0.71**	0.63	**0.71**	0.66	0.48	0.65	**0.71**	0.52	0.41
Car-evaluation	0.24	0.31	0.38	0.59	0.13	**0.71**	0.18	**0.71**	0.24	0.7	0.31	0.31	0.4	0.31
Cardiotocography	0.1	0.74	0.07	0.77	0.53	0.13	0.16	0.15	0.08	0.11	0.14	0.03	0.04	**0.78**
Cellcycle	0.24	0.33	0.13	0.22	0.17	0.16	0.32	0.09	0.26	0.3	**0.37**	0.31	0.21	0.26
Cnae	0.03	0.03	0.05	**0.26**	-0.09	0.05	0.1	0.08	0.14	0.11	0	0	0.15	0.06
Dermatology	0.15	**0.64**	0.46	0.51	0.35	0.52	0.3	0.26	0.41	0.3	0.33	0.21	0.39	0.48
Flare	0.05	0.07	-0.05	0.05	0.07	0.06	0.28	0.08	**0.29**	0.19	**0.29**	0.1	0.13	0.15
Glass	0.28	0.25	0.3	0.33	0.28	0.26	**0.34**	0.26	0.1	0.24	0.31	0.29	0.33	0.21
Hayes-roth	0.44	0.44	0.37	0.45	-0.02	**0.5**	0.04	0.41	**0.5**	0.35	0.31	0.44	0.41	0.13
Iris	0.7	0.5	0.69	0.45	0.73	0.74	0.51	**0.78**	0.72	0.69	0.64	0.68	0.74	0.45
Led	0.07	0.09	-0.01	0.02	0.02	-0.01	0.03	0.04	0	0.04	0.05	0.08	**0.17**	-0.01
Lung-cancer	0	0	0.12	0	-0.08	0.12	-0.32	**0.32**	0	**0.32**	0	-0.08	0.12	-0.17
Multiple-features	0.18	0.27	**0.51**	0.24	0.31	0.47	0.4	0.4	0.38	0.5	0	0	0.43	0.34
Optdigits	0.23	0.34	**0.76**	0.45	0.32	**0.76**	0.37	0.5	0.36	0.73	0.09	0.63	0.48	0.31
Sat	0.15	0.32	0.47	0.39	0.43	0.46	0.45	0.4	0.33	0.43	0.38	0.46	**0.48**	0.35
Seeds	0.62	0.52	**0.67**	0.62	0.64	0.63	0.54	0.54	0.5	0.6	0.54	0.58	0.56	0.61
Semeion	0	0	0.13	0	0.13	**0.45**	0.01	0.21	0	0.33	0	0.1	0.4	0.15
Soybean-small	0.45	0.6	**0.95**	0.35	0.74	0.75	0	0.44	0.67	0.85	0	0.64	0.84	0.31
Systhetic-control	0.26	0.36	0.58	0.22	0.3	0.58	0.22	0.76	0.53	**0.85**	0	0.27	0.53	0.29
Texture	0.21	0.44	0.59	**0.67**	0.25	0.55	0.41	0.52	0.22	0.62	0.4	0.35	0.53	0.33
User-knowledge	0.14	0.2	0.22	**0.39**	0.25	0.31	0.24	0.28	0.24	0.3	0.29	0.24	0.16	0.19
Vehicle	0.11	0.25	0.25	**0.42**	0.27	0.24	0.24	0.18	0.14	0.17	0.26	0.06	0.32	0.27
Vertebra-column	0.35	0.43	0.35	0.3	**0.49**	0.33	0.28	0.31	0.42	0.4	0.27	0.34	0.26	0.26
Vowels	0.16	0.25	0.49	0.47	0.07	**0.6**	0.24	0.48	0.45	0.35	0.3	0.4	0.5	0.3
Waveform	0.3	0.01	0.41	0.29	0.37	0.41	0.29	0.29	0.19	0.33	0.34	**0.44**	0.23	0.34
Wine	0.44	0.48	0.4	0.69	**0.76**	0.43	0.27	0.31	0.4	0.31	0.12	0.47	0.18	0.47
Yeastgalactose	0.58	0.44	0.59	0.38	0.51	**0.6**	0.38	0.43	0.46	0.51	0.44	0.42	**0.6**	0.33
Zoo	0.28	0.58	0.5	0.42	0.21	0.53	0.19	0.5	0.34	**0.67**	0.3	0.36	0.21	0.17
Synthetic	0.14 ± 0.11	0.30 ± 0.1	**0.36 ± 0.1**	0.32 ± 0.14	0.27 ± 0.08	0.31 ± 0.09	0.21 ± 0.04	0.24 ± 0.07	0.23 ± 0.13	0.32 ± 0.09	0.22 ± 0.11	0.27 ± 0.08	0.16 ± 0.07	0.14 ± 0.04
	0.26	0.33	0.38	0.37	0.31	**0.42**	0.25	0.36	0.32	0.41	0.25	0.3	0.36	0.28

Table 26: Detailed results of the methods with the parameters selected using Uniform Objects over all datasets of the Type II experiments (multiple source-class inliers) with respect to MCC. We visualize the distribution of these values in Figure 14(b).

MCC	ABOD	Auto Enc.	GLOSH	GMM	iForest	KNN G.	SVDD	KNN L.	LOCI	LOF	LP	Parzen	SOD	DSVDD
Abalone	0.25	0.03	0.12	0.05	0.23	0.02	0.2	0.24	**0.35**	0.23	0.22	0.08	0.13	0.05
Artificial-characters	0.04	0.05	0.08	**0.25**	0.1	0.11	0.1	0.18	0.05	0.11	0.17	0.09	0.17	0.11
Balancescale	0.73	0.42	0.76	0.65	0.74	**0.8**	0.75	0.69	0.69	0.69	0.77	0.72	0.47	0.51
Car-evaluation	0.24	0.21	0.61	0.72	0.48	**0.75**	0.38	0.7	0.43	0.64	0.29	0.32	0.43	0.31
Cardiotocography	0.01	**0.43**	0	0.32	0.08	0.01	-0.01	0.01	0.06	-0.02	0.14	0.01	0.01	0.35
Cellcycle	0.24	0.1	0.19	0.01	0.15	0.25	0.29	0	0.23	0.19	**0.35**	0.33	0.15	0.31
Cnae	0	0.01	0.02	**0.18**	0	0.02	-0.01	0.03	0.02	0.02	0	0	0.03	0.04
Dermatology	-0.01	0.53	-0.01	**0.71**	0.26	-0.01	-0.04	0.12	0.16	0.16	0.38	-0.01	0.34	0.52
Flare	-0.02	-0.01	0	0.1	0.02	-0.02	0.16	0.16	**0.18**	-0.03	**0.18**	0.01	0.15	0.07
Glass	0.2	0.24	0.25	0.11	0.25	0.14	0.24	0.19	0.21	**0.3**	0.19	0.09	0.25	0.04
Hayes-roth	0.51	0.47	0.45	0.38	0.12	0.52	0.54	**0.62**	0.58	0.35	0.59	0.4	0.41	0.09
Iris	0.67	**0.87**	0.66	0.76	0.64	0.77	0.54	0.76	0.69	0.66	0.79	0.82	0.59	0.57
Led	0.09	**0.17**	0.02	0.15	0.15	0.05	0.16	0.03	0.02	0.07	0.05	0.08	-0.07	0.05
Lung-cancer	0	0	0	0	**0.32**	0	-0.2	0	0.19	0	0	0	0	0.15
Multiple-features	0	0.31	0.01	**0.4**	0	0.03	0.06	0.07	0.24	0.08	0	0	0.07	0.24
Optdigits	0.03	0.01	0.29	0.02	0.01	0.18	0.08	**0.49**	0.08	0.19	0.09	0.07	0.26	0.15
Sat	0.18	0.18	0.21	0.2	0.25	0.2	0.32	0.23	0.15	0.24	**0.38**	0.18	0.23	0.2
Seeds	0.47	0.36	0.59	**0.71**	0.54	0.55	0.46	0.4	0.61	0.41	0.62	0.65	0.42	0.41
Semeion	0.1	0.03	0.04	0.15	0.03	0.01	0.12	0.12	0.08	0.08	0.12	0.09	0.13	**0.16**
Soybean-small	0.45	0.6	0.75	0.35	0.59	0.55	0.43	0.83	0.31	0.88	0.37	0.45	**0.94**	0.32
Systhetic-control	0.18	0.16	0.23	0.22	0.21	0.25	0.18	0.62	0.29	**0.71**	0	0.24	0.28	0.29
Texture	0.01	0.04	0.13	0.17	0.22	0.11	0.09	0.28	0.09	**0.3**	0.14	0.01	0.05	0.11
User-knowledge	0.14	0.16	0.21	**0.3**	0.27	0.27	0.2	0.22	0.2	**0.3**	0.18	0.22	0.28	0.2
Vehicle	0.03	0	0.03	0.02	-0.02	0.03	0.1	0.05	-0.04	0.03	**0.25**	0.03	0.08	0.11
Vertebra-column	0.32	0.36	0.37	0.38	0.33	0.44	**0.48**	0.25	**0.48**	0.44	0.3	0.31	0.28	0.4
Vowels	0.13	0.14	0.13	0.12	0.06	**0.5**	0.08	0.05	0.07	0.15	0.09	0.16	0.17	0.27
Waveform	0.01	-0.01	0.18	0.06	0.15	0.19	0.01	0.15	0.14	0.16	0.22	0.08	0.16	**0.37**
Wine	0.3	0.56	0.24	**0.8**	0.07	0.3	0.42	0.28	0.38	0.12	0.29	0.3	0.07	0.23
Yeastgalactose	0.43	0.51	0.31	0.41	0.35	0.24	0.43	0.19	0.37	0.56	**0.59**	0.25	0.19	0.36
Zoo	0.13	0.58	0.29	0.54	0.43	0.13	0.04	**0.64**	0.46	0.25	0.27	0.22	0.25	0.01
Synthetic	0.12 ± 0.15	0.09 ± 0.14	0.13 ± 0.16	0.25 ± 0.16	0.08 ± 0.14	0.16 ± 0.18	0.07 ± 0.09	0.06 ± 0.1	0.23 ± 0.13	0.1 ± 0.13	**0.31** ± **0.14**	0.14 ± 0.16	0.01 ± 0.05	0.03 ± 0.04
	0.2	0.25	0.24	**0.31**	0.23	0.25	0.22	0.29	0.26	0.28	0.27	0.21	0.23	0.23

Table 27: Detailed results of the methods with the parameters selected using Perturbation over all datasets of the Type II experiments (multiple source-class inliers) with respect to MCC. We visualize the distribution of these values in Figure 14(c).

	ABOD	Auto Enc.	GLOSH	GMM	iForest	KNN G.	SVDD	KNN L.	LOCI	LOF	LP	Parzen	SOD	DSVDD
Abalone	0.25	0.08	0.15	0.09	0.23	0.17	0.28	0.24	**0.35**	0.23	0.27	0.08	0.13	0.09
Artificial-characters	0.04	0.09	0.16	**0.26**	0.1	0.19	0.13	0.18	0.05	0.23	0.17	0.12	0.2	0.18
Balancescale	0.7	0.45	0.76	0.63	0.74	0.76	0.75	0.66	0.69	0.65	**0.77**	0.64	0.54	0.48
Car-evaluation	0.24	0.22	0.6	0.72	0.48	**0.75**	0.43	0.72	0.65	0.73	0.29	0.35	0.54	0.33
Cardiotocography	0.07	0.67	0.08	0.32	0.09	0.09	0.12	0.08	0.06	0.14	0.14	-0.01	0.01	**0.76**
Cellcycle	0	0.1	0	0.12	0.17	0	0.29	0.13	0	0.08	**0.32**	0.23	0.1	0.2
Cnae	0	0.03	0.02	0.18	0	0.02	-0.01	0.03	0.02	0.08	0	0	**0.2**	0.05
Dermatology	0.18	0.53	0.49	**0.71**	0.21	0.38	0.17	0.25	0.18	0.27	0.37	0.19	0.4	0.65
Flare	0.08	0.26	0.02	0.04	0.06	-0.02	**0.33**	0.2	0.1	0.01	0.24	0	0.24	0.14
Glass	0.27	0.27	0.17	0.25	0.23	0.2	0.22	0.15	0.2	0.18	0.26	0.19	**0.3**	0.23
Hayes-roth	0.35	0.47	0.5	0.34	0.12	0.5	0.54	0.48	0.5	0.39	**0.64**	0.44	0.48	0.18
Iris	0.67	**0.87**	0.69	0.74	0.64	0.8	0.76	0.76	0.69	0.69	0.76	0.74	0.61	0.62
Led	0.07	**0.17**	-0.04	0.06	0.15	0.05	0.16	0.01	0	-0.02	0.05	-0.03	0.01	-0.03
Lung-cancer	**0.32**	0	0.19	0	**0.32**	0.19	-0.2	0	**0.32**	0	0	0	0.12	0.23
Multiple-features	0.05	0.31	0.01	0.4	0	0.03	0.04	0.09	0.24	0.08	0	0	0.15	**0.43**
Optdigits	0.23	0.29	**0.75**	0.06	0.06	**0.75**	0.29	0.57	0.17	0.72	0.08	0.63	0.53	0.32
Sat	0.12	0.17	0.21	0.2	0.31	0.2	0.3	0.23	0.24	0.24	**0.38**	0.17	0.28	0.21
Seeds	0.65	0.36	0.59	0.71	0.52	**0.77**	0.59	0.4	0.58	0.55	0.65	0.65	0.47	0.47
Semeion	0.14	0.18	0.27	0.18	0.25	0.28	0.12	0.17	0.23	**0.3**	0.12	0.14	0.12	0.03
Soybean-small	0.45	0.58	0.68	0.35	0.69	0.55	0.4	0.77	0.79	0.59	0.35	0.51	**0.88**	0.28
Systhetic-control	0.18	0.16	0.37	0.22	0.19	0.37	0.17	0.62	0.5	**0.71**	0	0.24	0.35	0.32
Texture	0.12	0.05	0.13	0.17	0.21	0.11	0.09	0.3	0.09	**0.31**	0.14	0.01	0.05	0.09
User-knowledge	0.19	0.16	0.27	**0.38**	0.2	0.28	0.16	0.24	0.17	0.27	0.11	0.19	0.19	0.17
Vehicle	-0.01	0.08	0.1	0.02	0.01	0.03	0.06	0.12	0.01	0.06	**0.25**	0.04	0.08	0.11
Vertebra-column	0.32	0.36	0.36	0.38	**0.51**	0.44	0.48	0.23	0.29	0.42	0.3	0.38	0.24	0.34
Vowels	0.13	0.18	0.18	0.25	0.06	**0.5**	0.09	0.13	0.07	0.28	0.14	0.15	0.23	0.27
Waveform	0.13	-0.01	0.19	0.26	0.23	0.2	0.05	0.15	0.14	0.17	0.22	0.2	0.14	**0.37**
Wine	0.49	0.48	0.4	**0.8**	0.46	0.44	0.38	0.26	0.41	0.39	0.28	0.25	0.32	0.46
Yeastgalactose	0.59	0.48	**0.72**	0.41	0.53	0.66	0.46	0.16	0.39	0.48	0.54	0.67	0.6	0.54
Zoo	0.18	0.56	0.49	0.54	0.22	0.48	0.16	0.62	0.52	**0.63**	0.27	0.4	0.43	0.14
Synthetic	0.12 ± 0.15	0.11 ± 0.17	0.14 ± 0.16	0.24 ± 0.16	0.09 ± 0.14	0.16 ± 0.18	0.20 ± 0.12	0.10 ± 0.11	0.21 ± 0.14	0.10 ± 0.13	**0.31 ± 0.14**	0.15 ± 0.16	0.06 ± 0.1	0.06 ± 0.05
	0.24	0.29	0.32	0.33	0.27	**0.34**	0.26	0.3	0.29	0.33	0.27	0.25	0.3	0.29

Table 28: Detailed results of the ensemble experiments with members selected using different approaches (ROC AUC).

ROC AUC	Naive	Cross-validation	SDS	Perturbation	Uniform Objects
Abalone	0.75	**0.76**	0.71	0.72	0.72
Arrhythmia	**0.74**	**0.74**	**0.74**	**0.74**	**0.74**
Artificial-characters	0.89	**0.9**	0.89	0.89	0.89
Balancescale	0.87	**0.93**	0.86	0.86	0.87
Ball-bearing	0.98	**1**	0.98	0.98	0.98
Biomed	0.76	**0.79**	0.64	0.64	0.66
Breast	**0.99**	**0.99**	0.96	0.93	0.96
Cancer	**0.72**	**0.72**	0.67	0.65	0.67
Car-evaluation	0.99	**1**	0.99	0.98	0.98
Cardiotocography	0.78	**0.96**	0.76	0.78	0.75
Cellcycle	0.76	**0.79**	0.7	0.72	0.77
Cnae	0.79	**0.82**	0.74	0.75	0.76
Colon	0.78	0.77	0.73	0.78	**0.81**
Delft	0.96	**0.97**	0.95	0.95	0.91
Dermatology	0.98	**0.99**	0.98	0.98	0.98
Diabetes	0.71	**0.73**	0.7	0.7	0.71
Ecoli	**0.9**	**0.9**	0.89	0.88	0.88
Flare	0.8	**0.81**	0.77	0.79	0.77
Glass	0.73	**0.82**	0.76	0.8	0.77
Hayes-roth	0.68	**0.73**	0.67	0.59	0.62
Heart	0.63	**0.9**	0.62	0.63	0.62
Hepatitis	0.51	**0.76**	0.55	0.53	0.49
Housing	0.76	**0.83**	0.76	0.75	0.74
Imports	**0.88**	0.87	0.66	0.69	0.66
Ionosphere	**0.98**	**0.98**	**0.98**	0.96	0.95
Iris	**1**	**1**	**1**	0.99	0.99
Led	**0.93**	0.9	**0.93**	0.92	**0.93**
Lung-cancer	0.75	**0.78**	0.75	0.73	0.75
Multiple-features	**0.99**	**0.99**	**0.99**	**0.99**	0.98
Optdigits	**1**	**1**	**1**	**1**	**1**
Pageblocks	0.91	**0.95**	0.82	0.89	0.9
Sat	**0.96**	**0.96**	0.95	0.95	0.95
Seeds	0.98	**0.99**	**0.99**	**0.99**	**0.99**
Semeion	**0.97**	**0.97**	**0.97**	0.96	0.96
Sonar	**0.75**	**0.75**	0.68	0.67	0.6
Soybean-small	**1**	**1**	**1**	**1**	**1**
Spectf	0.84	0.84	0.84	**0.85**	0.84
Survival	0.76	**0.77**	0.7	0.7	0.68
Systhetic-control	**0.99**	**0.99**	**0.99**	0.98	0.98
Texture	**1**	**1**	**1**	0.99	0.99
User-knowledge	0.94	**0.95**	0.94	0.93	0.94
Vehicle	0.81	**0.86**	0.81	0.8	0.8
Vertebra-column	**0.9**	**0.9**	**0.9**	**0.9**	0.89
Vowels	**1**	**1**	**1**	0.99	0.99
Waveform	0.89	**0.9**	0.88	0.89	0.89
Wine	0.85	**0.91**	0.86	0.87	0.86
Yeastgalactose	**1**	**1**	**1**	**1**	**1**
Zoo	**1**	**1**	**1**	**1**	**1**
	0.87	**0.89**	0.85	0.85	0.85

Table 29: Detailed results of the ensemble experiments with members selected using different approaches (AdjustedPrec@n).

AdjustedPrec@n	Naive	Cross-validation	SDS	Perturbation	Uniform Objects
Abalone	0.34	**0.35**	0.3	0.32	0.31
Arrhythmia	0.36	0.36	**0.41**	**0.41**	**0.41**
Artificial-characters	0.36	**0.4**	0.36	0.36	0.37
Balancescale	0.47	**0.68**	0.48	0.45	0.46
Ball-bearing	0.91	**0.97**	0.89	0.9	0.89
Biomed	0.53	**0.59**	0.24	0.24	0.24
Breast	**0.91**	**0.91**	0.85	0.74	0.83
Cancer	0.28	**0.35**	0.28	0.28	0.21
Car-evaluation	0.87	**0.88**	0.82	0.8	0.8
Cardiotocography	0.34	**0.76**	0.32	0.36	0.31
Cellcycle	**0.43**	0.36	0.38	0.36	**0.43**
Cnae	0.46	**0.5**	0.32	0.34	0.39
Colon	**0.25**	**0.25**	**0.25**	**0.25**	**0.25**
Delft	0.72	**0.79**	0.74	0.7	0.61
Dermatology	0.84	**0.89**	0.84	0.82	0.8
Diabetes	0.28	0.28	**0.31**	**0.31**	**0.31**
Ecoli	**0.76**	**0.76**	**0.76**	0.65	**0.76**
Flare	**0.44**	0.4	0.43	0.41	0.43
Glass	0.33	**0.38**	0.33	0.33	0.31
Hayes-roth	0.15	**0.25**	0.15	0.06	0.16
Heart	0.18	**0.62**	0.18	0.18	0.18
Hepatitis	-0.25	**0.17**	-0.25	-0.25	-0.25
Housing	0.21	**0.33**	0.21	0.21	0.21
Imports	**0.61**	**0.61**	0.22	0.22	0.22
Ionosphere	**0.81**	**0.81**	**0.81**	**0.81**	0.75
Iris	**0.95**	**0.95**	**0.95**	**0.95**	**0.95**
Led	**0.58**	0.48	0.48	0.48	**0.58**
Lung-cancer	**0.47**	**0.47**	**0.47**	**0.47**	**0.47**
Multiple-features	0.88	**0.89**	0.88	0.88	0.87
Optdigits	0.94	**0.95**	0.94	0.94	0.93
Pageblocks	0.46	**0.6**	0.29	0.45	0.4
Sat	0.77	**0.78**	0.74	0.74	0.73
Seeds	0.86	**0.93**	0.89	0.89	0.89
Semeion	0.8	**0.81**	0.8	0.77	**0.81**
Sonar	**0.36**	**0.36**	0.22	0.17	0.07
Soybean-small	**1**	**1**	**1**	**1**	**1**
Spectf	**0.43**	**0.43**	**0.43**	**0.43**	**0.43**
Survival	0.36	**0.41**	0.27	0.23	0.27
Systhetic-control	0.88	**0.94**	0.88	0.85	0.86
Texture	0.95	**0.96**	0.93	0.92	0.91
User-knowledge	**0.63**	**0.63**	0.61	0.57	0.54
Vehicle	0.47	**0.48**	0.47	0.45	0.43
Vertebra-column	**0.58**	0.55	0.56	**0.58**	0.56
Vowels	**0.96**	**0.96**	0.93	0.91	0.85
Waveform	0.58	**0.6**	0.56	0.55	0.56
Wine	0.43	**0.71**	0.47	0.57	0.61
Yeastgalactose	0.88	**0.97**	0.88	**0.97**	**0.97**
Zoo	**1**	**1**	**1**	**1**	**1**
	0.58	**0.64**	0.55	0.54	0.54

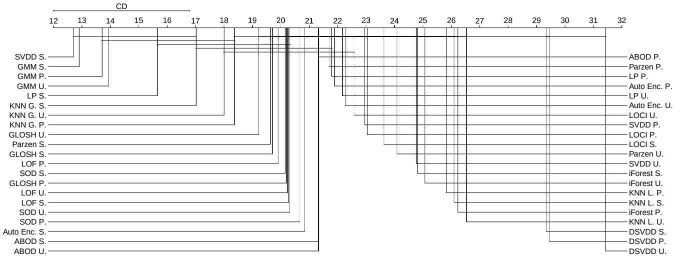

(a) ROC AUC

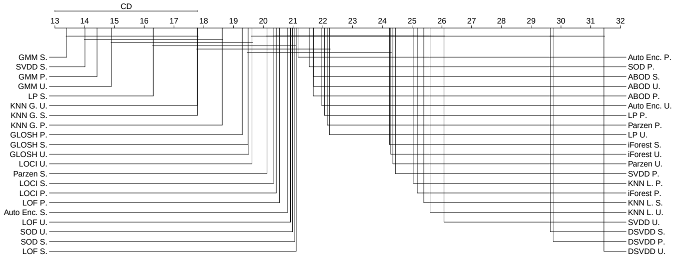

(b) AdjustedPrec@n

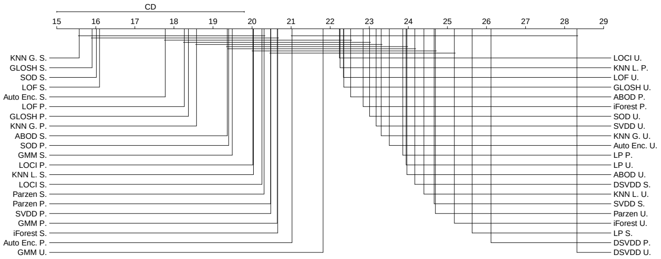

(c) MCC

Fig. 3: Complete critical difference diagrams with average ranks of the methods equipped with their best parameter value according to the respective model selection method. Methods ending in S., P. and U. had their parameters selected by SDS, Perturbation, and Uniform Objects, respectively

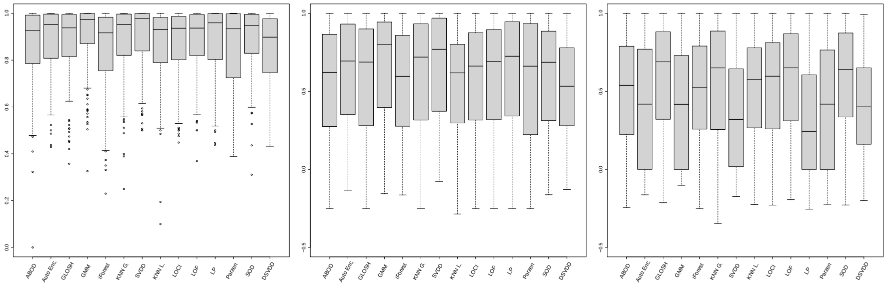

(a) ROC AUC (b) AdjustedPrec@n (c) MCC

Fig. 4: Boxplot of the results of the methods with the parameters selected using Cross-validation over all datasets of the Type I experiments (Single Source-Class Inliers)

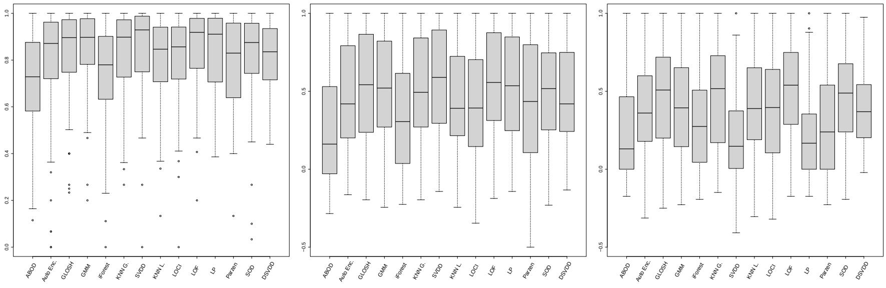

(a) ROC AUC (b) AdjustedPrec@n (c) MCC

Fig. 5: Boxplot of the results of the methods with the parameters selected using Cross-validation over all datasets of the Type II experiments (Multiple Source-Class Inliers)

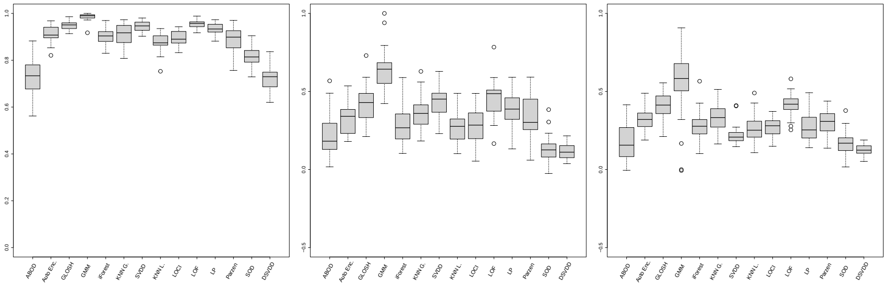

(a) ROC AUC (b) AdjustedPrec@n (c) MCC

Fig. 6: Boxplot of the results of the methods with the parameters selected using Cross-validation over all datasets of the Type III experiments (Local Outliers)

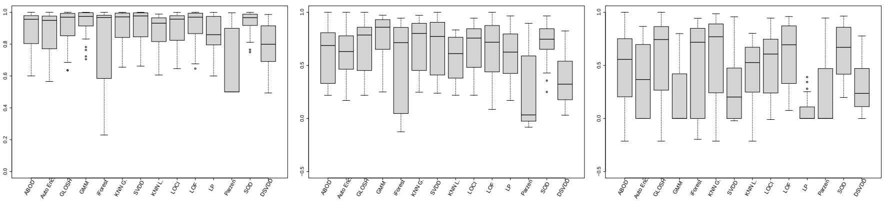

(a) ROC AUC (b) AdjustedPrec@n (c) MCC

Fig. 7: Boxplot of the results of the methods with the parameters selected using Cross-validation over all datasets of the Type IV experiments (High-Dimensional Datasets)

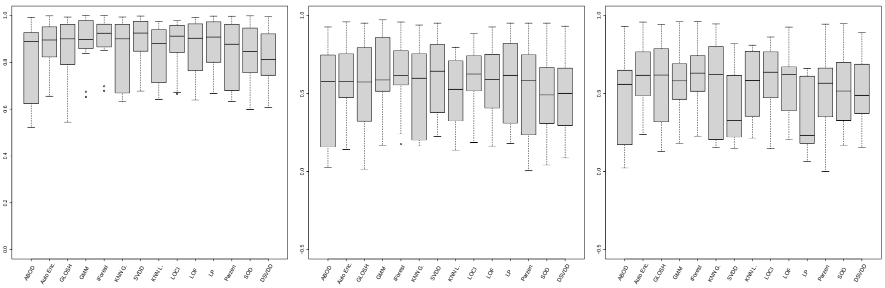

(a) ROC AUC (b) AdjustedPrec@n (c) MCC

Fig. 8: Boxplot of the results of the methods with the parameters selected using Cross-validation over all datasets of the Type V experiments (Larger Sample Size)

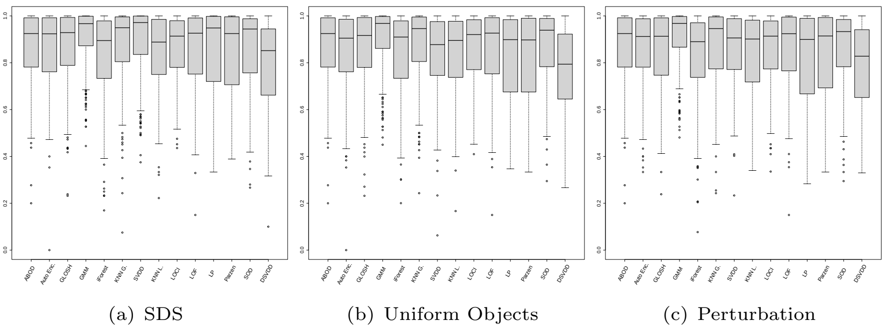

(a) SDS (b) Uniform Objects (c) Perturbation

Fig. 9: Boxplot of the ROC AUC results of the methods with the parameters selected using different practical model selection methods over all datasets of the Type I experiments (Single Source-Class Inliers)

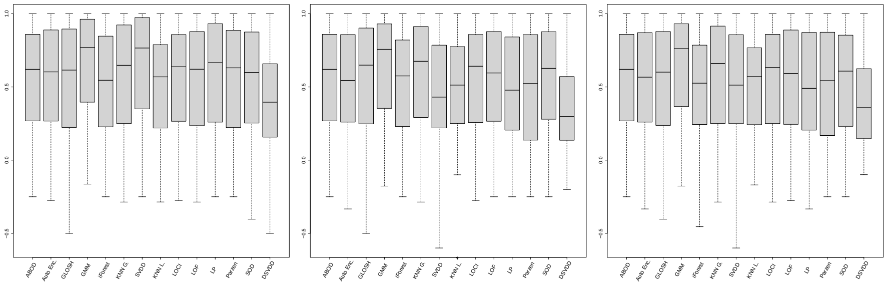

(a) SDS (b) Uniform Objects (c) Perturbation

Fig. 10: Boxplot of the AdjustedPrec@n results of the methods with the parameters selected using different practical model selection methods over all datasets of the Type I experiments (Single Source-Class Inliers)

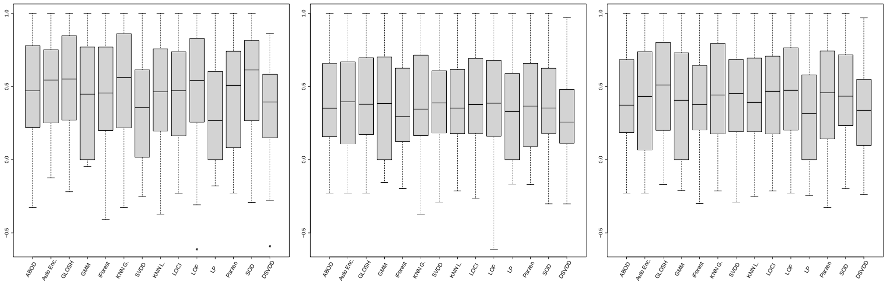

(a) SDS (b) Uniform Objects (c) Perturbation

Fig. 11: Boxplot of the MCC results of the methods with the parameters selected using different practical model selection methods over all datasets of the Type I experiments (Single Source-Class Inliers)

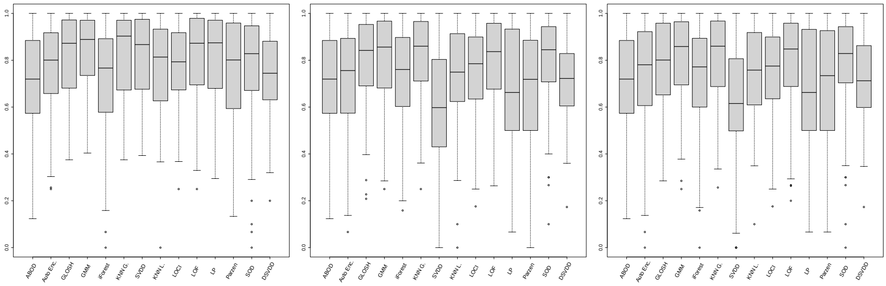

(a) SDS (b) Uniform Objects (c) Perturbation

Fig. 12: Boxplot of the ROC AUC results of the methods with the parameters selected using different practical model selection methods over all datasets of the Type II experiments (Multiple Source-Class Inliers)

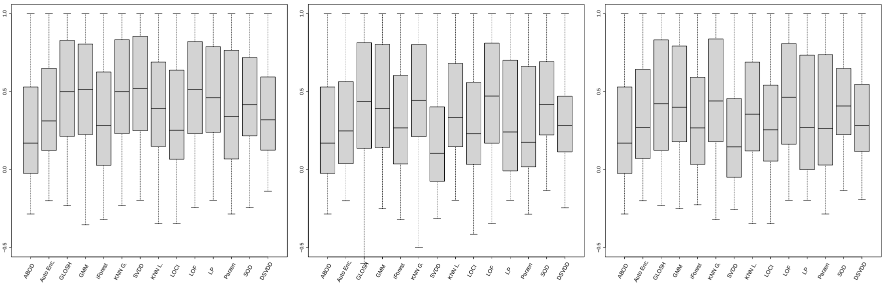

(a) SDS (b) Uniform Objects (c) Perturbation

Fig. 13: Boxplot of the AdjustedPrec@n results of the methods with the parameters selected using different practical model selection methods over all datasets of the Type II experiments (Multiple Source-Class Inliers)

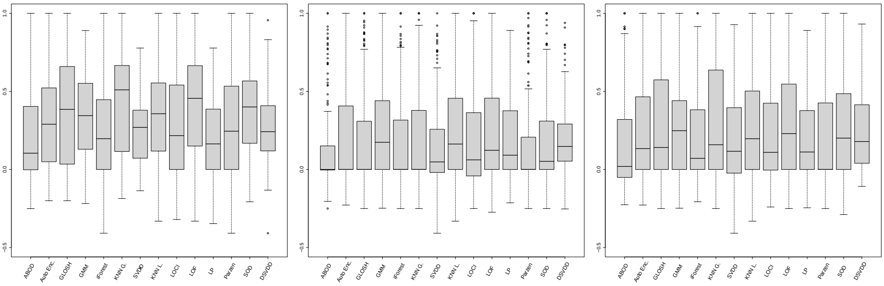

(a) SDS (b) Uniform Objects (c) Perturbation

Fig. 14: Boxplot of the MCC results of the methods with the parameters selected using different practical model selection methods over all datasets of the Type II experiments (Multiple Source-Class Inliers)

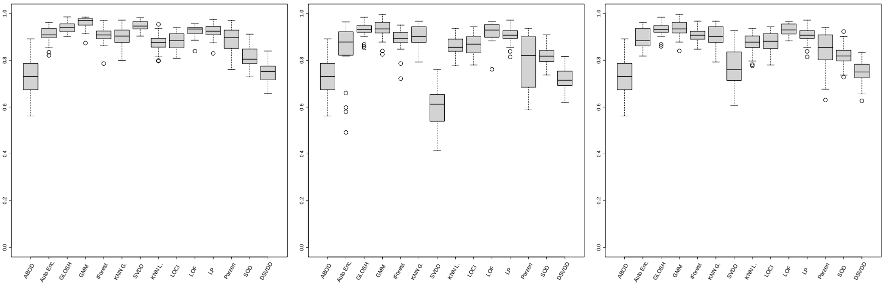

(a) SDS (b) Uniform Objects (c) Perturbation

Fig. 15: Boxplot of the ROC AUC results of the methods with the parameters selected using different practical model selection methods over all datasets of the Type III experiments (Local Outliers)

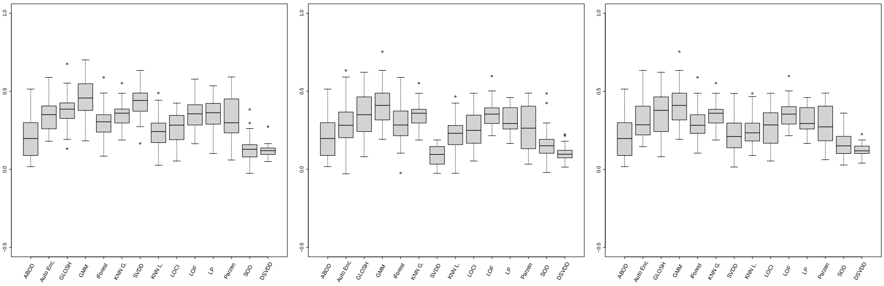

(a) SDS (b) Uniform Objects (c) Perturbation

Fig. 16: Boxplot of the AdjustedPrec@n results of the methods with the parameters selected using different practical model selection methods over all datasets of the Type III experiments (Local Outliers)

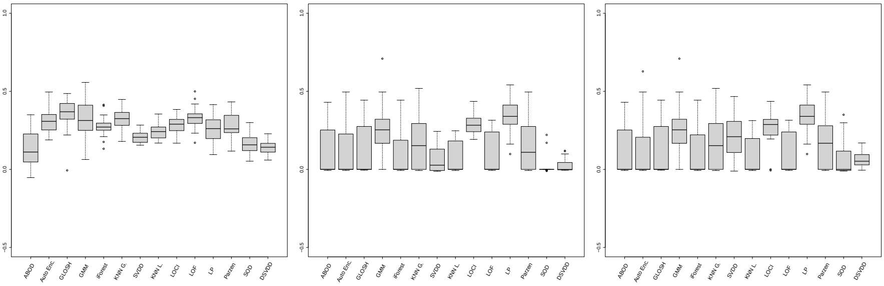

(a) SDS (b) Uniform Objects (c) Perturbation

Fig. 17: Boxplot of the MCC results of the methods with the parameters selected using different practical model selection methods over all datasets of the Type III experiments (Local Outliers)

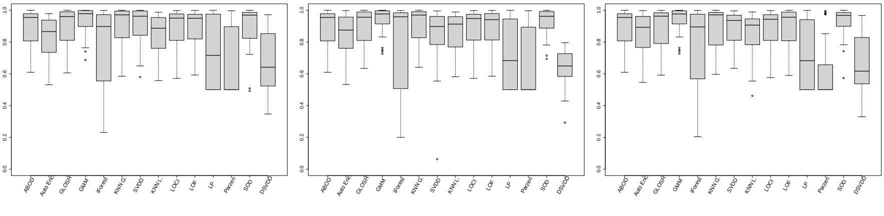

(a) SDS (b) Uniform Objects (c) Perturbation

Fig. 18: Boxplot of the ROC AUC results of the methods with the parameters selected using different practical model selection methods over all datasets of the Type IV experiments (High-Dimensional Datasets)

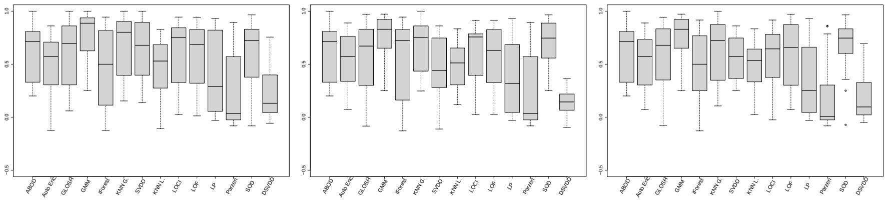

(a) SDS (b) Uniform Objects (c) Perturbation

Fig. 19: Boxplot of the AdjustedPrec@n results of the methods with the parameters selected using different practical model selection methods over all datasets of the Type IV experiments (High-Dimensional Datasets)

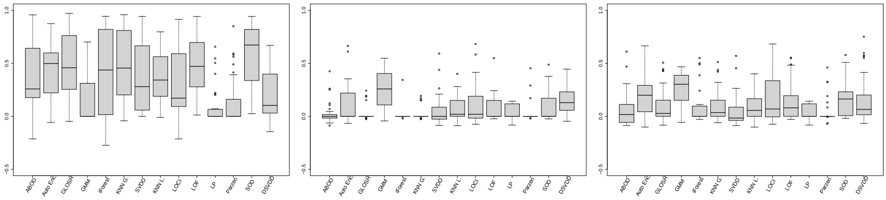

(a) SDS (b) Uniform Objects (c) Perturbation

Fig. 20: Boxplot of the MCC results of the methods with the parameters selected using different practical model selection methods over all datasets of the Type IV experiments (High-Dimensional Datasets)

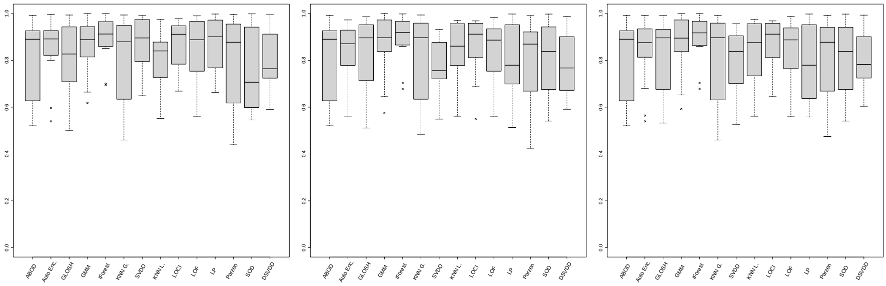

(a) SDS (b) Uniform Objects (c) Perturbation

Fig. 21: Boxplot of the ROC AUC results of the methods with the parameters selected using different practical model selection methods over all datasets of the Type V experiments (Larger Sample Size)

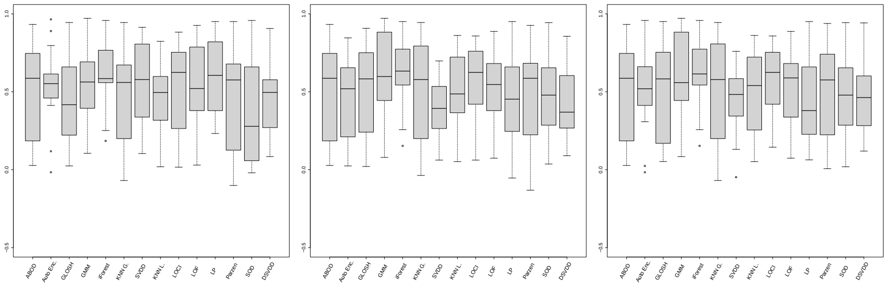

(a) SDS (b) Uniform Objects (c) Perturbation

Fig. 22: Boxplot of the AdjustedPrec@n results of the methods with the parameters selected using different practical model selection methods over all datasets of the Type V experiments (Larger Sample Size)

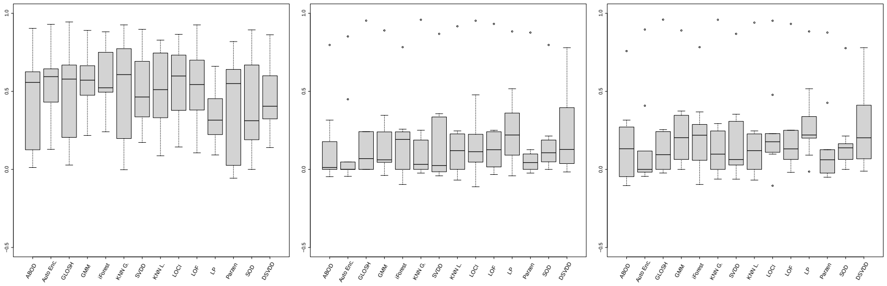

(a) SDS (b) Uniform Objects (c) Perturbation

Fig. 23: Boxplot of the MCC results of the methods with the parameters selected using different practical model selection methods over all datasets of the Type V experiments (Larger Sample Size)

References

Bazaraa MS, Jarvis JJ, Sherali HD (2009) Linear Programming and Network Flows, 4th edn. Wiley-Interscience

Bishop CM (2007) Pattern Recognition and Machine Learning, 5th edn. Springer

Breunig MM, Kriegel H, Ng RT, et al (2000) LOF: identifying density-based local outliers. In: Proceedings of the 2000 SIGMOD International Conference on Management of Data. ACM, pp 93–104, https://doi.org/10.1145/342009.335388

Campbell C, Bennett KP (2000) A linear programming approach to novelty detection. In: Proceedings of the 13th NIPS International Conference on Neural Information Processing Systems. MIT Press, pp 395–401

Campello RJGB, Moulavi D, Zimek A, et al (2015) Hierarchical density estimates for data clustering, visualization, and outlier detection. ACM Trans Knowl Discov Data 10(1):5:1–5:51. https://doi.org/10.1145/2733381

Dempster AP, Laird NM, Rubin DB (1977) Maximum likelihood from incomplete data via the em algorithm. Journal of the Royal Statistical Society 39(1):1–38

Duin RPW (1976) On the choice of smoothing parameters for parzen estimators of probability density functions. IEEE Trans Computers 25(11):1175–1179. https://doi.org/10.1109/TC.1976.1674577

Erfani SM, Baktashmotlagh M, Rajasegarar S, et al (2015) R1SVM: A randomised nonlinear approach to large-scale anomaly detection. In: Proceedings of the 29th AAAI Conference on Artificial Intelligence. AAAI Press, pp 432–438

Freeman JA, Skapura DM (1991) Neural Networks - Algorithms, Applications, and Programming Techniques. Addison-Wesley

Friedman JH, Bentley JL, Finkel RA (1977) An algorithm for finding best matches in logarithmic expected time. ACM Trans Math Softw 3(3):209–226. https://doi.org/10.1145/355744.355745

Goodfellow IJ, Bengio Y, Courville AC (2016) Deep Learning. MIT Press

Japkowicz N, Myers C, Gluck MA (1995) A novelty detection approach to classification. In: Proceedings of the 4th IJCAI International Joint Conference on Artificial Intelligence. Morgan Kaufmann, pp 518–523

Joachims T (2006) Training linear SVMs in linear time. In: Proceedings of the 12th SIGKDD International Conference on Knowledge Discovery and Data Mining. ACM, pp 217–226, https://doi.org/10.1145/1150402.1150429

Kriegel H, Schubert M, Zimek A (2008) Angle-based outlier detection in high-dimensional data. In: Proceedings of the 14th SIGKDD International Conference on Knowledge Discovery and Data Mining. ACM, pp 444–452, https://doi.org/10.1145/1401890.1401946

Kriegel H, Kröger P, Schubert E, et al (2009) Outlier detection in axis-parallel subspaces of high dimensional data. In: Proceedings of the 13th PAKDD Pacific-Asia Conference on Knowledge Discovery and Data Mining. Springer, pp 831–838, https://doi.org/10.1007/978-3-642-01307-2_86

LeCun Y, Bottou L, Orr GB, et al (1998) Efficient BackProp. In: Montavon G, Orr GB, Müller K (eds) Neural Networks: Tricks of the Trade. Springer, p 9–50, https://doi.org/10.1007/3-540-49430-8_2

Liu FT, Ting KM, Zhou Z (2008) Isolation forest. In: Proceedings of the 8th ICDM International Conference on Data Mining. IEEE Computer Society, pp 413–422, https://doi.org/10.1109/ICDM.2008.17

Liu FT, Ting KM, Zhou Z (2012) Isolation-based anomaly detection. ACM Trans Knowl Discov Data 6(1):3:1–3:39. https://doi.org/10.1145/2133360.2133363

Papadimitriou S, Kitagawa H, Gibbons PB, et al (2003) LOCI: fast outlier detection using the local correlation integral. In: Proceedings of the 19th ICDE International Conference on Data Engineering. IEEE Computer Society, pp 315–326, https://doi.org/10.1109/ICDE.2003.1260802

Parzen E (1962) On estimation of a probability density function and mode. The annals of mathematical statistics 33(3):1065–1076

Pekalska E, Tax DMJ, Duin RPW (2002) One-class LP classifiers for dissimilarity representations. In: Proceedings of the 15th NIPS International Conference on Neural Information Processing Systems, Advances in Neural Information Processing Systems. MIT Press, pp 761–768

Ramaswamy S, Rastogi R, Shim K (2000) Efficient algorithms for mining outliers from large data sets. In: Proceedings of the 2000 SIGMOD International Conference on Management of Data. ACM, pp 427–438, https://doi.org/10.1145/342009.335437

de Ridder D, Tax DMJ, Duin RPW (1998) An experimental comparison of one-class classification methods. In: Proceedings of the 4th ASCI Advanced School for Computing and Imaging, pp 213–218

Roussopoulos N, Kelley S, Vincent F (1995) Nearest neighbor queries. In: Proceedings of the 1995 SIGMOD International Conference on Management of Data. ACM Press, pp 71–79, https://doi.org/10.1145/223784.223794

Ruff L, Görnitz N, Deecke L, et al (2018) Deep one-class classification. In: Proceedings of the 35th ICML International Conference on Machine Learning. PMLR, pp 4390–4399

Schölkopf B, Platt JC, Shawe-Taylor J, et al (2001) Estimating the support of a high-dimensional distribution. Neural Comput 13(7):1443–1471. https://doi.org/10.1162/089976601750264965

Strang G (2016) Introduction to Linear Algebra, 5th edn. Wellesley-Cambridge Press

Tax DMJ (2001) One-class classification. PhD thesis, Delft University of Technology

Tax DMJ, Duin RPW (2004) Support vector data description. Mach Learn 54(1):45–66. https://doi.org/10.1023/B:MACH.0000008084.60811.49

Vapni VN (1995) The Nature of Statistical Learning Theory. Springer, https://doi.org/10.1007/978-1-4757-2440-0

Webb GI (2011) Lazy learning. In: Sammut C, Webb GI (eds) Encyclopedia of Machine Learning. Springer, p 571–572, https://doi.org/10.1007/978-0-387-30164-8_443

Woodsend K (2009) Using interior point methods for large-scale support vector machine training. PhD thesis, University of Edinburgh
